# Insights into respiratory illness at the population level through parallel analysis of pharmaceutical and viral markers in wastewater

**DOI:** 10.1038/s44221-025-00437-4

**Published:** 2025-05-14

**Authors:** Stephan Baumgartner, Michelle Salvisberg, Patrick Schmidhalter, Timothy R. Julian, Christoph Ort, Heinz Singer

**Affiliations:** 1 Eawag, Swiss Federal Institute of Aquatic Science and Technology, Dubendorf, Switzerland; 2 Institute of Civil, Environmental and Geomatic Engineering, ETH Zürich, Zurich, Switzerland; 3 Swiss Tropical and Public Health Institute, Allschwil, Switzerland; 4 University of Basel, Basel, Switzerland

**Keywords:** Infectious diseases, Biomarkers, Environmental sciences

## Abstract

Wastewater as a medium contains information on both circulating pathogens and drug consumption at the population level. This study combines tracking of respiratory viruses and quantification of pharmaceuticals as untargeted indicators of symptoms related to acute respiratory infections and influenza-like illnesses such as coughing, fever and pain. From January 2021 to June 2024, raw wastewater samples from ten locations covering 23% of the Swiss population were analysed. This encompassed 15 pharmaceuticals and four priority respiratory viruses including severe acute respiratory syndrome coronavirus virus-2 (SARS-CoV-2), respiratory syncytial virus (RSV), influenza A and influenza B viruses. The pharmaceutical compounds dextromethorphan, pheniramine, clarithromycin, acetaminophen and codeine showed a strong correlation with respiratory virus loads in wastewater. This enabled the estimation of pathogen-specific and cumulative symptom treatment in the population. In 2021 and 2024, notable increases in pharmaceutical loads without corresponding increases in viral loads signalled high community symptoms linked to unsurveilled pathogens. This study demonstrates that pharmaceutical surveillance can inform respiratory disease burden and highlights the value of integrated surveillance for assessing emerging public health threats beyond those routinely monitored.

## Abstract (web summary)

Wastewater surveillance is used to monitor pharmaceuticals related to respiratory diseases in parallel with four respiratory viruses. The results demonstrate the potential of pharmaceutical surveillance to assess emerging public health threats.

## Main

Wastewater contains a diverse array of human excreta, offering a valuable source of epidemiological information at the population level. Historically, wastewater-based surveillance (WBS) has evolved along two relatively distinct paths: one focused on the analysis of small molecules—such as illicit drugs, pharmaceuticals and lifestyle chemicals—to estimate community-level substance use^1^, and the other centered on monitoring infectious diseases through the analysis of pathogens in wastewater^2^. The detection of severe acute respiratory syndrome coronavirus-2 (SARS-CoV-2) RNA in wastewater—which showed strong correlations with clinical cases—marked a rapid uptake in WBS for pathogen surveillance globally^3^. The rigorous, simultaneous monitoring of SARS-CoV-2 in both clinical settings and wastewater provided a unique opportunity to validate WBS as an objective, inclusive and cost-effective tool for public health monitoring. As WBS has gained recognition and institutionalization, its scope has expanded to cover a wide range of pathogens beyond SARS-CoV-2, including additional respiratory viruses, gastrointestinal pathogens, hepatitis and monkeypox virus^4–10^.

In addition to tracking pathogens, WBS was applied to chemicals during the pandemic to examine shifts in the consumption of illicit drugs and pharmaceuticals, illustrating the widespread impacts of the pandemic on lifestyle and emotional health^11–14^.

Although chemicals and pathogens were investigated extensively, combined analyses are rare and have not been integrated into extended surveillance efforts^15–17^. However, pharmaceutical analysis, offers a promising avenue for the assessment of the level of symptom treatment in a population, as a proxy for the disease burden. One example of this is seasonal allergic rhinitis (commonly referred to as hay fever). Longitudinal wastewater-based antihistamine surveillance has demonstrated its utility in tracking symptom dynamics over time, providing valuable insights into the population burden of allergenic triggers^18^. Similarly, in the context of respiratory illnesses, pharmaceutical loads in wastewater offer rapid feedback on the population’s response during infectious disease outbreaks, complementing the highly targeted nucleic acid quantification assays of priority pathogens. This untargeted approach enables the estimation of symptom burdens for monitored pathogens and the detection of emerging, unsurveilled threats, such as ‘Pathogen X’^19–21^. Additionally, the parallel analysis of chemical and microbial targets in the same wastewater sample ensures that both pieces of information originate simultaneously from the same, large group of individuals on any given day. This facilitates circumventing inherent challenges in comparing and interpreting data that arise from unknown or difficult to quantify variations in (1) population dynamics (how many and which individuals contribute to a given wastewater sample) and (2) epidemiological data (temporal changes due to varying willingness to test and testing capacity, particularly evident during the pandemic).

In this study, chemical surveillance focused on pharmaceuticals used to alleviate symptoms of acute respiratory infections and influenza-like illnesses, specifically coughing, fever and pain. We analysed wastewater samples from ten wastewater treatment plants (WWTPs) serving approximately 23% of the Swiss population, collected from 2021 to mid-2024. Pharmaceutical surveillance was complemented by wastewater measurements for priority respiratory viruses including SARS-CoV-2, respiratory syncytial virus (RSV), influenza A virus (IAV) and influenza B virus (IBV). These viruses are recognized as leading causes of respiratory illnesses and impose substantial health and economic burdens on communities^22–24^. Furthermore, the data were compared with national-level sentinel surveillance to explore additional respiratory viruses as explanatory variables for pharmaceutical consumption.

With this analysis we aimed to:

1. Quantify the dynamics of pharmaceutical consumption in wastewater as an indicator of symptomatic surveillance
2. Examine the relationship between symptomatic treatment and phases of respiratory viral exposure to better understand population-level symptom burdens
3. Identify periods of high levels of treatment without corresponding aetiologies to highlight potential contributions from unsurveilled pathogens

## Parallel WBS of drugs and viruses

Wastewater samples were collected from ten WWTPs throughout Switzerland, representing diverse geographic and socioeconomic contexts, including Zurich, Basel, Bern, Solothurn, Chur, Schwyz (German-speaking), Geneva, Lausanne, Neuchâtel (French-speaking) and Lugano (Italian-speaking). These WWTPs cover the five largest cities as well as rural areas, with catchment populations ranging from 31,000 to 471,000 individuals, collectively serving around 2.02 million people (Supplementary Fig. 1). Chemical analysis was performed on samples collected every 13th day to ensure that each weekday was represented once per quarter between January 2021 and June 2024.

We monitored 15 chemicals in wastewater, including the opioid analogue dextrorphan (the main urine metabolite of the cough suppressant dextromethorphan), the opioids codeine, morphine and tramadol and non-opioid analgesics acetaminophen (paracetamol), diclofenac and naproxen. Additionally, pheniramine and its metabolite *N*-desmethylpheniramine were assessed owing to its use in Switzerland, where it is found exclusively in over-the-counter combination products for treating flu and cold symptoms (Supplementary Table 12). Furthermore, the antibiotics clarithromycin (macrolide), sulfamethoxazole (sulfonamide), trimethoprim (antifolate) and metronidazole (nitroimidazole) were quantified.

These compounds were selected on the basis of their relevance in the treatment of respiratory illnesses, including symptomatic relief and associated infections, as well as their high consumption volumes in terms of mass.

Candesartan, an antihypertensive, and paraxanthine, a urine metabolite of caffeine, were included as control compounds, with their consumption expected to remain rather stable over time. Aside from utilizing a constant value for the residential population to calculate population-normalized loads and trends in wastewater, we used these two compounds to determine whether (unknown/unexpected) changes in population dynamics impact the general patterns. This is of particular importance during the transition from the pandemic to the post-pandemic phase (Supplementary Fig. 4). The patterns were found to persist regardless of the normalization approach used. Therefore, census data were used to calculate population normalized loads for further analyses in this study. Additionally, since this study focuses on the correlation of pharmaceutical and viral loads obtained from the same wastewater sample, any uncertainties from sampling, population size and flow cancel out when assessing the relationship of the population-normalized loads of pharmaceutical and viral markers (it reduces to the ratio of the two concentrations measured in the same wastewater sample—a synthetic example is given in Supplementary Fig. 5 for illustration purposes). Furthermore, we conducted in-sample stability experiments, demonstrating that the transformation of compounds is negligible, under the study’s specified conditions of 4 °C for short-term handling and −20 °C for long-term storage (Supplementary Information).

**Fig. 1:**
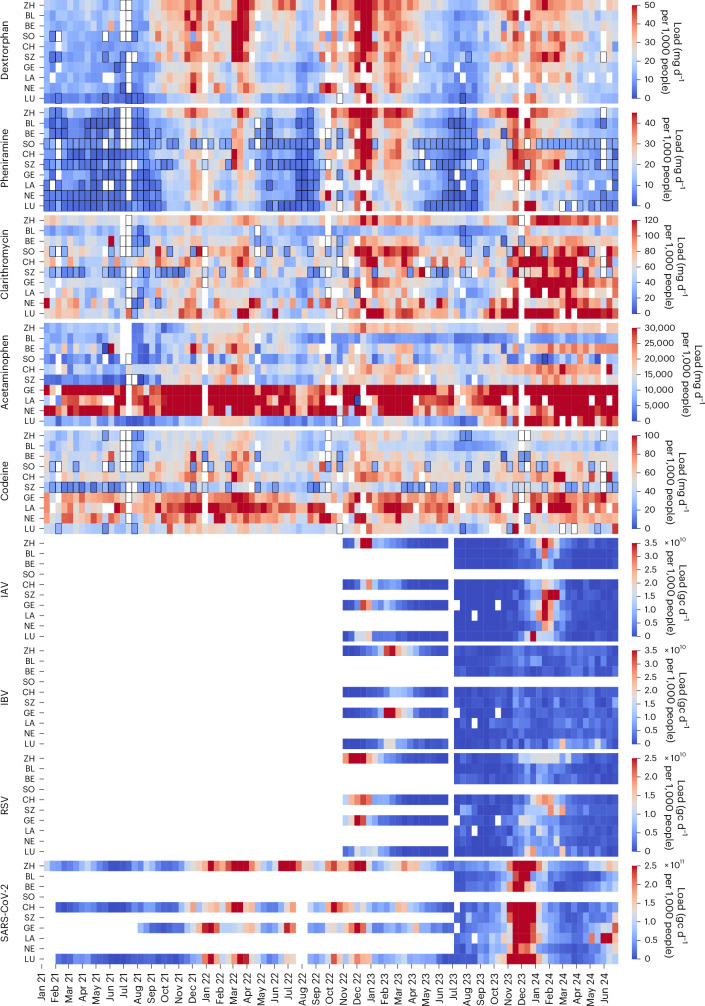
Spatiotemporal load patterns of pharmaceuticals and viral gene copies in wastewater. Wastewater loads from every 13th day between January 2021 and June 2024 are displayed. Data are shown for individual treatment plants according to the abbreviations on the *y* axis. ZH, Zurich; BL, Basel; BE, Bern; SO, Solothurn; CH, Chur; SZ, Schwyz; GE, Geneva; LA, Lausanne; NE, Neuchâtel; LU, Lugano. These are arranged first by language in the region then by catchment population size. The colouring represents the load values for pharmaceuticals (mg d^−1^ per 1,000 people) and viruses (gc d^−1^ per 1,000 people) with linear colour scaling. For pharmaceuticals, each space refers to an individual sample of a particular day, and for the viral loads, each space reflects values from the centred 7-day median. White spaces indicate data exclusions owing to rain-related increases in wastewater volumes exceeding maximum threshold values or owing to the absence of sampling. The black-outlined spaces represent pharmaceutical loads from samples with concentrations below the limit of quantification (LOQ), which are estimated on the basis of half the LOQ value.

Figure 1 displays the population-normalized loads of individual samples for the pharmaceuticals dextrorphan, pheniramine, clarithromycin, acetaminophen and codeine across the ten locations.

Pronounced seasonal trends were observed in the wastewater for several pharmaceuticals monitored. Higher loads of dextrorphan and pheniramine were typically measured during the winter months with occasional increases in summer months, such as June and July 2022. Clarithromycin followed a similar seasonal pattern from 2021 to 2023; however, its levels remained consistently elevated in the first half of 2024.

Other pharmaceuticals did not show clear seasonality with trends obscured by pronounced spatial variation in loads. Codeine levels were notably higher in the French-speaking regions of Switzerland, with a mean of 75 mg d^−1^ per 1,000 people, compared with the non-French-speaking regions, which had a mean of 45 mg d^−1^ per 1,000 people. Similarly, acetaminophen was higher in French-speaking regions with a mean of 33,531 mg d^−1^ per 1,000 people compared with 12,441 mg d^−1^ per 1,000 people in non-French-speaking regions. This disparity suggests that regional usage practises, potentially shaped by Switzerland’s decentralized healthcare system and fragmented decision-making^25^, exert a substantial influence on pharmaceutical consumption patterns.

From 2021 through late 2022, viral analysis was conducted daily for SARS-CoV-2, limited to four WWTPs. The scope expanded in late October 2022 to include RSV, IAV and IBV. Since July 2023, these four pathogens were measured in all ten WWTPs, with the frequency reduced to 5 days per week (Fig. 1).

The missing data for pharmaceuticals, indicated by white spaces, are due the exclusion of values on rainy days when the inflow volume at WWTPs exceeded an empirically determined location-specific maximum threshold (Supplementary Table 1 and Supplementary Figs. 2 and 3). Beyond this threshold, estimated loads are consistently underestimated or overestimated across the substance spectrum. This discrepancy is linked to the potential discharge of untreated wastewater through combined sewer overflows and to inaccuracies in sampling or flow measurements at the WWTPs during high flow conditions. These events usually span over several locations. In contrast, viral gene copy load data from these dates were still considered reliable, as they represent 7-day centred median values, which reduce the impact of outliers.

The compounds dextrorphan, pheniramine, clarithromycin, acetaminophen and codeine were selected for detailed analysis based on their significant positive Pearson correlation with viral loads of SARS-CoV-2, RSV and IAV both location-dependent and independent (Supplementary Figs. 15–21). While the manuscript focuses on these pharmaceuticals, analogous analyses for the additional chemicals are provided in the Supplementary Information.

## Wastewater and clinical data at the national level

For dextrorphan, pheniramine and, to a lesser extent, clarithromycin, temporal fluctuations were relatively consistent across locations, as evidenced by the narrow 10–90% inter-percentile range (Supplementary Figs. 7–9). This pattern was also observed in viral loads (Supplementary Fig. 10), indicating similar trends across the sampled locations.

**Fig. 2:**
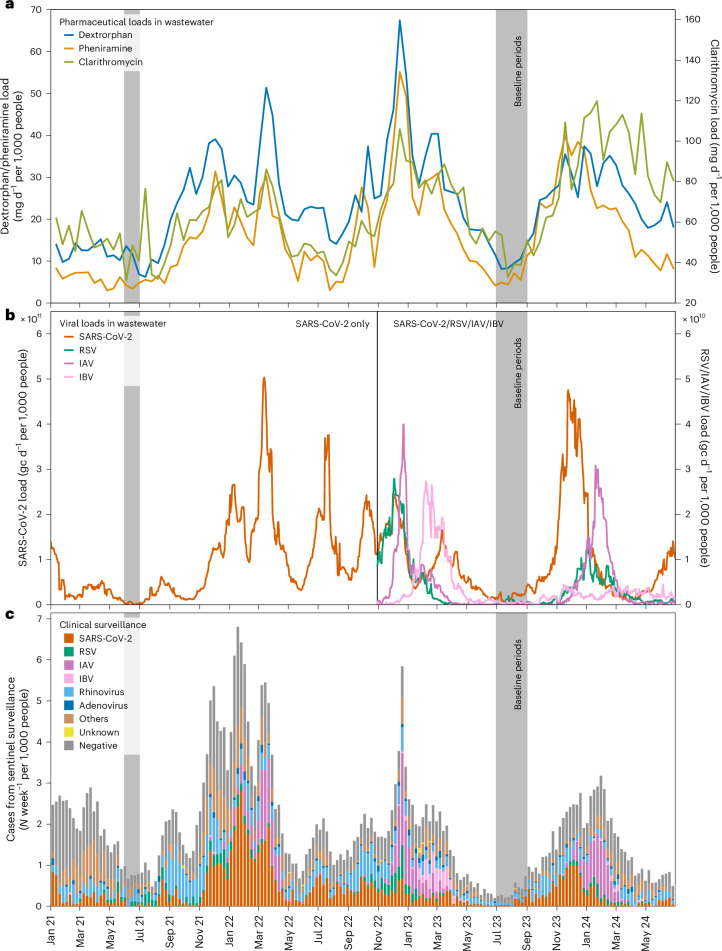
National trends of pharmaceutical and viral data in wastewater (from ten WWTPs) alongside clinical sentinel testing data (January 2021 to June 2024). **a**, The median pharmaceutical loads recorded every 13th day. **b**, The median viral gene copy loads based on the daily 7-day centred median values. **c**, The stacked testing cases from the general practitioner-based sentinel surveillance system. The ‘others’ category (shown in brown) encompasses tests for bocavirus, human coronaviruses (229E, HKU1, NL63 and OC43), metapneumovirus and parainfluenza viruses (types 1–4). The ‘negative’ category includes cases where individuals sought consultation for symptoms of acute respiratory infections or influenza-like illnesses but tested negative for the respiratory viruses under surveillance. The ‘unknown’ category includes samples with incomplete data on detected respiratory viruses.

To directly compare wastewater patterns with national-level sentinel clinical testing data for respiratory viruses (Fig. 2c), we estimated the median load values from the ten locations as indicators of pharmaceutical consumption (Fig. 2a) and viral exposure (Fig. 2b) at the national level.

The seasonal patterns for dextrorphan, pheniramine and clarithromycine align with seasonality of viral loads in wastewater. In December 2022, the peak loads of dextrorphan and pheniramine coincided with a SARS-CoV-2 wave and the highest recorded loads of RSV and IAV, highlighting a substantial cumulative viral burden on the population. Subsequently, pharmaceutical loads surged again in February 2023 during an Influenza B wave, presumably the Victoria subtype (Supplementary Fig. 12).

In the following winter (2023/2024), the major SARS-CoV-2 wave in November 2023 preceded the RSV and IAV waves, mirroring concurrent peaks in pharmaceutical consumption. Notably, IBV was almost absent in winter in 2023/2024. Between 2021 and October 2022, when only SARS-CoV-2 was monitored, pharmaceutical loads generally rose in tandem with SARS-CoV-2 waves, including during the summer surge in June 2022.

From September to November 2021, an intriguing increase in pharmaceutical loads, particularly dextrorphan, occurred despite relatively low SARS-CoV-2 levels in wastewater. To expand the analysis beyond respiratory pathogens included in wastewater surveillance, we also investigated trends in the publicly available Swiss Sentinel System (Sentinella), a voluntary reporting system with contributions from 160 to 180 general practitioners and paediatricians^26^. The sentinel surveillance includes monitoring of additional respiratory viruses, such as rhinoviruses and adenoviruses, alongside seasonal human coronaviruses, bocavirus, human metapneumovirus and parainfluenza viruses (types 1–4), which are grouped under ‘others’ in Fig. 2c. In addition to pathogens, Sentinella tracks the number of individuals who sought medical consultation owing to symptoms but tested negative for the monitored viruses, suggesting the presence of alternative aetiological factors (the ‘negative’ category). The summed values from sentinel testing reflects the number of primary consultations for acute respiratory infection or influenza-like illness symptoms (Supplementary Fig. 13). These consultation counts are multiplied by testing positivity rates (Supplementary Fig. 14) to calculate weekly virus-specific cases displayed in Fig. 2c. During the period of October to November 2021, an increased number of consultations were noted, predominantly testing positive for rhinovirus and ‘others’. This period was closely followed by an observed increase in SARS-CoV-2 cases.

Periods marked by low viral exposure for SARS-CoV-2, RSV, IAV and IBV, as indicated in wastewater and sentinel data from early 2021 and mid-2023, are highlighted with grey bars (Fig. 2). To accommodate spatial variation in pharmaceutical loads, particularly for acetaminophen and codeine, location-specific mean loads from these low-activity periods were utilized to establish baselines for subsequent modelling of the influence of respiratory viruses on pharmaceutical consumption.

## Viral pathogen contributions to pharmaceutical consumption

We employed a linear model to estimate the contributions of SARS-CoV-2, RSV, IAV and IBV to pharmaceutical consumption, accounting for location-specific baselines at each WWTP. Using ordinary least squares (OLS) regression, we assessed the linear relationship between viral gene copy loads and baseline-subtracted pharmaceutical loads, under the assumption that this relationship remains consistent across locations and time. An empirical baseline, derived from mean loads during periods of low respiratory virus activity (June 2021 and July to August 2023; Fig. 2), was used. This approach was chosen instead of estimating a baseline term within the OLS model to prevent the potential obscuring of additional, unsurveilled symptom triggers by artificially reducing the sum of the squared errors.

Diagnostics of the OLS modelling are available in the Supplementary Information (Supplementary Figs. 22–26 and Supplementary Tables 5–9).

**Fig. 3:**
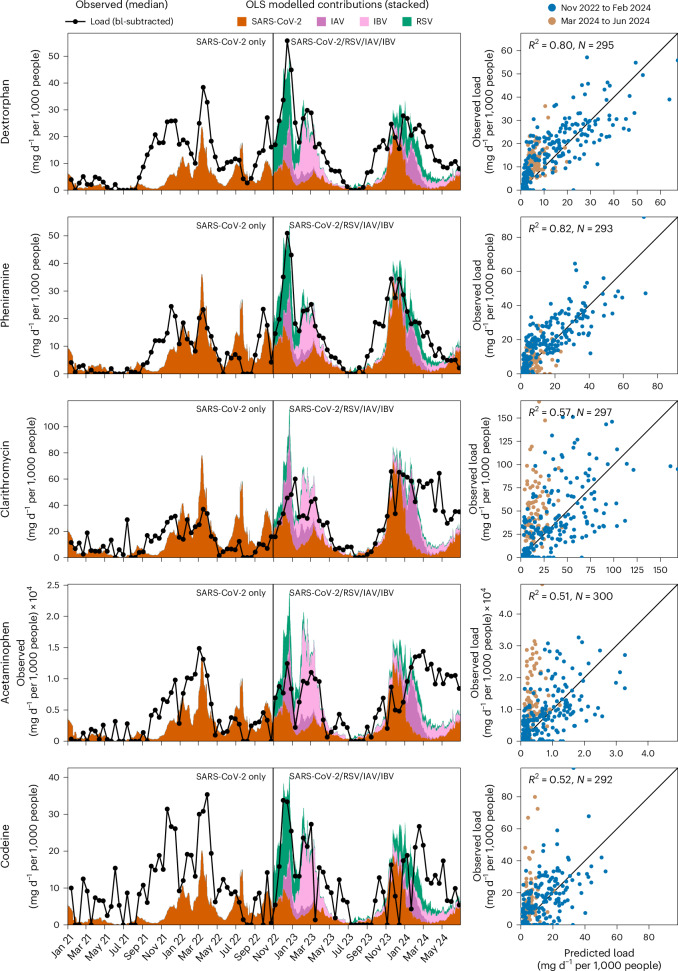
Modelled contributions of respiratory viruses to pharmaceutical loads. Left: time series plots of observed baseline-subtracted median pharmaceutical loads (black line) and the modelled contributions, derived from OLS regression coefficients applied to the median viral gene copy loads of SARS-CoV-2, RSV, IAV and IBV. The coloured areas represent the stacked contributions of each virus to the overall baseline-subtracted (bl-subtracted) pharmaceutical load. The baseline loads were determined from location-specific mean values during periods of low respiratory virus exposure (June 2021 and July to August 2023). The OLS coefficient determined for SARS-CoV-2 was applied to data from early 2021 through late October 2022, the period during which only this virus was monitored in wastewater. Right: scatter plots that compare observed versus predicted baseline-subtracted pharmaceutical loads from the OLS analyses. The data points are colour-coded by time periods: November 2022 to February 2024 (blue) and March 2024 to June 2024 (beige, indicating poor model fit with viral aetiologies). Each scatter plot includes the uncentred coefficient of determination (*R*²) and the number of observations (*N*). The diagonal black line represents the 1:1 line, indicating perfect agreement between observed and predicted values.

The OLS-estimated coefficients, applied to median viral gene copy loads, are compared with the observed median pharmaceutical loads adjusted for baselines in the left panel of Fig. 3. This comparison highlights periods where pharmaceutical loads correspond closely to respiratory viral loads and identifies discrepancies. Coloured stacked areas in the figure represent the estimated contributions of each virus from late October 2022 onwards, when monitoring included all four viruses. The coefficient determined for SARS-CoV-2 in this model was also applied to data from early 2021 through late October 2022, the period during which only this virus was monitored in wastewater.

Our analysis indicates that peaks in dextrorphan, pheniramine and clarithromycin from November 2022 to February 2024, align well with loads of SARS-CoV-2, RSV, IAV and IBV. However, patterns for acetaminophen and codeine, while elevated during these periods, exhibited spikes that did not consistently align with viral trends. Both codeine and acetaminophen are frequently recommended or used for conditions beyond infectious diseases, including managing chronic pain, osteoarthritis, menstrual pain and injuries^27,28^.

From March to June 2024, a notable discrepancy was observed, high wastewater loads of clarithromycin, acetaminophen, codeine and, to a lesser extent, dextrorphan persisted even after a decline in respiratory viruses. This inconsistency is further emphasized in the right panels of Fig. 3, where the observed versus modelled baseline-subtracted pharmaceutical loads are plotted and colour-coded by date periods. Data highlighted in beige indicate deviations from previous patterns, especially for clarithromycin, acetaminophen and codeine.

Pharmaceutical loads modelled from the beginning of 2021 to October 2022 were consistently underestimated when only SARS-CoV-2 was considered, a trend also evident in OLS models using SARS-CoV-2 as the sole predictive variable (Supplementary Fig. 29). The analyses reinforce the earlier speculation that SARS-CoV-2 alone does not adequately explain the observed pharmaceutical consumption during the period from September to November 2021.To address such gaps in pharmaceutical consumption explanation, we performed analogous OLS modelling, utilizing case data from the sentinel surveillance system. In an initial analysis we used only SARS-CoV-2, RSV, IAV and IBV cases as predictors for median baseline-subtracted loads. This provided insights similar to those obtained using wastewater viral loads but improved descriptions for 2021 and 2022 by considering additional viral waves of RSV and IAV (Supplementary Fig. 30).

Expanding these analyses to case data for rhinovirus and the ‘others’ category (comprising seasonal human coronaviruses, bocavirus, human metapneumovirus and parainfluenza viruses), we found that including rhinovirus substantially enhanced the model’s ability to describe pharmaceutical loads, particularly for dextrorphan and pheniramine (Supplementary Figs. 31–33). This was owing to rhinovirus waves preceding other respiratory viruses each year around September, which correlated with early increases in symptom treatment.

From March to June 2024, the OLS analyses showed that none of the respiratory viruses monitored by the sentinel system could account for the increased usage of clarithromycin, acetaminophen and codeine, suggesting the presence of other contributing factors beyond the surveilled respiratory virus infections.

The observed correlation between clarithromycin and viral loads may suggest its use in treating pharyngitis and pneumonia^29,30^, which can arise as complications from viral infections, including viral-bacterial co-infections or because of potential misdiagnoses^31–33^. However, clarithromycin is also prescribed for other medical conditions, such as skin infections and *Helicobacter pylori* infections^34^.

Notably, this period in 2024 coincides with a substantial increase in pertussis cases across Europe, including Switzerland, where pertussis had been nearly absent since the start of the coronavirus disease 2019 (COVID-19) pandemic in 2020^35,36^. This surge may explain the increase in clarithromycin use, as it is one of the first line macrolide antibiotics for treating pertussis^37^.

While clinical data from sentinel surveillance yield valuable insights into circulating pathogens, the quantitative estimation of infection rates is susceptible to biases introduced by under-reporting and changes in testing regimes. This issue is particularly evident in the Swiss mandatory reporting data for SARS-CoV-2 during the transition to a post-pandemic scenario (Supplementary Fig. 13). The sentinel data may experience similar biases, and further may not be nationally representative given the relatively low number of participating physicians and potential subjectivity in reporting. These biases can influence the accurate attribution of symptoms to specific viruses. In contrast, wastewater data is considered more stable over time, offering a more consistent basis for analysing trends in pathogen prevalence and corresponding pharmaceutical usage.

Overall, the OLS analyses demonstrate that the respiratory viruses SARS-CoV-2, RSV, IAV and IBV can explain most of the observed peaks in pharmaceutical usage throughout the study period, highlighting their impact on symptom burden. Additionally, the observations highlight the reliance on pharmaceuticals from infections with respiratory viruses beyond SARS-CoV-2, including IAV and RSV. Even during the pandemic in 2021, SARS-CoV-2 alone does not explain pharmaceutical use patterns, indicating a need for broader surveillance.

Furthermore, the study identifies periods when pathogens not encompassed by our wastewater surveillance — possibly including rhinovirus and pertussis — remained undetected yet prompted population-wide symptom-driven treatment. This highlights a potential method for detecting the emergence of novel pathogens in communities (‘Pathogen X’).

## Limitations

An important limitation of this study is that the current WBS of pathogens covers only a subset of possible aetiologies of disease leading to pharmaceutical use. For much of the period, only SARS-CoV-2 data from wastewater were available; however, analysis of RSV, IAV and IBV was included from late October 2022 to June 2024. Additionally, there are strong indications that other aetiological agents contribute to the population symptom burden, as exemplified by our observations on rhinovirus and pertussis. Extending the time frame of combined analyses and increasing the number of infectious diseases monitored in wastewater may help to align pharmaceutical data more accurately with disease aetiology. The current analysis may cause misattribution of symptoms caused by unsurveilled (and therefore unknown) aetiologies.

A second limitation relates to the pharmaceutical monitoring methods used. We employed a liquid chromatography–mass spectrometry technique with direct large-volume injection, which facilitated the efficient processing of numerous samples at the expense of sensitivity. More sensitive methods would improve baseline estimation and enable analysis of pharmaceuticals at lower concentrations, but these are more time-consuming and costly, potentially limiting the feasibility of high-frequency measurements.

A final limitation is in our modelling approach. Our OLS modelling assumptions may oversimplify the intricate interactions between pathogen loads and pharmaceutical usage, assuming uniform relationships across different geographical and socio-demographic contexts. Additionally, the model does not accommodate variations in disease severity, which may be tempered by vaccinations or natural immunity, nor does it distinguish between different pathogen variants or subtypes, which might have unique shedding rates and associated symptomatology^38^. Enhancing the analysis to cover extended time periods and to provide finer temporal resolution beyond the current 13-day intervals could delineate the dynamics between viral exposure and pharmaceutical consumption more accurately. Such improvements are important for assessing temporal relation of pathogen activity and drug usage.

Endogenous health biomarkers, such as inflammation markers, hold great potential as more universal indicators that could overcome the context-dependence of pharmaceuticals, though they are not yet established for WBS.

## Conclusion

This study combined chemical and microbial wastewater surveillance, analysing pharmaceuticals used to treat symptoms of acute respiratory infections and influenza-like illnesses (coughing, fever and pain) alongside major respiratory viruses. The parallel analysis established a correlation between pharmaceutical and viral wastewater loads over time, enabling an estimation of the associated disease burden. Importantly, it demonstrates the capacity to detect periods of increased health burden attributable to unsurveilled pathogens, as indicated by clinical data for rhinovirus and pertussis.

Overall, the findings highlight the value of longitudinal pharmaceutical surveillance in wastewater, providing complementary and untargeted insights into the population symptom burdens and detection of emerging diseases. Further, the study advocates for enhanced centralized coordination and interdisciplinary collaboration within chemical and microbial WBS frameworks. Standardizing procedures across sample collection, storage, analysis and data management can streamline resource use, minimize costs and ensure consistent data, which are vital for exploiting the full potential of WBS.

## Methods

### Chemicals and reagents

Details on the chemicals and solutions used for analysing the 15 chemical markers—acetaminophen, candesartan, clarithromycin, codeine, dextrorphan, diclofenac, metronidazole, morphine, *N*-desmethylpheniramine, naproxen, paraxanthine, pheniramine, sulfamethoxazole, tramadol and trimethoprim—in wastewater are provided in the Supplementary Information.

### Wastewater sample collection and storage

The sampling campaign, spanning from January 2021 to June 2024, involved the collection of 24-h composite wastewater samples from the influent of ten WWTPs across Switzerland. These samples were collected using on-site sampling infrastructure. Small molecule markers were analysed in samples collected every 13th day. Together with the daily analyses of samples from Zurich in 2021 (Supplementary Figs. 7–9), this resulted in the chemical analysis of 1,172 wastewater samples. Additionally, viral RNA analysis was conducted initially daily but limited to four treatment plants (Zurich, Chur, Geneva and Lugano) and focused exclusively on SARS-CoV-2. This scope expanded in late October 2022 to include RSV, IAV and IBV, and by July 2023, to all ten treatment plants but with a reduced frequency of 5 days per week. In total, microbial analyses were performed on 5,480 samples.

Sampling methodologies varied across the WWTPs: volume-proportional sampling was employed for those in Zurich, Bern, Solothurn, Chur, Schwyz, Geneva and Lugano. In contrast, the Basel WWTP used flow-proportional sampling and the Lausanne WWTP used time-proportional sampling (Supplementary Table 1). The Basel WWTP samples also composited multiple days when sampling occurred on weekends and holidays.

During the 24-h sampling period, samples were stored at 4 °C in auto-samplers. Post-collection, samples destined for chemical analysis were transferred to muffled 100-ml glass bottles and stored at −20 °C until analysis. Samples for viral RNA analysis were processed following methodologies outlined in previous studies^39–41^.

### Wastewater sample preparation for small molecule analysis

The wastewater samples were thawed and 1 ml of sample was centrifuged at room temperature at 280*g* for 10 min using a Centrifuge 5427 R (Eppendorf). Following centrifugation, 600 µl of the supernatant was transferred to a new glass vial. This supernatant was then spiked with 10 µl of an ethanol-based isotope-labelled internal standard (ISTD) mix containing the 15 structurally identical ISTDs, reaching a final concentration of 1,000 ng l^−1^, except for Paracetamol-D4 and Paraxanthine-D6, which had final concentrations of 10 µg l^−1^ (Supplementary Information).

A 12-point calibration in Evian mineral water, ranging from 10 ng l^−1^ to 40 µg l^−1^, was prepared from four ethanol-based working standard solutions. For acetaminophen, this range was extended with four additional calibration points, reaching up to 500 µg l^−1^.

For each measurement batch, three independently prepared samples were analysed to determine the precision of the analytical method. Additionally, we spiked aliquots of a specific wastewater sample with the target analytes at seven different concentration levels, ranging from 50 ng l^−1^ to 10 µg l^−1^, to assess analyte recoveries in the sample matrix (Supplementary Information).

### LC-HRMS measurements

The wastewater samples were analysed for the target substances using a large volume direct injection into a reversed-phase liquid chromatography system coupled to a high-resolution mass spectrometer (LC-HRMS). The chromatographic system comprised a PAL auto-sampler (CTC Analytics) and a Dionex UltiMate3000 RS pump (Thermo Fisher Scientific). A 100 µl sample was injected onto a reversed-phase C18 column (Atlantis T3 3 µm, 3.0 × 150 mm; Waters). Chromatographic separation was conducted at a flow rate of 300 µl min^−1^ using a mobile phase gradient, starting with 100% eluent A (water with 0.1% v/v formic acid). After 1.5 min, eluent B (methanol with 0.1% v/v formic acid) was ramped up to 95% over 17 min, held for 10 min and then returned to the initial conditions, followed by a 4-min re-equilibration phase.

The samples were analysed on a hybrid quadrupole-orbitrap high-resolution mass spectrometer (Orbitrap ExplorisTM 240 from Thermo Fisher Scientific) operating in positive ionization mode (ESI, 3.5 kV). Full scans were recorded at a resolution of 120,000 (at *m*/*z* 200), followed by five data-dependent MS/MS (fragment ion spectrum) experiments at a resolution of 30,000 (at *m*/*z* 200). Fragmentation of target ions was based on an inclusion mass list and was performed using higher-energy collision-induced dissociation with stepped collision energies of 15%, 45% and 90%.

The HRMS data were analysed using TraceFinder 5.1 (Thermo Fisher). Target analytes were quantified from the extracted ion chromatogram of the MS full-scan on the basis of the area ratio of the reference standard to the corresponding ISTD of the analyte. Detailed information on the LC-HRMS (liquid chromatography–high-resolution mass spectrometry) settings and measurement quality control are provided in the Supplementary Information.

### Population-normalized wastewater daily population loads

The concentrations measured in the wastewater samples (${c}_{\mathrm{sample}}$, chemical analytes in mg l^−1^, viral RNA in gc L^−1^) were multiplied by the daily wastewater volume (${\mathrm{Flow}}_{\mathrm{WWTP}}$, m^3^ d^−1^). This product was then divided by the estimated population (census) in the catchment area (${\mathrm{Pop}}_{\mathrm{WWTP}}$, number of people), resulting in population-normalized wastewater loads (DPL, mg d^−1^ per 1,000 people for chemical markers and gc d^−1^ per 1,000 people for viral loads), as described in equation (1).

1 $${\mathrm{DPL}}=\,\frac{{c}_{\mathrm{sample}}\times {\mathrm{Flow}}_{\mathrm{WWTP}}\,}{\mathrm{{Pop}}_{\mathrm{WWTP}}}\times \mathrm{1,000}.$$

For Lugano WWTP, three data points were excluded owing to unusually high loads, probably from sewer disposal or industrial sources.

Alternative normalization approaches using the population proxies candesartan (an antihypertensive) and paraxanthine (a caffeine metabolite) have been explored in the Supplementary Information.

### Swiss Sentinella practice-based research network

Data on weekly consultations for influenza-like illnesses and acute respiratory infection symptoms across Switzerland, along with associated laboratory testing results, were obtained from the Swiss Sentinella Network. The data are managed by the Swiss Federal Office of Public Health and are publicly available^26^. The Sentinella network comprises a volunteer cohort of 160–180 general practitioners, internists and paediatricians who contribute data on the incidence of circulating respiratory virus infections across Switzerland^42,43^.

Participating practitioners submit weekly reports on initial consultations and send nasopharyngeal swabs from cases to the National Reference Centre for Influenza for diagnostic analysis. The samples are tested for several respiratory viruses, including SARS-CoV-2, RSV, IAV and IBV, as well as other respiratory viruses such as adenovirus, rhinovirus, bocavirus, additional coronaviruses (229E, HKU1, NL63 and OC43), metapneumovirus and parainfluenza viruses (types 1–4).

We derived population-normalized estimates of specific respiratory infection cases by multiplying the test positivity rate by the population-normalized consultation incidence. The population-normalized consultation numbers (cases per 100,000 inhabitants) were provided by the FOPH and based on the relative population coverage of the participating doctors within the Sentinella system.

### SARS-CoV-2 cases

SARS-CoV-2 case data were sourced from the Swiss mandatory reporting system for infectious diseases, overseen by the Federal Office of Public Health (FOPH). Catchment-level data for Zurich, Chur, Geneva, and Lugano based on postal codes corresponding to the catchment areas of the respective WWTPs. This data are publicly accessible for the duration of the study period.

### Wastewater respiratory virus and pharmaceutical load modelling

The wastewater pharmaceutical loads (${\mathrm{DL}}_{t}$) were modelled as a function of the viral gene copies of the key respiratory viruses SARS-CoV-2, RSV, IAV and IBV. The equation used is as follows:

2 $$\begin{array}{l}{\mathrm{DL}}_{t}={\beta }_{0,{\mathrm{WWTP}}}+{\beta }_{\mathrm{SARS}\text{-}\mathrm{CoV}\text{-2}}\times {\mathrm{VL}}_{\mathrm{SARS}\text{-}\mathrm{CoV}\text{-2},t}\,+{\beta }_{\mathrm{RSV}}\times {\mathrm{VL}}_{\mathrm{RSV},t}\\\qquad\,+\,{\beta }_{\mathrm{IAV}}\times {\mathrm{VL}}_{\mathrm{IAV},t}+{\beta }_{\mathrm{IBV}}\times {\mathrm{VL}}_{\mathrm{IBV},t}+{\epsilon }_{t}.\end{array}$$

In this model:

- ${\beta }_{0,{\mathrm{WWTP}}}$ describes the WWTP and substance-specific baseline load. This represents the mean pharmaceutical load from periods of low respiratory virus activity (June 2021 and July to August 2023), as determined from wastewater virus and sentinel surveillance data (Fig. 2).
- ${\beta }_{\mathrm{SARS}-{\mathrm{CoV}-2}}$, ${\beta }_{\mathrm{RSV}}$, $\,{\beta }_{\mathrm{IAV}}$ and $\,{\beta }_{\mathrm{IBV}}$ are the coefficients for the viral loads of SARS-CoV-2, RSV, IAV and IBV, respectively.
- ${\mathrm{VL}}_{\mathrm{SARS}-{\mathrm{CoV}-2},{t}}$, ${\mathrm{VL}}_{\mathrm{RSV},t}$,$\,{\mathrm{VL}}_{\mathrm{IAV},t}$ and $\,{\mathrm{VL}}_{\mathrm{IBV},t}$ denote the 7-day median viral gene copy loads for each respective virus at day $t$. For RSV, IAV and IBV, this refers to copies of the Matrix protein gene (M), and for SARS-CoV-2, it refers to the Nucleoprotein gene locus 2 (N2), as detailed in ref. ^43^.
- The error term ${\epsilon }_{t}\, \sim {N}(0,{\sigma }^{2})$ represents the unexplained variability in the model. It follows a normal distribution with a mean of 0 and a constant variance ${\sigma }^{2}$. This term accounts for random fluctuations and factors not captured by the viral load variables.

We performed OLS regression to determine estimates of the coefficients (${\beta }_{1}$, ${\beta }_{2}$, $\,{\beta }_{3}$ and $\,{\beta }_{4}$), which quantify the contribution of virus-specific gene copy loads to the baseline-subtracted pharmaceutical load. A single OLS model was applied across all treatment plants, assuming a consistent relationship across locations. Diagnostic information for the OLS regressions is provided in the Supplementary Information.

All analyses were conducted in Python 3.10 using the statsmodels 0.14.0 package for OLS regression. Access to code and computational environments used for evaluation is provided in the data and code availability statements.

### Reporting summary

Further information on research design is available in the Nature Portfolio Reporting Summary linked to this article.

## Supplementary information

Supplementary Information Supplementary study characteristics and results.

Reporting Summary

## Data Availability

The dataset, along with the corresponding analysis and visualization scripts, is available via Eawag’s Research Data Institutional Collection at 10.25678/000D6F (ref. ^44^).
