# Supplementary material for: Insights into respiratory illness at the population level through parallel analysis of pharmaceutical and viral markers in wastewater
Source: Nat Water. 2025 May 14;3(5):580–9. doi: 10.1038/s44221-025-00437-4 (PMC12098119; doi:10.1038/s44221-025-00437-4)
Supplement: Supplementary file 1 — Supplementary study characteristics and results. [file 44221_2025_437_MOESM1_ESM.pdf]

nature water

Supplementary information https://doi.org/10.1038/s44221-025-00437-4

Insights into respiratory illness at the population level through parallel analysis of pharmaceutical and viral markers in wastewater

In the format provided by the authors and unedited

Contents

SI 1 Materials and methods 2
- SI 1.1 Wastewater treatment plant (WWTP) characteristics and wastewater sampling procedure 2
- SI 1.2 Exploring normalization approaches using chemical proxies to account for population dynamics 7
- SI 1.3 Chemicals and solutions 11
- SI 1.4 Chemical marker characteristics 12
- SI 1.5 Analytical instrumentation and method 13
- SI 1.6 Quantification method and quality control 13
  - SI 1.6.1 Quantification 13
  - SI 1.6.2 Matrix effects 13
  - SI 1.6.3 Limit of quantification 14
  - SI 1.6.4 Accuracy 14
  - SI 1.6.5 Precision 14

SI 2 Results and discussion 15
- SI 2.1 LCMS quantification 15
- SI 2.2 Spatiotemporal patterns of chemical and viral loads in wastewater 19
  - SI 2.2.1 Spatiotemporal pattern of baseline-subtracted loads for pharmaceuticals 23
  - SI 2.2.2 Influenza subtype clinical surveillance data 24
  - SI 2.2.3 Temporal trends of cases from mandatory reporting system and sentinel consultations 25
  - SI 2.2.4 Distribution of respiratory viruses from sentinel clinical testing 26
- SI 2.3 Correlation analysis of wastewater markers 26
  - SI 2.3.1 Pearson correlations chemical and viral loads (location-specific) 26
  - SI 2.3.2 Pearson correlations chemical and viral loads (location independent) 27
  - SI 2.3.3 Pearson correlations baseline-subtracted chemical and viral loads (location independent) 27
  - SI 2.3.4 Scatter plot of viral and pharmaceutical loads (location independent) 28
- SI 2.4 Modeling of relationship between pharmaceutical and viral loads in wastewater 32
  - SI 2.4.1 Ordinary least squares analyses with viral loads as predicting variables 33
  - SI 2.4.2 Ordinary least squares analyses with clinical cases as predicting variables 45

SI 3 In-sample stability of chemical markers 50
- SI 3.1 Sample preparation and analytical procedure 50
- SI 3.2 Data analysis 50
- SI 3.3 Stability results 50

SI 4 Licensed pharmaceutical products in Switzerland containing target chemicals 56

SI 5 References 57

SI 1 Materials and methods

SI 1.1 Wastewater treatment plant (WWTP) characteristics and wastewater sampling procedure

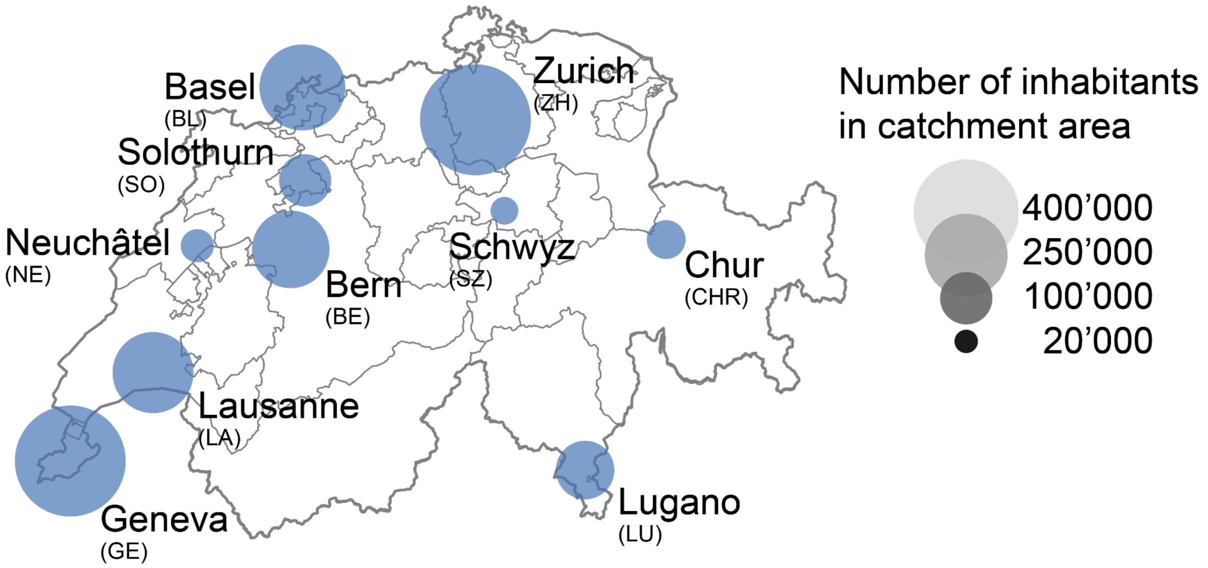

Figure SI 1: Geographic distribution of wastewater treatment plants (WWTPs) included in the study. The location of each WWTP is indicated by the center of a dot, with the dot size proportional to the catchment population served by the respective plant. The selection encompasses WWTPs from the different linguistic regions, including German-speaking (ZH, BL, BE, SO, CHR, SZ), French-speaking (GE, LA, NE), and Italian-speaking (LU) areas, representing both rural and urban environments.

Table SI 1: Characteristics of wastewater treatment plants (WWTPs) and details of the wastewater sampling procedures, as reported by WWTP operators.
(PE**:** Population Equivalent**,** n: count)

Location	Zurich	Basel	Bern	Solothurn	Chur	Schwyz	Geneva	Lausanne	Neuchâtel	Lugano
**Name of the Wastewater Treatment Plant**	ARA Werdhölzli	ProRheno AG	ara region bern ag	ARA Emmenspitz	ARA Chur	ARA-Schwyz	STEP Aïre	STEP EPURA	STEP Neuchâtel	IDA-Bioggio
**Date of Processing**	31.05.2023	03.01.2023	15.02.2023	26.1.2023	27.12.2023	26.01.2023	10.02.2023	16.05.2023	10.7.2024	10.02.2023
**Residential population [p]**	471275	273075	224557	95793	53123	31164	454433	247824	40697	123240
**Date of Census**	2022	2022	31.12.2021	1.1.2022	-	1.Januar 2022	31.12.2022	31.12.2022	1.1.2024	31.12.2021
**Design Capacity [PE]**	670000	477000	500000	125000	110000	70000	600000	350000	65000	186700
**Current Total Load [PE]**	693914	270000	400000	105000	75000	52000	696000	276612	47500	124416
**Loads from Industrial Sources [PE]**		220000	103000	-	10000	-	-	-	-	11000
**Most reliable number residential population**	Census	Census	Census	Census	Census	Census	Census	Census	-	Census
**Weekday population fluctuations**	Yes	No	Yes	No	No	No	Yes	-	No	Yes
**Increase during workdays**	Increase in people and discharge	-	Increase in people and discharge	-	-	-	Increase in people	-	-	Increase in people and discharge
**Number of hospitals in catchment**	14	16	10	>1	>1	1	7	6	2	>1
**Hospital beds of all the hospitals listed above [n]**	3665	2331	Last survey in 2010: 2216 beds	-	-	121	Unknown	1732	-	-
**pump stations in catchment [n]**	38	3	47	14	5	14	33	2	15	25
**Exfiltration**	No	Yes	-	Yes	-	Yes	No	Yes	No	Yes
**Max. flow distance [km]**	15	13	-	20	20	11	26.31	17	13	15
**Average hydraulic sewer residence time [h]**	1	1.5	-	3	5	1.2	3	0.66	1.5	3
**Location inflow measurement**	After sand trap	After sand trap	After the sand trap	In effluent	After screen	Before pumping station.	Inflow	-	Before screen	After screen (4 inluents)
**Inflow measurement method**	Venturi	Radar (Endress+Hauser FMR60)	MID	Venturi	Venturi	Venturi	Water level and velocity	-	Water level and velocity	Venturi, MID
**Flow meter calibration interval [months]**	0	0	12	6	12	0	0	-	0	12
**Flow measurement accuracy estimation [%]**	-	1	± 2 %	-	5	-	5	-	10	2
**Flow measuremnt accuracy manufacturer [%]**	10%	1%	-0.88%	5	5	5-10	unknown	-	5	2
**Lifting pump station operation**	Continuously	Continuously	None	Continuously	None	Continuously	None	None	-	None
**Wastewater volume on dry weather day [m^3d^{-1}]**	143655	173000	63303	52000	13000	12000	132732	-	10500	35000
**Minimum flow on dry weather day [Ls^{-1}]**	750	500	265	500	40	83	700	-	75	250
**Maximum flow on dry weather days [Ls^{-1}]**	3000	1000	1165	1000	250	242	2300	-	200	600
**Maximum flow on rainy weather day [Ls^{-1}]**	6000	2000	3000	4500	550	1500	12000	-	1000	2500
**Maximum threshold volume considered for evaluation [m^2 d^{-1}] (*empirically determined)**	420000	120000	200000	130000	40000	70000	350000	150000	35000	150000

Sampling location	After the fine screen	After sand trap (until July 2022), then after fine screen	After fine screen	After sand trap	After fine screen	After fine screen	Before coarse screen.	-	Between coarse screen and sand trap	Before coarse screen
**Manufacturer and type of sampling device**	WaterSam WS316 MS3	Bühler BU4011	Bühler 4010	Gerber WS 312	Gerber	Endress&Hauser CSF48-MD50/0	Endress&Hauser CSF48	Teledyne ISCO	Hach Bühler 4011	Endress&Hauser CSF48
**Temperature in sampling device [°C]**	4°C or cooler	4°C or cooler	4°C or cooler	4°C or cooler	4°C or cooler	4°C or cooler	4°C or cooler	4°C or cooler	4°C or cooler	4°C or cooler
**Time until sample freezing [h]**	7	1	0.75	1	2	0	0.5	2	8	0.5
**Bottles in sampler [n]**	4	4	1	4	4	1	1	1	4	4
**Total volume in sampler [L]**	72	40	30	48	25	30	20	20	10	48
**Composite sample duration [h]**	24	24 (48-72 on weekend/holidays)	24	24	24	24	24	24	24	24
**Material of sampling bottles**	PE	Plastic	PE-LD	Plastic	-	Plastic	PEHD	Plastic	Plastic	PEHD
**Sampling mode**	Volume-proportional	Flow-proportional	Volume-proportional	Volume-proportional	Volume-proportional	Volume-proportional	Volume-proportional	Time-proportional	Volume-proportional	Volume-proportional
**Sampling interval**	800 m^3	10 min	-	15 m^3	90 m^3	60 m^3	1460 m^3	5 min	175 m^3	500 m^3
**Storage temperature for collected sample at WWTP [°C]**	-20	-20	-20	-20	-20	-20	-20	-20	-20	-20

Maximum threshold flow volumes were determined based on dates when pharmaceutical loads were systematically under- or overestimated due to high inflows associated with heavy rainfall events. These events likely led to the discharge of untreated wastewater, resulting in the underestimation of pharmaceutical loads and introducing uncertainty in both sampling and flow measurement at the treatment plant. Consequently, for wastewater samples collected on days when the inflow volume at the treatment plant exceeded the maximum threshold volume specified in Table SI 1, the corresponding pharmaceutical load data were excluded from analysis due to the associated uncertainties. In contrast, viral gene copy load data from these dates were still considered reliable, as they represent 7-day centered median values, which reduce the impact of such outliers.

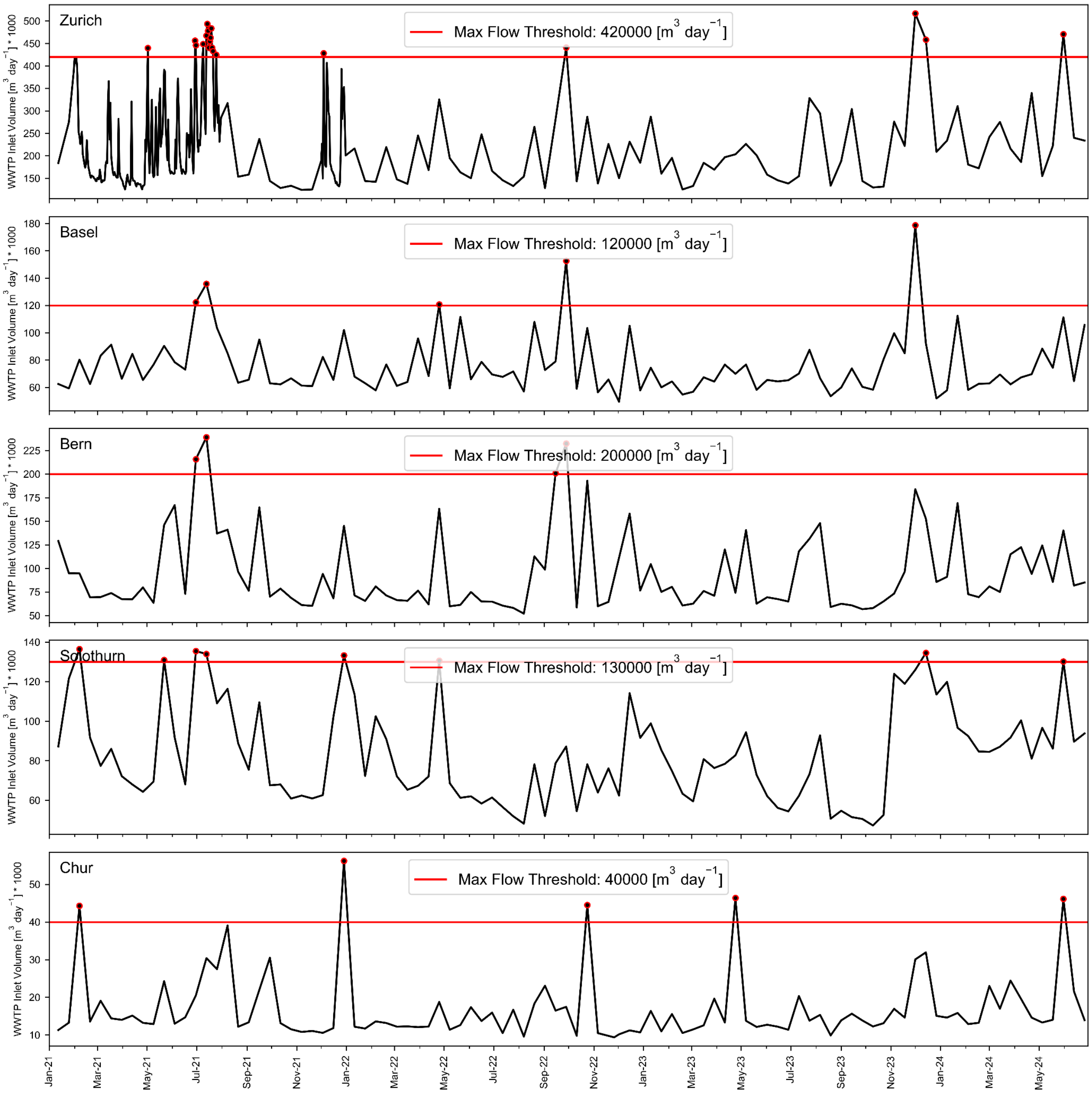

Figure SI 2: Daily wastewater inlet volumes at wastewater treatment plants (WWTPs). Black lines represent the daily inlet volumes on the days samples were collected for chemical analysis. The red line denotes the empirically determined, location-specific maximum volume threshold, above which daily load estimates are considered unreliable due to the discharge of untreated wastewater and potential irregularities in flow measurement and sampling. Red dots indicate the days when inlet volumes exceeded this threshold; data from these days were excluded from further analysis in the study.

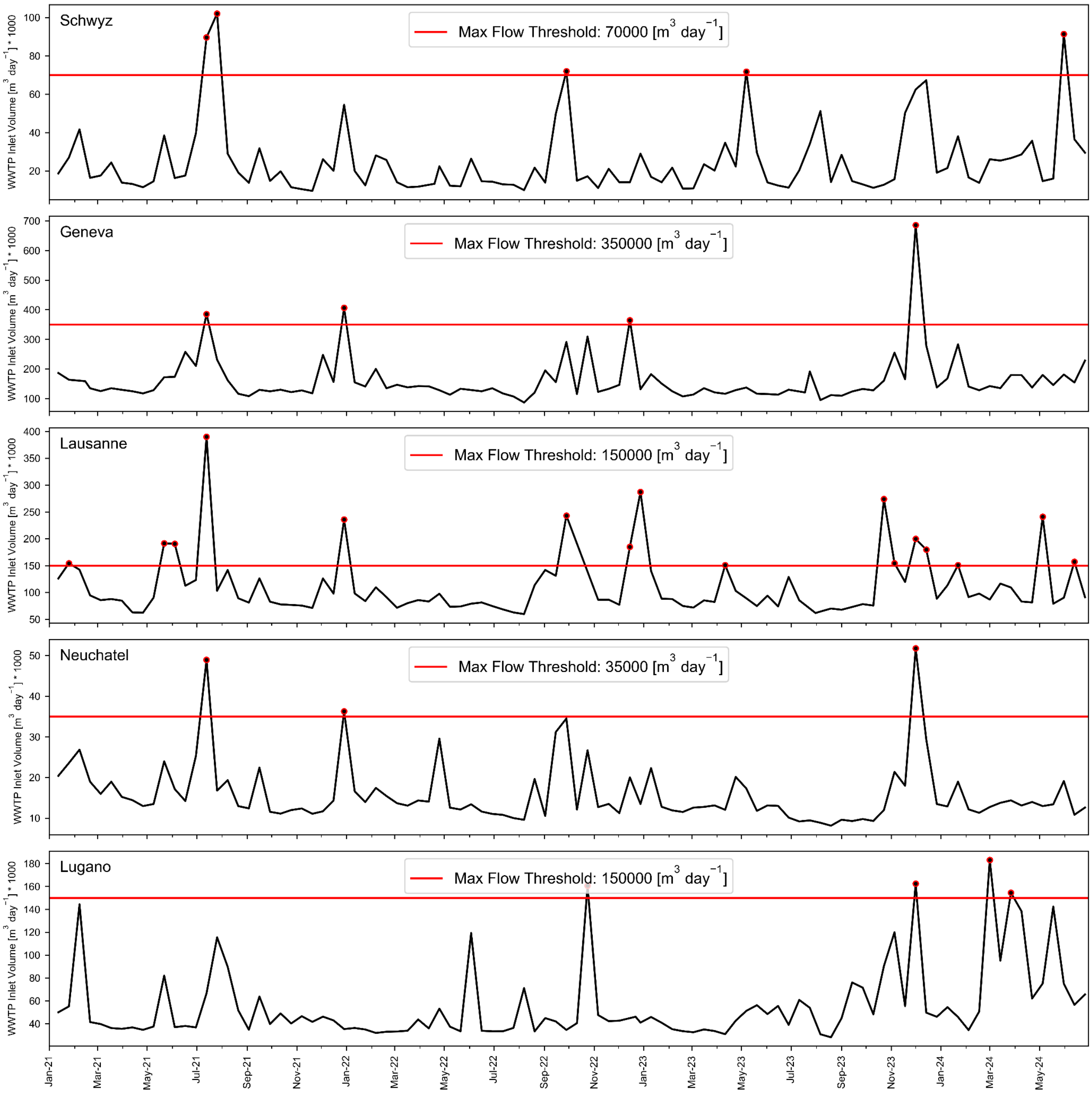

Figure SI 3: Daily wastewater inlet volumes at wastewater treatment plants (WWTPs). Black lines represent the daily inlet volumes on the days samples were collected for chemical analysis. The red line denotes the empirically determined, location-specific maximum volume threshold, above which daily load estimates are considered unreliable due to the discharge of untreated wastewater and potential irregularities in flow measurement and sampling. Red dots indicate the days when inlet volumes exceeded this threshold; data from these days were excluded from further analysis in the study.

SI 1.2 Exploring normalization approaches using chemical proxies to account for population dynamics

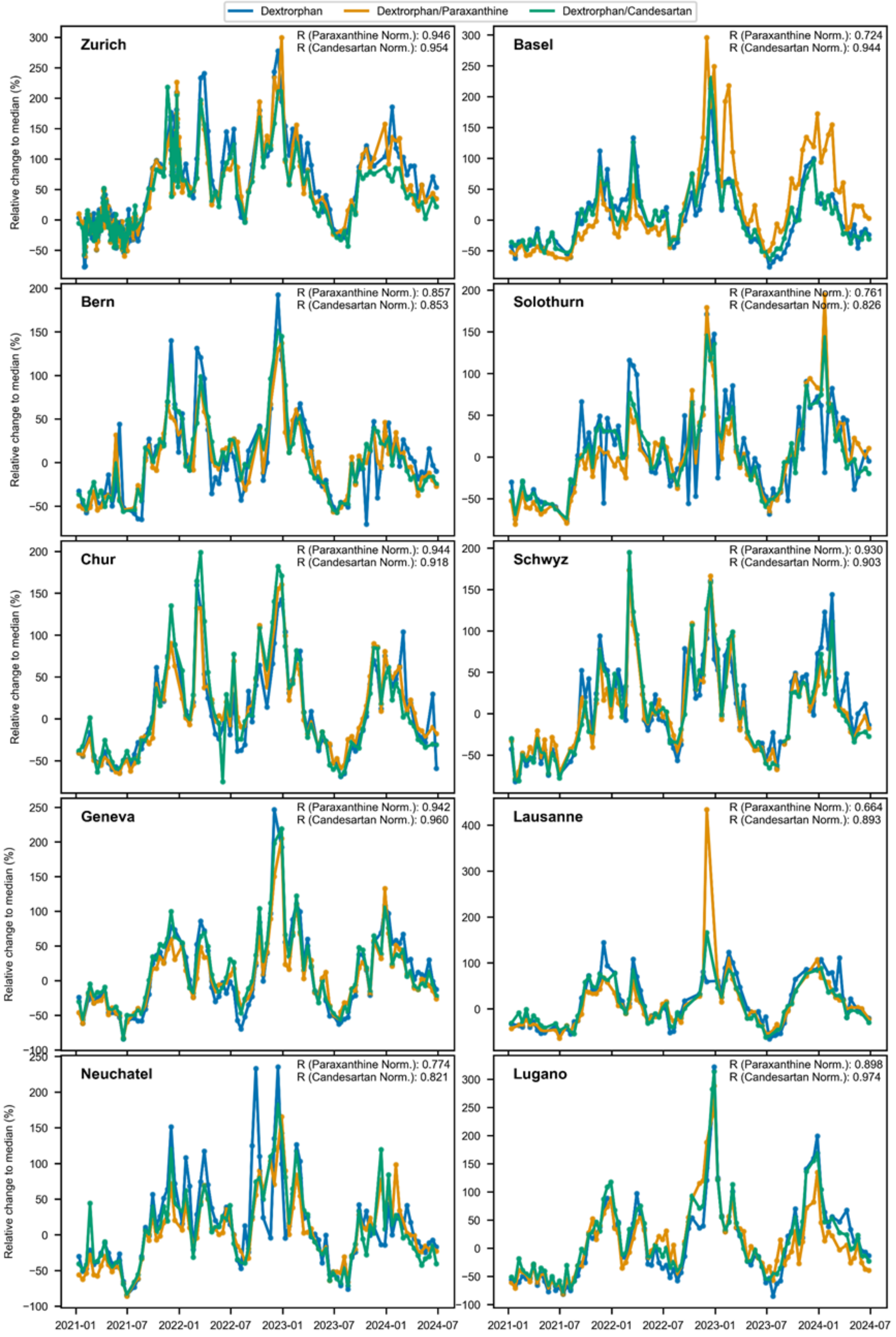

Figure SI 4: Comparison of the impact of different normalization approaches on relative dextrorphan trends. The Figure displays relative changes (%) for median dextrorphan loads normalized by census (blue, mg/d/1000p), dextrorphan normalized by paraxanthine (orange), and dextrorphan normalized by candesartan (green). Dextrorphan values are based on population-normalized loads [mg/d/1000p] using a static population (census), as described in the manuscript. Pearson correlation coefficients (R) were calculated between dextrorphan and its population proxy-normalized values (dextrorphan/paraxanthine and dextrorphan/candesartan) for each location.

The strong correlations between dextrorphan loads normalized using a static population and those normalized with candesartan (an antihypertensive) and paraxanthine (a caffeine metabolite) suggest that population fluctuations are not the primary drivers of the observed wastewater patterns.
Therefore, we used census to calculate population-normalized loads for the analyses presented in the manuscript, as this approach facilitates comparisons across locations and with other studies. The use of population proxies may vary geographically, making direct comparisons less reliable.
However, during extreme rain events, where uncertainties in sampling, flow measurements, and potential discharge of untreated wastewater may compromise data accuracy, census-based normalization may not be appropriate. In such cases, these values were excluded, as described in Figure SI 2 & Figure SI 3. Under these conditions, normalization using population proxies would provide a more robust alternative, as it mitigates these variability.
To visualize the theoretical effects of variations in flow and population on the observed temporal patterns of viral RNA markers, symptom-associated pharmaceutical markers such as dextrorphan, and population proxies such as candesartan (whose consumption is assumed to remain stable over time), we have simulated different synthetic scenarios (Figure SI 5).

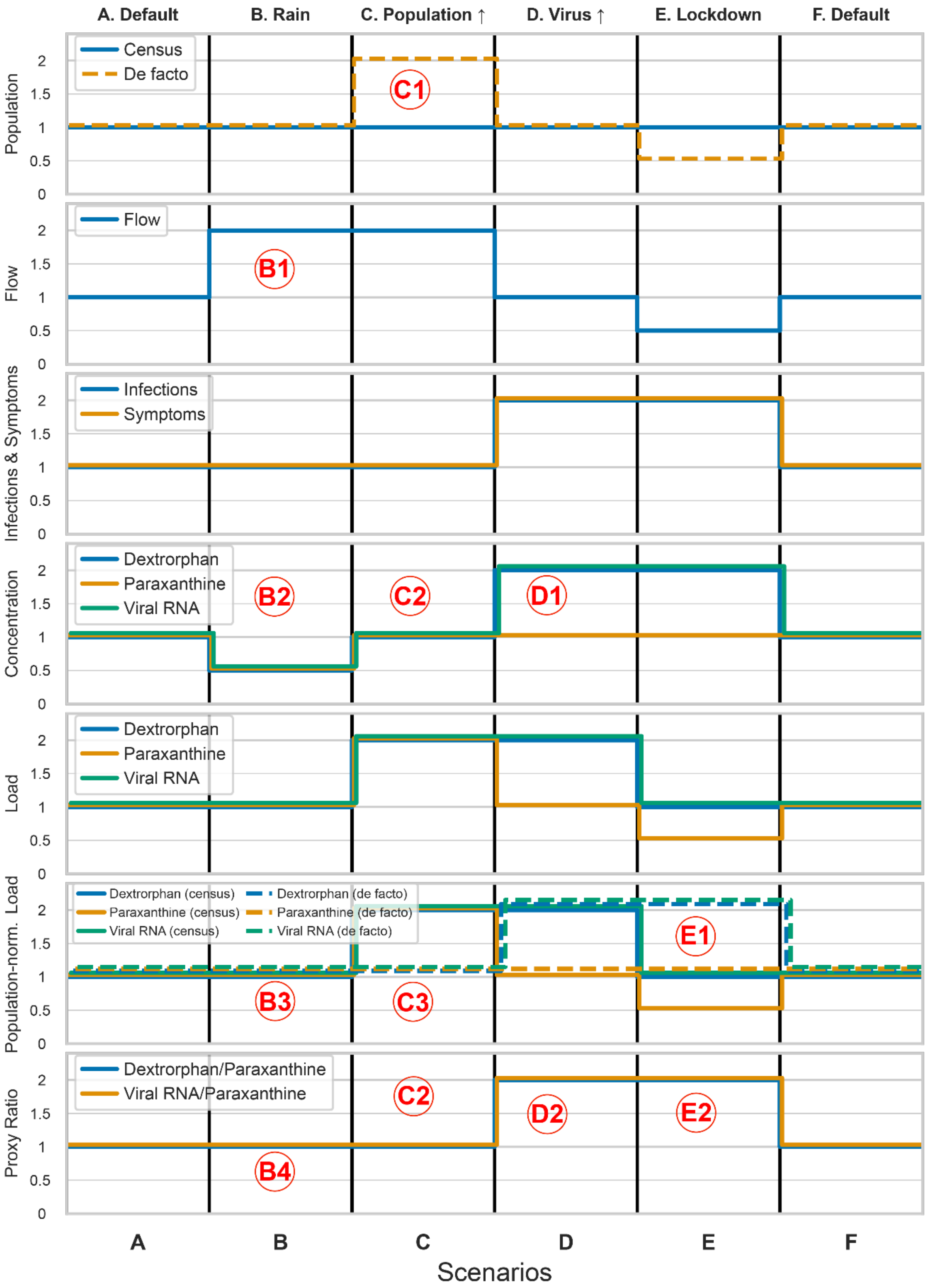

Figure SI 5: Synthetic scenarios illustrating the impact of variations in catchment population and flow on the concentrations, loads, and population-normalized ratios of an example symptom marker (dextrorphan, associated with coughing), viral RNA (indicative of respiratory viruses), and a population proxy (paraxanthine, a caffeine metabolite). The following scenarios are considered: **A & F** Default state: The de facto population matches the census population under dry weather conditions with standard daily flow. **B** Rain scenario: Increased flow (B1) leads to diluted concentrations of all markers (B2), while absolute and population-normalized loads remain unchanged since they are calculated as multiplication of concentration and flow (B3). The ratio between the target analytes (viral RNA or symptom marker) and the population proxy remains constant, as all concentrations decrease proportionally (B4). **C** Increased population: A temporary population surge (C1), for instance, due to a special event, results in higher flow and loads. However, the concentrations, and ratios with the population proxy remain

approximately unchanged (C2). The population-normalized loads using the de facto population remain stable (correct), while those based on the census population erroneously appear elevated due to the unaccounted population increase (C3). **D** Increased viral circulation (with no change of population size): A rise in infections leads to increased wastewater concentrations (and loads) of viral RNA and symptom markers (D1), while the population proxy (paraxanthine) remains unaffected, as its consumption is assumed independent of infections. Consequently, the ratio of viral RNA and the symptom marker to the population proxy increases (D2). **E** Lockdown scenario: In parallel to high infection levels, the de facto population in the catchment may decreases due to mobility restrictions. As a result, concentrations and loads of the population proxy (paraxanthine) decrease, accurately reflecting the reduced population. When normalizing with the de facto population, higher population-normalized loads of viral RNA and the symptom marker are observed. However, normalization using static census data fails to account for this population shift (E1). The ratio of the viral marker and symptom marker (dextrorphan) to the population marker (paraxanthine) increases, correctly reflecting the impact of the reduced population (E2).

SI 1.3 **Chemicals and solutions**

Organic solvents were of HPLC grade purity (> 98%) and supplied by Merck (ethanol) or Fisher Chemical (methanol and acetonitrile). Ultrapure water was obtained from a laboratory purification system (Arium Pro, Sartorius). Concentrated formic acid (>98%) was supplied by Merck.

Stock solutions of the individual analytes (STDs & ISTDs) were prepared at a concentration of 1 g/L in ethanol, methanol, or in acetonitrile (ACN) as described in Table SI 2. Individual stock solutions were combined into working mix solutions of 500, 60, 6, and 0.6 µg/L in EtOH. For acetaminophen, an additional working solution of 6 mg/L in ethanol was used for the high calibration range, extending up to 5000 µg/L. The isotope-labeled standard mix was prepared in ethanol at a concentration of 60 µg/L, with acetaminophen-D4 and paraxanthine-D6 included at a higher concentration of 600 µg/L. All solutions were stored at -20°C.

Table SI 2: Analytical reference standards (STD) and corresponding isotope-labelled standards (ISTD)

Substance	CAS-No	Molecular formula	Manufacturer	Solvent	Conc. [g/L]	Corresponding ISTD
Dextrorphan	125-73-5	C17H23N1O1	Lipomed AG	MeOH	1	Dextrorphan-D3
Codeine	76-57-3	C18H21N1O3	Lipomed AG	MeOH	1	Codeine-D6
Morphine	57-27-2	C17H19N1O3	Lipomed AG	MeOH	1	Morphine-D3
Tramadol	27203-92-5	C16H25N1O2	Sigma (*HCl)	EtOH	1	Tramadol-D6
Diclofenac	15307-86-5	C14H11N1O2Cl2	Sigma (*Na)	EtOH:H2O (1:1)	1	Diclofenac-D4
Naproxen	22204-53-1	C14H14O3	TRC-Canada	MeOH	1	Naproxen-D3
Paracetamol (Acetaminophen)	103-90-2	C8H9N1O2	Cerilliant	MeOH	1	Paracetamol-D4
Pheniramine	86-21-5	C16H20N2	Sigma-Aldrich (*maleate)	EtOH	1	Pheniramine-D6
N-desmethylpheniramine	19428-44-5	C15H18N2	LGC (*maleate)	MeOH	1	Pheniramine-D6
Clarithromycin	81103-11-9	C38H69N1O13	TCI EUROPE	EtOH	1	Clarithromycin-D3
Metronidazole	443-48-1	C6H9N3O3	Sigma-Aldrich	EtOH	1	Metronidazol-D4
Sulfamethoxazole	723-46-6	C10H11N3O3S1	Sigma-Aldrich	EtOH	1	Sulfamethoxazol-D4
Trimethoprim	738-70-5	C14H18N4O3	Sigma-Aldrich	EtOH	1	Trimethoprim-D3
Candesartan	139481-59-7	C24H20N6O3	TRC-Canada	ACN	1	Candesartan-D5
Paraxanthine	611-59-6	C7H8N4O2	Cerilliant	MeOH	1	Paraxanthine-D6
Dextrorphan-D3	-	C17H20[2]H3N1O1	Lipomed AG	MeOH	1	-
Codeine-D6	1007844-34-9	C18H15[2]H6N1O3	Lipomed AG	MeOH	1	-
Morphine-D3	67293-88-3	C17H16[2]H3N1O3	Sigma-Aldrich	MeOH	1	-
Tramadol-D6	-	C16H19[2]H6N1O2	TRC-Canada (*HCl)	MeOH	1	-
Diclofenac-D4	153466-65-0	C14H7[2]H4N1O2Cl2	TRC-Canada	MeOH	1	-
Naproxen-D3		C14H11[2]H3O3	C/D/N Isotopes Inc.	ACN	1	-
Paracetamol-D4 (Acetaminophen-D4)	64315-36-2	C8H5[2]H4N1O2	TRC-Canada	EtOH	1	-
Lidocaine-D10	1189959-13-4	C14H12[2]H10N2O1	TRC-Canada (*HCl)	EtOH	1	-
Pheniramine-D6		C16H14[2]H6N2	TRC-Canada	MeOH	1	-
Clarithromycin-D3	959119-17-6	C38H66[2]H3N1O13	TRC-Canada	MeOH	0.5	-
Metronidazol-D4	1261392-47-5	C6H5[2]H4N3O3	TRC-Canada	EtOH	1	-
Sulfamethoxazol-D4	1020719-86-1	C10H7[2]H4N3O3S1	TRC-Canada	ACN	1	-
Trimethoprim-D3	1189923-38-3	C14H15[2]H3N4O3	TRC-Canada	MeOH	1	-
Candesartan-D5	1189650-58-5	C24H15[2]H5N6O3	TRC-Canada	MeOH	1	-
Paraxanthine-D6	117490-41-2	C7H2[2]H6N4O2	Cayman Chemical	MeOH	0.1	-

For the quantification of Pheniramine and N-desmethylpheniramine using LC-MS, the isotope-labeled internal standard was changed from Lidocaine-D10 to Pheniramine-D6 during the course of the project. Consequently, samples collected from April 11, 2023, onwards in Bern, Geneva, Lausanne, and Neuchâtel, and from July 11, 2023, onwards in Zurich, Basel, Solothurn, Chur, Schwyz, and Lugano were quantified using Pheniramine-D6. Samples collected before these dates were quantified using Lidocaine-D10. To account for potential deviations in absolute concentrations, relative recoveries were determined. Additionally, aliquots of spiked wastewater and mineral water (referred to as 'eternity samples') were quantified in each measurement batch to ensure consistency over time, as detailed in the long-term stability analysis (SI 3.3).

SI 1.4 **Chemical marker characteristics**

Opioid analog

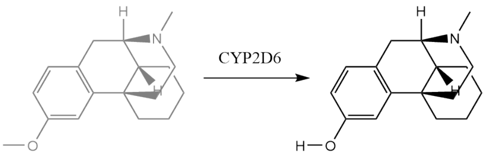

Dextromethorphan

Dextrorphan
(metabolite)

Opioids

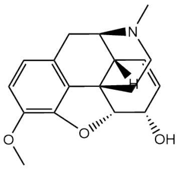

Codeine

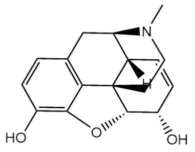

Morphine

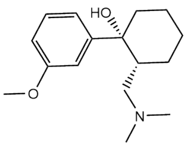

Tramadol

Non-opioid analgesic

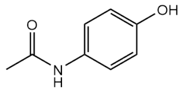

Acetaminophen / Paracetamol

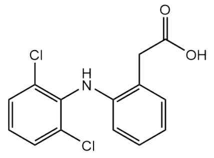

Diclofenac
(NSAID)

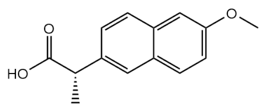

Naproxen
(NSAID)

Antihistamines

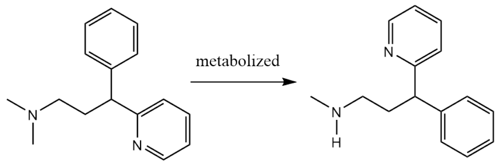

Pheniramine
(1st generation antihistamine)

N-desmethylpheniramine
(metabolite)

Antibiotics

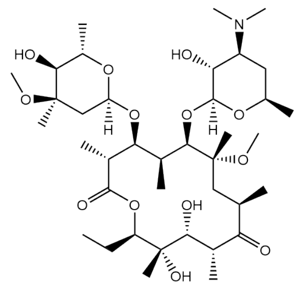

Clarithromycin
(macrolide)

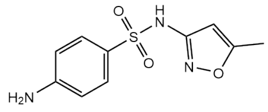

Sulfamethoxazole
(sulfonamide)

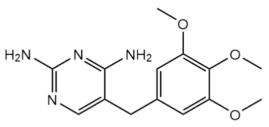

Trimethoprim
(antifolate)

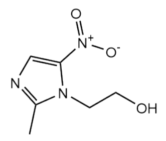

Metronidazole
(nitromidazole)

Controls

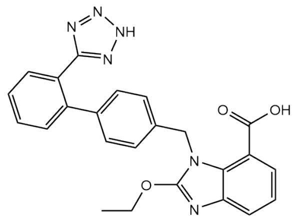

Candesartan
(angiotensin-receptor blocker)

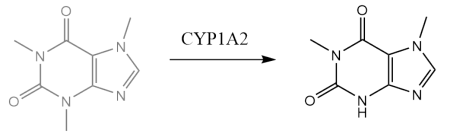

Caffeine

Paraxanthine
(metabolite)

Figure SI 6: Molecular structures of small molecule wastewater markers. Analytes highlighted in bold demonstrated a positive correlation with viral RNA gene copy loads of respiratory viruses, including SARS-CoV-2, Influenza A (IAV), and Respiratory Syncytial Virus (RSV) in wastewater samples.

SI 1.5 **Analytical instrumentation and method**

Table SI 3: Parameter settings used for HRMS/MS measurements with Exploris^{TM} 240 mass spectrometer

**Parameter**	**Value**
Ionization mode	positive
Spray voltage [kV]	3.5
Ion transfer tube temperature [°C]	320
Sheet gas flow (nitrogen) [AU]	40
Auxiliary gas flow (nitrogen) [AU]	10
Sweep gas flow [AU]	0
RF Lens [%]	60
Spectrum data type	Profile
Maximum injection time MS1 [ms]	100
Scan range MS1 [m/z]	100 - 1000
Resolution MS1	120000
Internal calibration	Yes (Run Start EASY-IC)
Data-dependent mode	Number of scans
Data-dependent trigger	Ions of mass list
Isolation window [m/z]	1
Resolution MS/MS	30000
Collision energy mode	Stepped
Collision energy type	Normalized
HCD collision energies [%]	15, 45, 90

AU: arbitrary units
ms: milliseconds
m/z: mass to charge ratio

SI 1.6 **Quantification method and quality control**

SI 1.6.1 **Quantification**

Target analytes were quantified based on the area ratio of the analyte and its selected isotope-labelled internal standard (ISTD) using Tracefinder 5.1 software. Structurally identical ISTDs were available for all analytes, except for the metabolie N-desmethylpheniramine. The target peaks were extracted from full scans with a mass tolerance of 5 ppm, and only peaks with at least five data points and a signal-to-noise ratio greater than 10 were considered for quantification. Extracted peaks were verified as target analytes by comparing the MS/MS spectra, retention time, and isotopic pattern to those of the corresponding reference standards and library spectra from MZCloud. The twelve-point calibration in Evian mineral water included the following concentration levels: 10, 25, 50, 100, 250, 500, 1000, 2500, 5000, 10000, 25000, 40000ng/L. For acetaminophen the calibration extended to 100, 250, 500 µg/L.
Linear calibration was applied to all substances, with regression weighting set to 1/x.
All quality control parameters of the LC-HRMS measurements that are described below were determined for each measurement batch independently.

SI 1.6.2 **Matrix effects**

The impact of matrix effects during analysis was evaluated by determining the absolute recoveries specific to each analyte. For target analytes with structurally identical ISTD, the absolute recovery is described as the ratio of the ISTD area in the matrix samples ($ISTD\ Area_{Matrix}$) to their average area in the calibration series ($Average\ ISTD\ Area_{Calibration}$), as described in equation SI 1:

$$Absolute\ Recovery = \frac{ISTD\ Area_{Matrix}}{Average\ ISTD\ Area_{Calibration}} \qquad (SI\ 1)$$

If a structurally identical ISTD was unavailable, the absolute recovery was calculated based on the difference of the target peak area in spiked samples ($STD\ Area_{spiked\ sample}$) and the area in the unspiked sample ($STD\ Area_{unspiked\ sample}$) and set into relation with the area in the respective calibration standard ($STD\ Area_{Calibration}$) (Equation SI 2).

$$Absolute\ recovery_{non-identical\ ISTD} = \frac{STD\ Area_{spiked\ sample} - STD\ Area_{unspiked\ sample}}{STD\ Area_{corresponding\ calibration\ standard}} \qquad (SI\ 2)$$

SI 1.6.3 **Limit of quantification**

The limit of quantification (LOQ) was determined based on the lowest concentration in the wastewater matrix, at which the quantification criteria (i. signal-to-noise ratio larger than 10 and ii. at least five data points) was met. If such an LOQ in the wastewater matrix was not determinable, the LOQ was determined based on the lowest quantified calibration level multiplied by the inverse of the absolute recovery (Equation SI 3).

$$LOQ_{matrix}\ [ngL^{-1}] = \frac{LOQ_{solvent}}{Absolute\ recovery} \qquad (SI\ 3)$$

Additionally, if the target analyte was detected in the matrix blanks (Evian mineral water spiked with ISTD and processed as the samples) an LOQ based on the equation below, with sd being the standard deviation, was calculated (Equation SI 4).

$$LOQ_{matrix\ blank}[ngL^{-1}] = Average\ C_{matrix\ blank} + 10 * sd(C_{matrix\ blanks}) \qquad (SI\ 4)$$

If multiple LOQ values were obtained the higher value was considered.

SI 1.6.4 **Accuracy**

The accuracy of the measurements was assessed by determining the relative recovery in wastewater samples that were deliberately spiked with specific concentrations as described in the equation below (Equation SI 5). In each measurement batch, a wastewater sample was spiked with the target analytes at the following concentrations; 50, 100, 250, 500, 1000, 2500, 5000, 10000ng/L.

$$Relative\ recovery\ [\%] = \frac{C_{spiked\ sample} - C_{unspiked\ sample}}{C_{spiked}} * 100 \qquad (SI\ 5)$$

Relative recoveries were only calculated if the concentration in the unspiked sample was lower than twice the spiked concentration. If the concentration in the unspiked sample was lower than twice the LOQ only recoveries from samples with a spiked concentration above four times the LOQ were reported. Recoveries were not considered if the total concentration in the spiked sample exceeded the highest calibration point concentration.

SI 1.6.5 **Precision**

Three aliquots of the same wastewater sample were prepared and analyzed separately in every measurement batch to assess the overall precision of the analytical method.

The precision was computed as the relative standard deviation of the three measurements (Equation SI 6).

$$Relative\ standard\ deviation\ [\%] = \frac{std\ (C_{Replicates})}{mean\ (C_{Replicates})} * 100 \qquad (SI\ 6)$$

SI 2 Results and discussion

SI 2.1 **LCMS quantification**

Table SI 4: Summary of LCMS Analytics Quality Control and Quantification. The data are presented as average values for individual measurement batches. Quality control parameters determined as described above. (m/z: Mass to charge ratio, RT: Retention time, LOQ: Limit of quantification, SD: Standard deviation, ND: Not determinable)

Substance	Measurement batch ID	m/z theoretical [M+H]+l	m/z delta [ppm]	RT measured [min]	SD RT [min]	LOQ [ng/L]	Sample conc. [ng/L]	Samples above LOQ [%]	Absolute recovery	Relative recovery [%]	Precision [%]	Number of samples
Acetaminophen	220613	152.0706	0.46	10.4	0.02	500	68428	99.3	0.51	ND	7.5	148
Acetaminophen	220623	152.0706	0.28	10.5	0.01	500	88752	100	0.6	ND	2.2	38
Acetaminophen	220704	152.0706	0.44	10.4	0.02	500	27054	97.2	0.63	120.2	9.7	143
Acetaminophen	220803	152.0706	0.20	10.5	0.01	500	28486	100	0.57	ND	2.7	81
Acetaminophen	221021	152.0706	0.06	10.2	0.02	500	23838	100	0.63	89.2	4.9	187
Acetaminophen	230109	152.0706	0.38	9.9	0.02	500	45478	99.4	0.57	125.6	3.2	181
Acetaminophen	230424	152.0706	0.20	10	0.01	500	40199	100	0.45	73.6	3.9	45
Acetaminophen	230711	152.0706	-0.17	10.3	0.01	500	64418	99	0.76	89.9	7.1	100
Acetaminophen	231106	152.0706	1.06	10.3	0.03	500	27980	100	0.51	ND	0.3	45
Acetaminophen	240131	152.0706	0.35	10.3	0.01	500	58097	100	0.57	ND	2.2	127
Acetaminophen	240701	152.0706	1.01	10.6	0.06	500	63367	99.3	0.62	115.4	1.9	143
Candesartan	220613	441.167	0.53	18.8	0.05	50	476	100	0.69	108.2	5.4	148
Candesartan	220623	441.167	0.47	18.8	0.04	50	521	97.4	0.71	105.3	4	38
Candesartan	220704	441.167	0.42	18.8	0.06	50	478	99.3	0.76	100	3.1	143
Candesartan	220803	441.167	0.08	18.7	0.03	50	345	100	0.73	100	1.8	81
Candesartan	221021	441.167	0.23	18.6	0.07	50	305	97.9	0.75	98.6	6.1	187
Candesartan	230109	441.167	0.50	18.3	0.04	50	483	100	0.65	100.7	1	181
Candesartan	230424	441.167	0.26	18.3	0.06	50	533	100	0.47	106.5	6.4	45
Candesartan	230711	441.167	0.04	18.5	0.04	50	514	100	0.6	108.7	3.2	100
Candesartan	231106	441.167	1.36	18.6	0.02	50	452	100	0.61	95.4	3.2	45
Candesartan	240131	441.167	0.06	18.7	0.01	50	486	100	0.73	102.3	4.5	127
Candesartan	240701	441.167	-0.15	18.8	0	50	476	100	0.67	96.2	3.9	143
Clarithromycin	220613	748.4842	0.43	16.4	0.07	60	159	92.6	0.82	104.2	7.7	148
Clarithromycin	220623	748.4842	0.51	16.4	0.05	60	182	89.5	0.8	102.7	5.7	38
Clarithromycin	220704	748.4842	0.30	16.5	0.08	60	181	76.2	0.86	99.9	1.4	143
Clarithromycin	220803	748.4842	0.20	16.6	0.05	60	157	96.3	0.75	96.6	0.9	81
Clarithromycin	221021	748.4842	0.04	16.3	0.08	60	155	94.1	0.73	102	10.8	187
Clarithromycin	230109	748.4842	0.28	16.2	0.07	60	168	89	0.72	101.4	7	181
Clarithromycin	230424	748.4842	0.44	16.2	0.09	60	248	95.6	0.77	98.6	6.7	45
Clarithromycin	230711	748.4842	0.02	16.6	0.08	60	203	94	0.81	102.4	1.6	100
Clarithromycin	231106	748.4842	1.67	16.3	0.02	60	155	88.9	0.49	102.1	2.5	45
Clarithromycin	240131	748.4842	0.22	16.6	0	60	188	92.9	0.61	98.9	2.1	127
Clarithromycin	240701	748.4842	0.03	16.7	0.09	60	232	95.8	0.59	89.3	4	143
Codeine	220613	300.1594	0.47	9	0.02	45	196	95.3	0.86	104.2	6.3	148
Codeine	220623	300.1594	0.28	9	0.02	45	202	97.4	0.96	108.3	7.5	38
Codeine	220704	300.1594	0.28	9	0.03	45	148	75.5	0.88	106	3.1	143
Codeine	220803	300.1594	0.08	9.1	0.02	45	105	89.9	0.93	107.9	ND	79
Codeine	221021	300.1594	0.18	8.9	0.02	45	102	79.3	0.97	100.3	ND	184
Codeine	230109	300.1594	0.63	8.7	0.02	45	160	92.3	0.75	103.3	6.5	181
Codeine	230424	300.1594	0.31	8.7	0.02	45	158	93.3	0.82	101.9	5.9	45
Codeine	230711	300.1594	0.28	9	0.02	45	182	93.9	0.85	107.9	1.6	99

Substance	Measurement batch ID	m/z theoretical [M+H]+I	m/z delta [ppm]	RT measured [min]	SD RT [min]	LOQ [ng/L]	Sample conc. [ng/L]	Samples above LOQ [%]	Absolute recovery	Relative recovery [%]	Precision [%]	Number of samples
Codeine	231106	300.1594	1.61	8.8	0.03	45	228	80.4	0.88	93.4	2.3	46
Codeine	240131	300.1594	0.82	8.8	0.02	45	194	89	0.92	102.6	5.9	127
Codeine	240701	300.1594	0.23	9	0.04	45	160	88.7	0.79	100	6.7	142
Dextrorphan	220613	258.1852	0.43	11.8	0.03	10	71	96.6	0.65	107.2	7.4	148
Dextrorphan	220623	258.1852	0.26	11.9	0.02	10	62	94.7	0.77	107.1	4.5	38
Dextrorphan	220704	258.1852	0.17	11.9	0.04	10	54	88.1	0.69	107.2	2	143
Dextrorphan	220803	258.1852	-0.11	12	0.03	10	51	96.2	0.69	105.9	ND	79
Dextrorphan	221021	258.1852	0.18	11.7	0.04	10	58	94	0.7	96.9	ND	184
Dextrorphan	230109	258.1852	0.50	11.5	0.04	10	77	99.4	0.63	103.3	5.1	181
Dextrorphan	230424	258.1852	0.32	11.5	0.04	10	98	100	0.72	98.4	4.5	45
Dextrorphan	230711	258.1852	0.17	12	0.04	10	81	99	0.72	108.6	1.3	99
Dextrorphan	231106	258.1852	1.51	11.6	0.03	10	46	95.7	0.8	92.7	1.5	46
Dextrorphan	240131	258.1852	0.91	11.7	0.04	10	63	100	0.72	97.6	2.2	127
Dextrorphan	240701	258.1852	0.66	12	0.07	10	64	99.3	0.68	90.2	1	142
Diclofenac	220613	296.024	0.14	20.6	0.01	100	1658	100	0.78	116	7.9	148
Diclofenac	220623	296.024	0.11	20.6	0.01	100	1236	97.4	0.8	107.3	3.1	38
Diclofenac	220704	296.024	0.09	20.6	0.02	100	1690	98.6	0.85	107.3	0.8	143
Diclofenac	220803	296.024	-0.28	20.6	0.01	100	1624	100	0.84	104.4	8.7	81
Diclofenac	221021	296.024	0.11	20.4	0.02	100	1334	100	0.79	106.2	4.6	187
Diclofenac	230109	296.024	0.38	20.2	0.01	100	1542	100	0.88	98.3	4.2	181
Diclofenac	230424	296.024	0.16	20.2	0.02	100	1760	100	0.69	100.4	1.5	45
Diclofenac	230711	296.024	-0.11	20.4	0.02	100	1359	100	0.88	101.2	1.1	100
Diclofenac	231106	296.024	1.41	20.5	0	100	1431	100	0.65	98.6	1.6	45
Diclofenac	240131	296.024	0.59	20.6	0	100	1252	100	0.92	101.9	2.6	127
Diclofenac	240701	296.024	-0.14	20.5	0	100	1266	100	0.89	91.7	2.1	143
Metronidazole	220613	172.0717	0.33	10.1	0.02	25	194	97.3	0.52	109.8	6	148
Metronidazole	220623	172.0717	0.27	10.2	0.02	25	194	97.4	0.61	114.5	3.7	38
Metronidazole	220704	172.0717	0.13	10.2	0.02	25	148	97.9	0.53	106.9	1.2	143
Metronidazole	220803	172.0717	-0.09	10.1	0.02	25	164	100	0.57	100.7	4.3	81
Metronidazole	221021	172.0717	0.07	9.9	0.02	25	145	100	0.55	105	2.5	187
Metronidazole	230109	172.0717	0.44	9.7	0.03	25	150	97.8	0.45	104.1	3.1	181
Metronidazole	230424	172.0717	0.27	9.7	0.02	25	161	100	0.47	102.9	1.2	45
Metronidazole	230711	172.0717	0.11	10.1	0.02	25	182	97	0.73	104.5	1.2	100
Metronidazole	231106	172.0717	1.27	10.1	0.02	25	150	100	0.59	100.6	1.7	45
Metronidazole	240131	172.0717	0.93	10.2	0.01	25	191	96.1	0.48	104	16.1	127
Metronidazole	240701	172.0717	1.24	10.3	0.04	25	172	97.2	0.54	105.2	6.2	143
Morphine	220613	286.1438	0.21	7.4	0.02	100	1046	98.6	1.04	85.8	15.7	148
Morphine	220623	286.1438	0.18	7.4	0.02	100	570	94.7	1.25	105.5	9.7	38
Morphine	220704	286.1438	-0.01	7.4	0.02	100	359	67.1	0.98	90.3	6.4	143
Morphine	220803	286.1438	-0.15	7.5	0.02	100	958	97.5	1.04	97.2	ND	79
Morphine	221021	286.1438	0.10	7.3	0.03	100	900	93.5	0.97	85.5	ND	184
Morphine	230109	286.1438	0.58	7.1	0.03	100	651	87.3	0.7	101.2	9.5	181
Morphine	230424	286.1438	0.26	7.2	0.01	100	613	91.1	0.84	93.6	10.6	45
Morphine	230711	286.1438	-0.01	7.5	0.03	100	685	92.9	0.87	100.9	8	99
Morphine	231106	286.1438	1.43	7.2	0.05	100	628	91.3	0.96	90.4	2.4	46
Morphine	240131	286.1438	0.72	7.3	0.02	100	690	90.6	0.98	81.3	4	127
Morphine	240701	286.1438	0.24	7.4	0.04	100	624	85.9	0.87	79.2	3.3	142

Substance	Measurement batch ID	m/z theoretical [M+H]+l	m/z delta [ppm]	RT measured [min]	SD RT [min]	LOQ [ng/L]	Sample conc. [ng/L]	Samples above LOQ [%]	Absolute recovery	Relative recovery [%]	Precision [%]	Number of samples
N-desmethylpheniramine	220613	227.1543	0.40	10.5	0.17	20	42	42.6	0.79	98.2	10.1	148
N-desmethylpheniramine	220623	227.1543	0.15	10.8	0	20	39	42.1	0.97	108.2	9.8	38
N-desmethylpheniramine	220704	227.1543	0.18	10.8	0	20	41	32.9	0.9	107.8	3.7	143
N-desmethylpheniramine	220803	227.1543	-0.03	10.5	0.04	20	28	34.6	0.79	98	3.4	81
N-desmethylpheniramine	221021	227.1543	0.35	10.2	0.04	20	46	36.9	0.82	100.2	ND	187
N-desmethylpheniramine	230109	227.1543	0.50	10.2	0.02	20	84	15.5	0.7	102.5	ND	181
N-desmethylpheniramine	230424	227.1543	0.41	10.2	0.02	20	53	82.2	0.89	101.7	11.6	45
N-desmethylpheniramine	230711	227.1543	-0.08	10.5	0.04	20	39	58	0.95	99.9	ND	100
N-desmethylpheniramine	231106	227.1543	1.46	10.1	0.04	20	41	31.1	0.79	88.1	20.2	45
N-desmethylpheniramine	240131	227.1543	1.13	10.5	0.02	20	53	66.9	0.86	116.5	4.8	127
N-desmethylpheniramine	240701	227.1543	1.07	10.8	0.11	20	42	53.1	1.11	103.3	4.4	143
Naproxen	220613	231.1016	0.29	19.2	0.01	300	2038	99.3	0.51	119.8	6	148
Naproxen	220623	231.1016	0.23	19.2	0.01	300	1502	97.4	0.62	107.8	5.7	38
Naproxen	220704	231.1016	0.20	19.2	0.01	300	1577	97.2	0.66	109	4.1	143
Naproxen	220803	231.1016	-0.15	19.2	0.01	300	1763	100	0.63	111.5	4.8	81
Naproxen	221021	231.1016	0.27	19	0.01	300	1539	98.9	0.58	93.2	7.7	187
Naproxen	230109	231.1016	0.59	18.8	0.01	300	1828	98.9	0.69	99.6	5.5	181
Naproxen	230424	231.1016	0.51	18.8	0.01	300	1857	100	0.46	106.2	3.5	45
Naproxen	230711	231.1016	0.25	19.1	0.02	300	1901	99	0.73	106.2	2.2	100
Naproxen	231106	231.1016	1.52	19	0.01	300	1527	100	0.5	88.9	3.5	45
Naproxen	240131	231.1016	1.22	19	0.01	300	1809	97.6	0.77	100.5	3	127
Naproxen	240701	231.1016	1.36	19.3	0.07	300	1714	98.6	0.81	88.1	3.2	143
Paraxanthine	220613	181.072	0.09	11.2	0.02	500	26744	96.6	0.64	ND	8.1	148
Paraxanthine	220623	181.072	-0.08	11.2	0.01	500	24303	100	0.72	ND	3	38
Paraxanthine	220704	181.072	-0.03	11.2	0.01	500	17694	86	0.71	107.9	5	143
Paraxanthine	220803	181.072	-0.28	11.3	0.01	500	25476	100	0.74	ND	1.2	81
Paraxanthine	221021	181.072	-0.20	11	0.01	500	21814	100	0.79	100.4	2.6	187
Paraxanthine	230109	181.072	0.13	10.8	0.01	500	20455	93.9	0.57	91	2.6	181
Paraxanthine	230424	181.072	0.09	10.8	0.01	500	20486	88.9	0.54	97.4	3.2	45
Paraxanthine	230711	181.072	-0.26	11.1	0.01	500	24761	100	0.66	112.7	8.4	100
Paraxanthine	231106	181.072	0.86	11.1	0.03	500	18790	100	0.61	ND	1	45
Paraxanthine	240131	181.072	0.58	11.2	0.02	500	22208	100	0.78	ND	2.3	127
Paraxanthine	240701	181.072	0.43	11.2	0.03	500	20914	100	0.77	ND	1.2	143
Pheniramine	220613	241.1699	0.08	10.6	0.06	20	52	64.9	0.64	80.8	4.1	148
Pheniramine	220623	241.1699	-0.14	10.6	0.05	20	61	50	0.78	90.6	2.6	38
Pheniramine	220704	241.1699	0.08	10.7	0.06	20	57	41.3	0.81	97.9	6.7	143
Pheniramine	220803	241.1699	-0.22	10.7	0.05	20	39	84	0.8	101.7	10.6	81
Pheniramine	221021	241.1699	0.00	10.5	0.05	20	57	49.2	0.77	90.6	ND	187
Pheniramine	230109	241.1699	0.71	10.4	0.03	20	72	69.1	0.74	109.8	2.9	181
Pheniramine	230424	241.1699	0.52	10.4	0.02	20	86	97.8	0.9	103	6.8	45
Pheniramine	230711	241.1699	0.19	10.7	0.04	20	63	76	1.01	102.7	4.9	100
Pheniramine	231106	241.1699	2.02	10.4	0	20	52	60	0.76	90.7	15.4	45
Pheniramine	240131	241.1699	1.34	10.6	0.01	20	66	85	0.68	97.4	7.4	127
Pheniramine	240701	241.1699	1.07	11.5	0.02	20	53	73.4	0.85	88.3	5.5	143
Sulfamethoxazole	220613	254.0594	0.41	13	0.01	25	231	97.3	0.62	120.4	13.9	148
Sulfamethoxazole	220623	254.0594	0.30	13.1	0.01	25	128	84.2	0.7	105.2	5.7	38
Sulfamethoxazole	220704	254.0594	0.25	13.1	0.02	25	160	78.3	0.68	101.8	10.2	143

Substance	Measurement batch ID	m/z theoretical [M+H]+I	m/z delta [ppm]	RT measured [min]	SD RT [min]	LOQ [ng/L]	Sample conc. [ng/L]	Samples above LOQ [%]	Absolute recovery	Relative recovery [%]	Precision [%]	Number of samples
Sulfamethoxazole	220803	254.0594	-0.03	13.1	0.01	25	84	88.6	0.68	98.4	ND	79
Sulfamethoxazole	221021	254.0594	0.30	12.9	0.01	25	100	81.5	0.71	103.3	ND	184
Sulfamethoxazole	230109	254.0594	0.40	12.6	0.02	25	244	94.5	0.58	106.7	16	181
Sulfamethoxazole	230424	254.0594	0.27	12.6	0.01	25	253	97.8	0.61	99.1	15.7	45
Sulfamethoxazole	230711	254.0594	0.05	12.9	0.01	25	275	97	0.6	104.4	8.8	99
Sulfamethoxazole	231106	254.0594	1.43	12.9	0.02	25	251	97.8	0.58	93.8	1.5	46
Sulfamethoxazole	240131	254.0594	0.99	12.9	0.02	25	312	93.7	0.66	105.4	4	127
Sulfamethoxazole	240701	254.0594	0.79	13	0.02	25	278	96.5	0.62	92.8	5	142
Tramadol	220613	264.1958	0.43	11.8	0.03	20	374	100	0.82	103.7	5.6	148
Tramadol	220623	264.1958	0.31	11.8	0.03	20	395	100	0.93	102.4	1.6	38
Tramadol	220704	264.1958	0.15	11.9	0.05	20	235	99.3	0.82	101.1	1.5	143
Tramadol	220803	264.1958	-0.10	12	0.03	20	242	100	0.8	101.5	ND	79
Tramadol	221021	264.1958	0.20	11.7	0.04	20	184	100	0.83	99.8	ND	184
Tramadol	230109	264.1958	0.45	11.5	0.04	20	269	100	0.84	100.1	2.3	181
Tramadol	230424	264.1958	0.27	11.6	0.04	20	245	100	0.87	97.9	4.3	45
Tramadol	230711	264.1958	0.17	12	0.04	20	293	100	0.89	102	1	99
Tramadol	231106	264.1958	1.39	11.6	0.03	20	211	100	0.88	96.1	0.6	46
Tramadol	240131	264.1958	0.90	11.7	0.04	20	315	99.2	0.86	101	2.2	127
Tramadol	240701	264.1958	0.69	12	0.07	20	237	99.3	0.81	90.3	0.6	142
Trimethoprim	220613	291.1452	0.47	10.3	0.02	30	198	100	0.8	103	6.1	148
Trimethoprim	220623	291.1452	0.34	10.3	0.02	30	160	97.4	0.93	104.3	2.7	38
Trimethoprim	220704	291.1452	0.11	10.3	0.03	30	159	97.9	0.86	103.7	1.6	143
Trimethoprim	220803	291.1452	0.17	10.4	0.02	30	167	100	0.87	101	ND	79
Trimethoprim	221021	291.1452	0.32	10.1	0.02	30	137	98.4	0.89	98.3	ND	184
Trimethoprim	230109	291.1452	1.05	10	0.03	30	134	99.4	0.74	96.8	5	181
Trimethoprim	230424	291.1452	0.69	10	0.03	30	154	100	0.83	98.7	6.8	45
Trimethoprim	230711	291.1452	0.59	10.4	0.03	30	171	98	0.81	101.6	2	99
Trimethoprim	231106	291.1452	1.99	10	0.03	30	146	100	0.75	90.5	3.3	46
Trimethoprim	240131	291.1452	1.66	10.1	0.03	30	126	96.9	0.82	101.3	5.1	127
Trimethoprim	240701	291.1452	0.57	10.4	0.05	30	163	98.6	0.76	93.2	3.7	142

SI 2.2 **Spatiotemporal patterns of chemical and viral loads in wastewater**

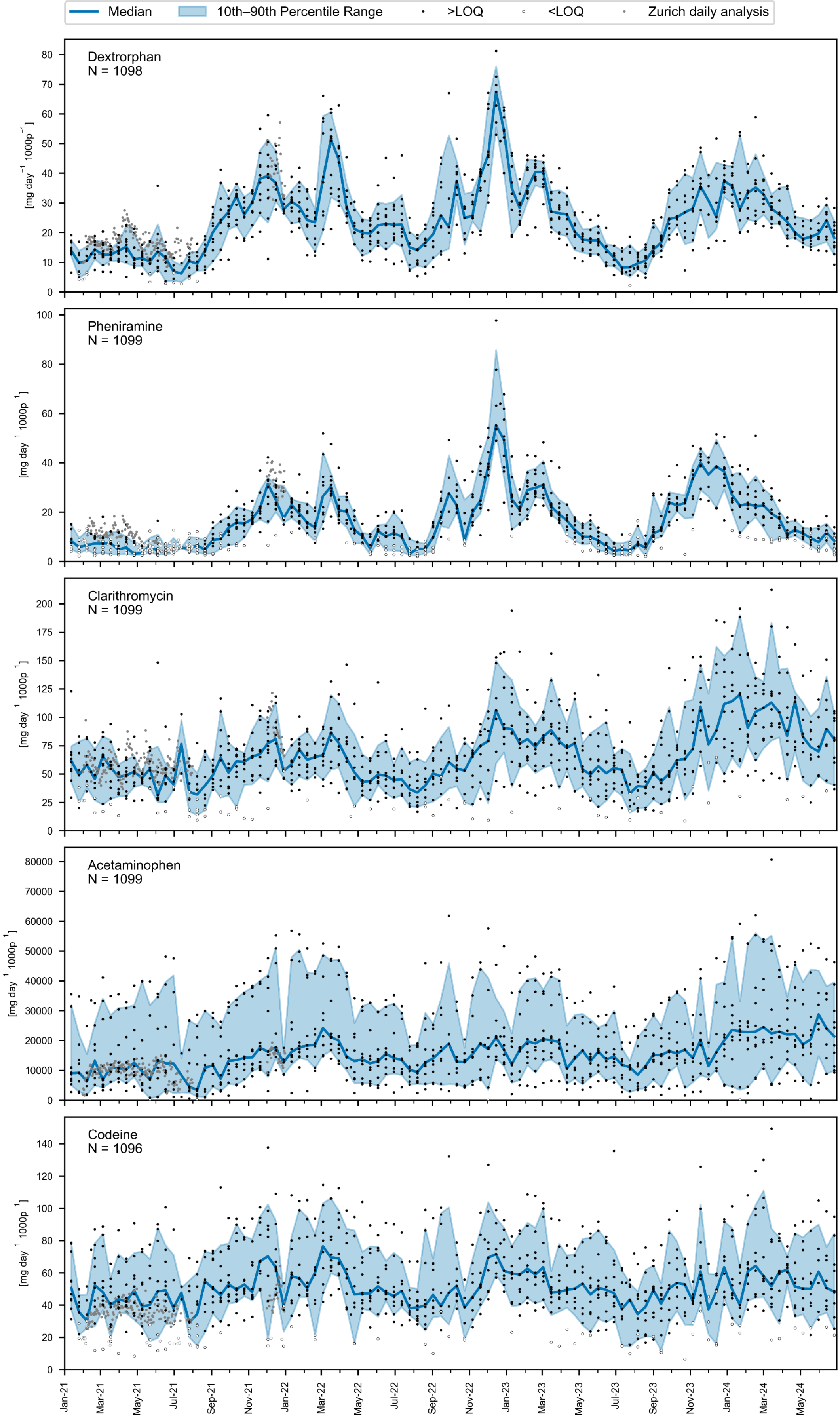

Figure SI 7: Spatiotemporal patterns of pharmaceutical loads in wastewater. Pharmaceutical loads from 10 wastewater treatment plants, sampled every 13th day from 2021 to June 2024, are represented by black dots. Grey dots indicate daily samples from Zurich, collected between February to July 2021 and in December 2021, highlighting day-to-day variability. Samples with values below the limit of quantification (LOQ) are depicted as white dots, with these loads estimated from concentration of 1/2 * LOQ. Day-specific medians are illustrated by interconnected blue dots, with shaded areas indicating the 10th to 90th interpercentile range.

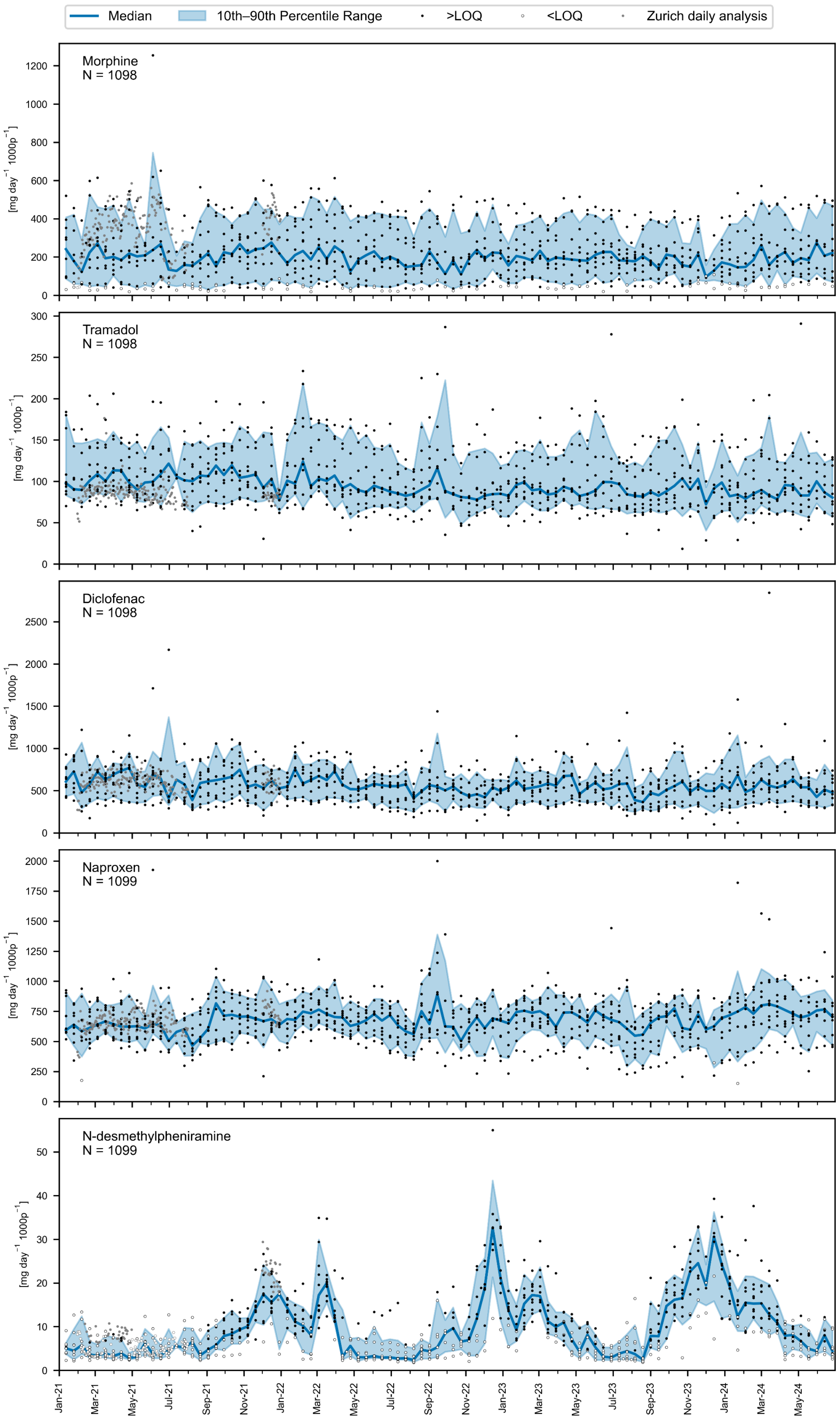

Figure SI 8: Spatiotemporal patterns of pharmaceutical loads in wastewater. Pharmaceutical loads from 10 wastewater treatment plants, sampled every 13th day from 2021 to June 2024, are represented by black dots. Grey dots indicate daily samples from Zurich, collected between February to July 2021 and in December 2021, highlighting day-to-day variability. Samples with values below the limit of quantification (LOQ) are depicted as white dots, with these loads estimated from concentration of 1/2 * LOQ. Day-specific medians are illustrated by interconnected blue dots, with shaded areas indicating the 10th to 90th interpercentile range.

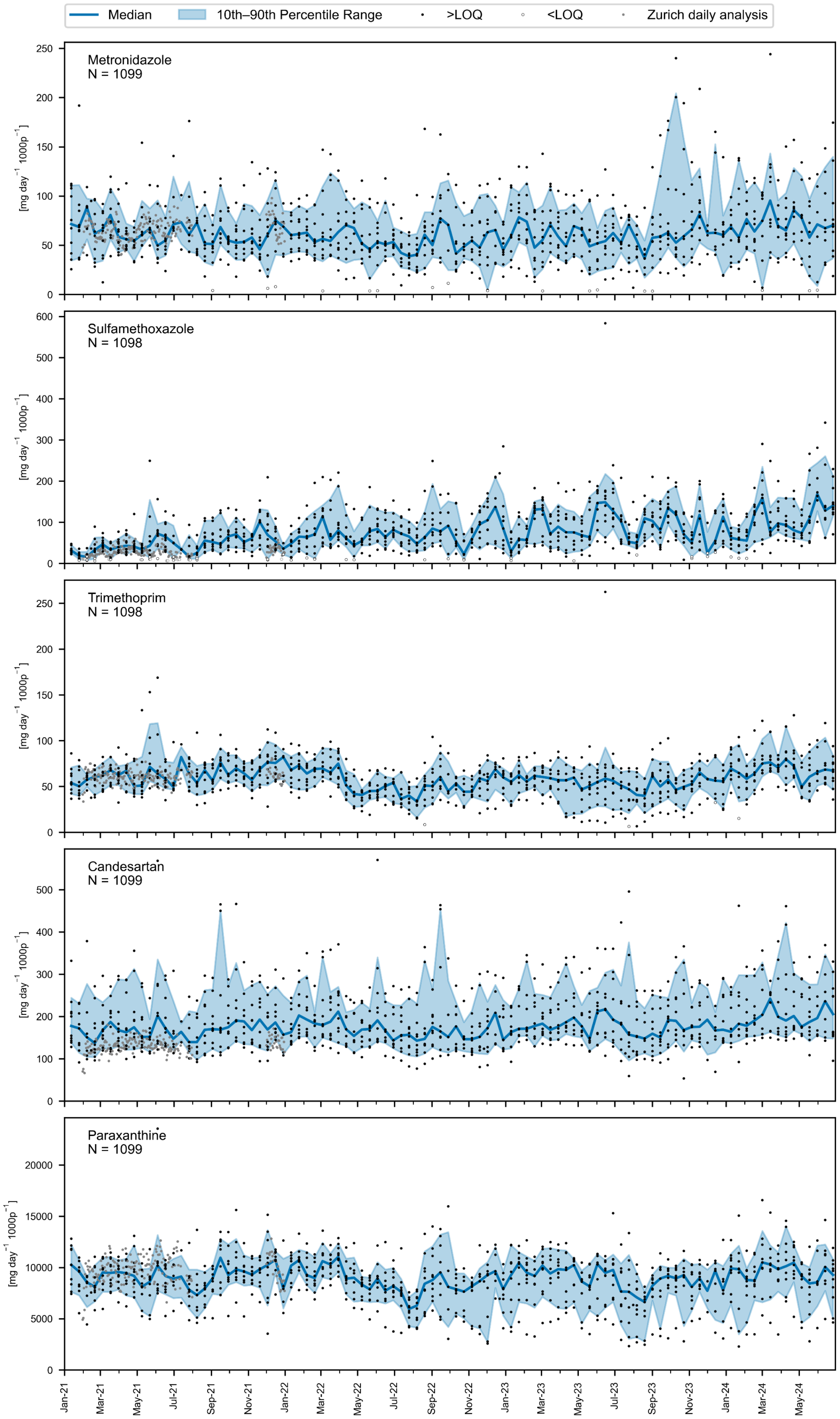

Figure SI 9: Spatiotemporal patterns of pharmaceutical loads in wastewater. Pharmaceutical loads from 10 wastewater treatment plants, sampled every 13th day from 2021 to June 2024, are represented by black dots. Grey dots indicate daily samples from Zurich, collected between February to July 2021 and in December 2021, highlighting day-to-day variability. Samples with values below the limit of quantification (LOQ) are depicted as white dots, with these loads estimated from concentration of 1/2 * LOQ. Day-specific medians are illustrated by interconnected blue dots, with shaded areas indicating the 10th to 90th interpercentile range.

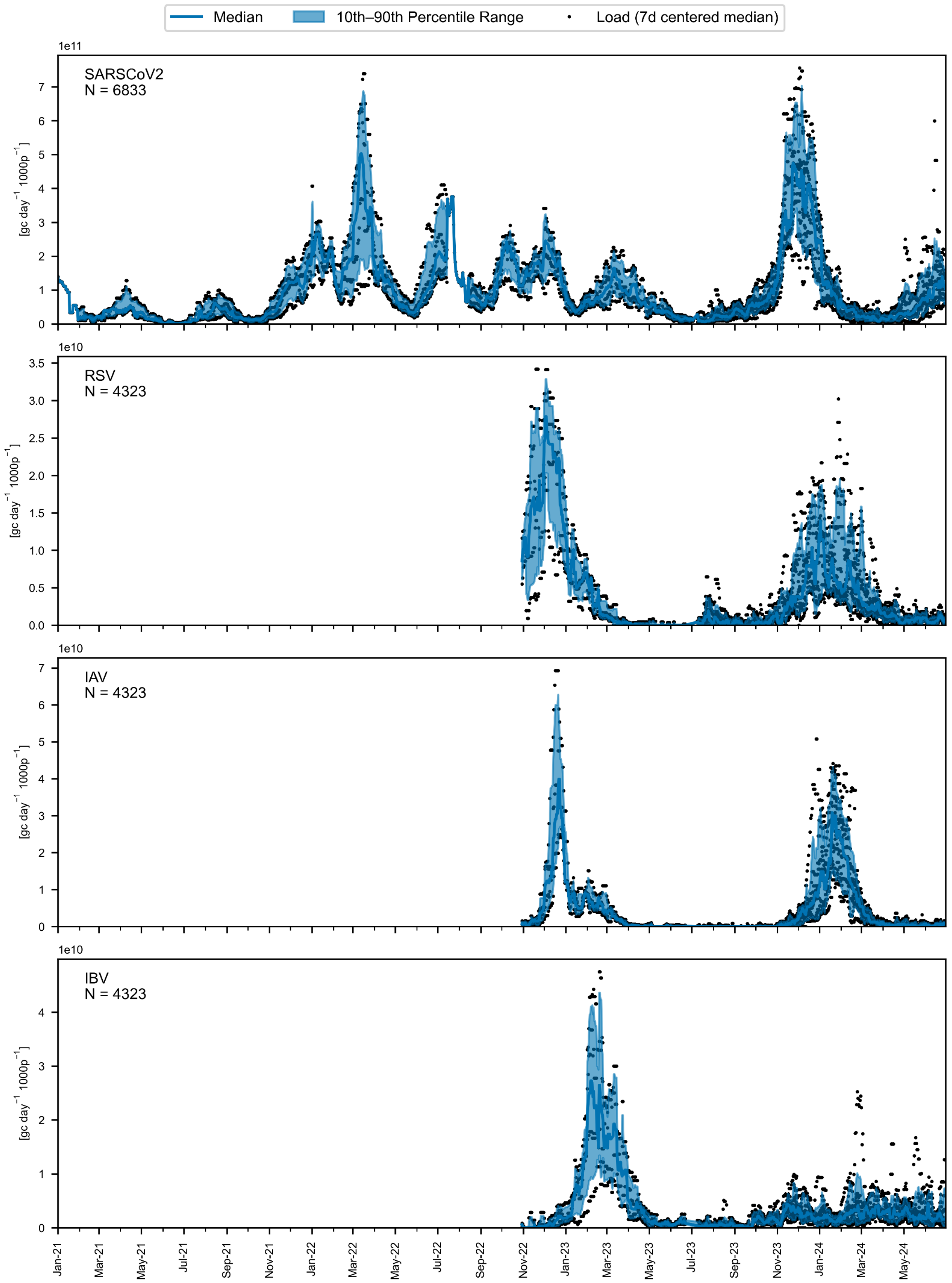

Figure SI 10: Spatiotemporal patterns of viral loads in wastewater. Day-specific gene copy median loads from SARS-CoV-2, RSV, influenza A (IAV) and influenza B (IBV) are illustrated by interconnected blue dots. Shaded areas indicate the 10th to 90th interpercentile range.

SI 2.2.1 **Spatiotemporal pattern of baseline-subtracted loads for pharmaceuticals**

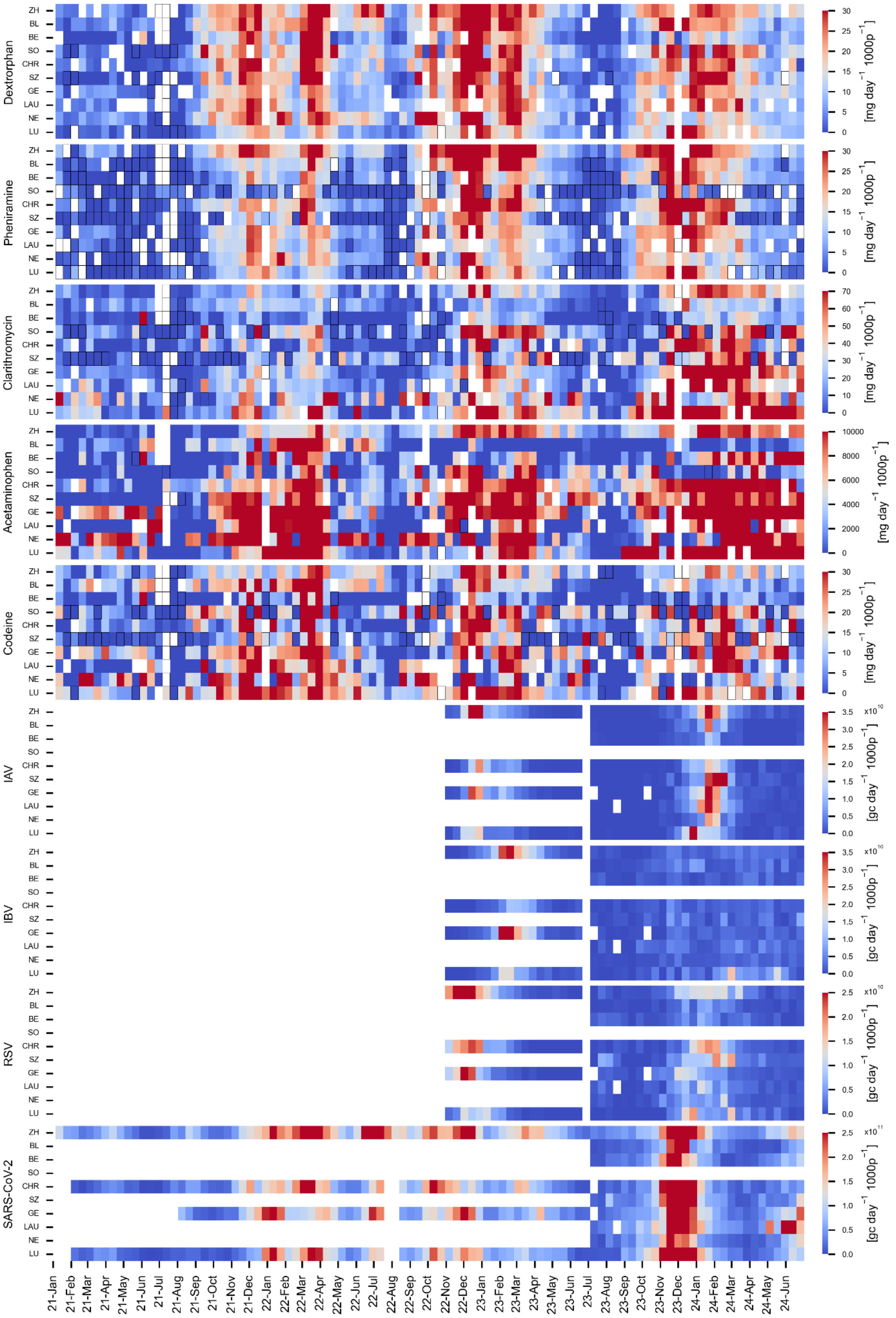

Figure SI 11: Spatiotemporal load patterns of baseline-subtracted pharmaceutical loads and viral gene copy loads in wastewater. Wastewater loads from every 13th day between January 2021 and June 2024 are displayed. Data is shown for individual treatment plants according to the abbreviations on the Y-axis: ZH (Zurich), BL (Basel), BE (Bern), SO (Solothurn), CHR (Chur), SZ (Schwyz), GE (Geneva), LA (Lausanne), NE (Neuchâtel), and LU (Lugano). These are arranged first by language region, then by catchment population size. The coloring represents the load values for pharmaceuticals [mg d^{-1} $1000p^{-1}d^{-1}$] and viruses [gc d^{-1} $1000p^{-1}$] with linear color scaling. For pharmaceuticals, each space refers to individual samples, and for the viral loads, each space reflects values from the centered 7-day median. White space indicates data exclusions due to day-specific wastewater volumes exceeding maximum threshold values or due to lack of sampling. Black-outlined spaces represent pharmaceutical loads from samples with concentrations below the limit of quantification (LOQ), which are estimated based on half the LOQ value. . Baseline loads were determined from location-specific mean values during periods of low respiratory virus exposure (June 2021 and July to August 2023).

SI 2.2.2 **Influenza subtype clinical surveillance data**

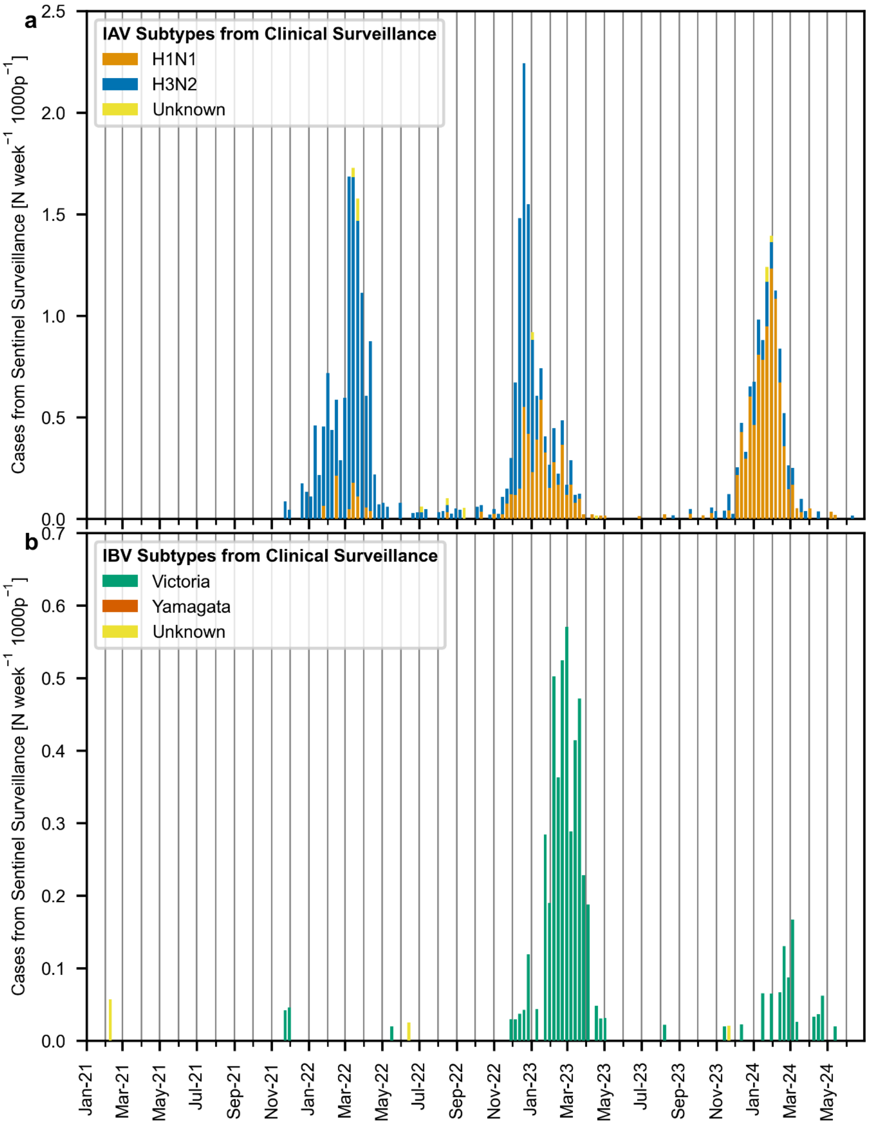

Figure SI 12: Temporal trends in national influenza subtypes from Swiss sentinel surveillance (Sentinella). (a) Case numbers of Influenza A (IAV) subtypes and (b) case numbers of Influenza B (IBV) subtypes.

SI 2.2.3 **Temporal trends of cases from mandatory reporting system and sentinel consultations**

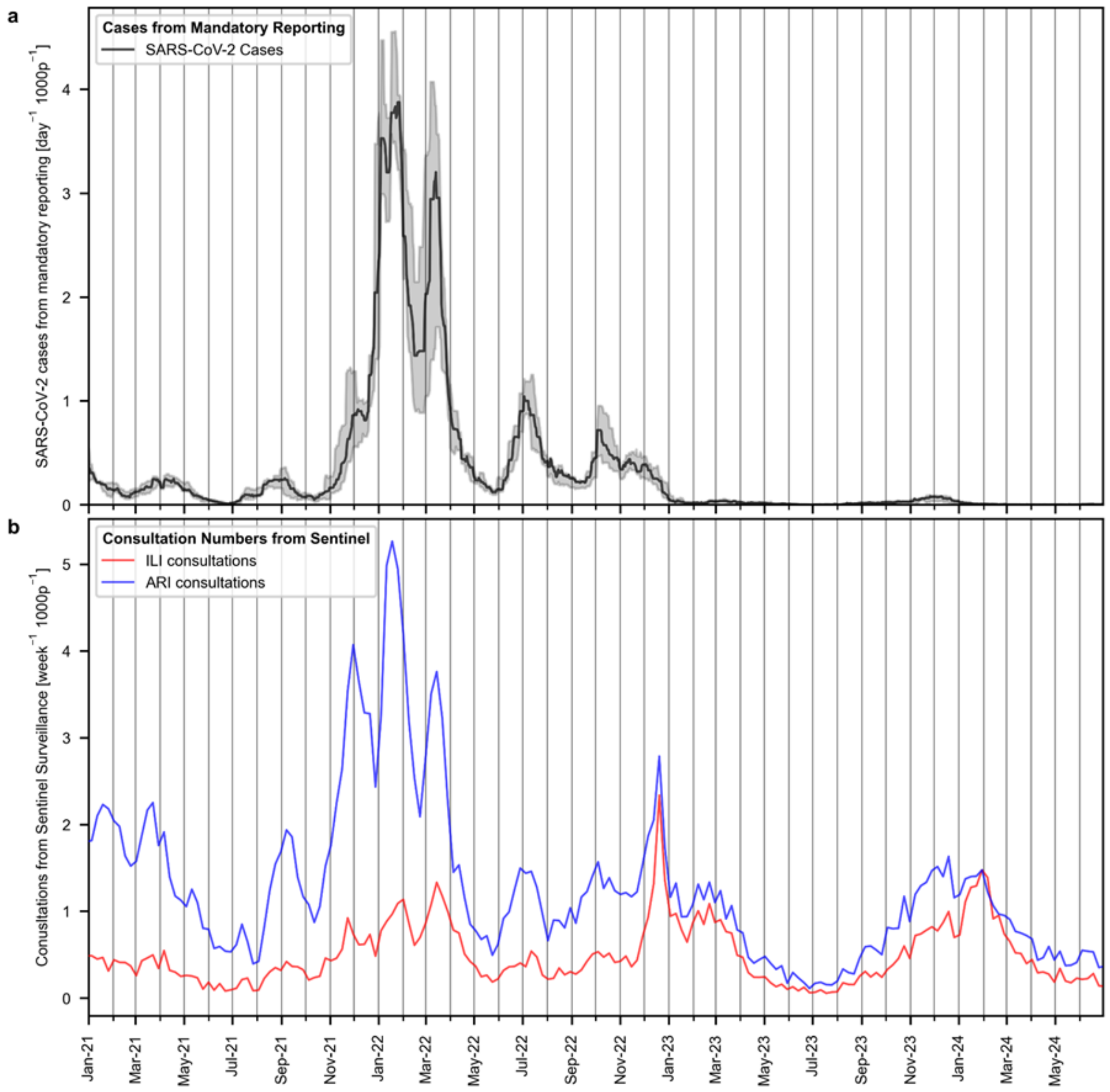

Figure SI 13: National level trends of SARS-COV-2 cases and sentinel consultations. (a) Median values of reported cases from the Swiss mandatory SARS-CoV-2 reporting system, based on the catchment areas of Zurich, Chur, Geneva, and Lugano, with the 10% to 90% interpercentile range represented by shaded areas. (b) General practitioner-based Sentinella data on weekly primary consultations for influenza-like illness (ILI) or acute respiratory infection (ARI) symptoms.

SI 2.2.4 Distribution of respiratory viruses from sentinel clinical testing

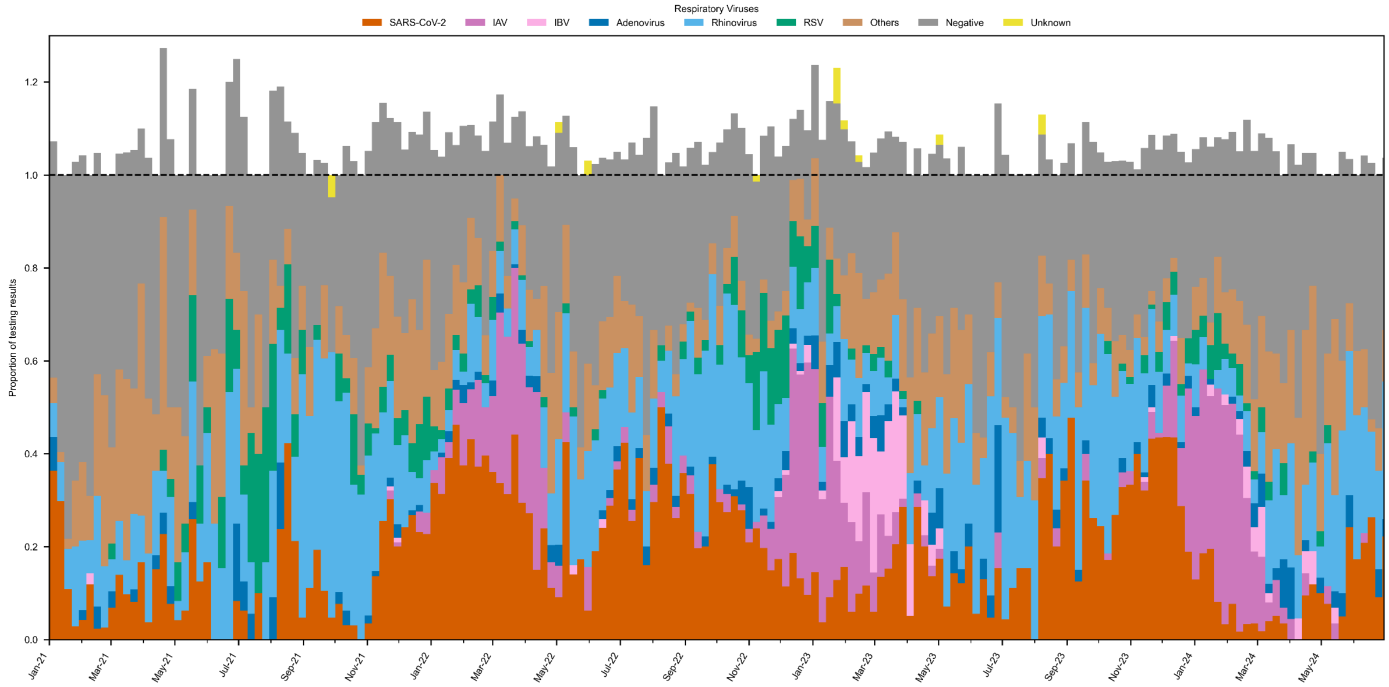

Figure SI 14: Proportion of positive tests for respiratory viruses from the Swiss sentinel surveillance system. This figure represents the proportion of positive test results for each virus, calculated by dividing the number of detections by the total number of samples tested. The "Others" category comprises detections of bocavirus, human coronaviruses (229E, HKU1, NL63, OC43), metapneumovirus, and parainfluenza viruses (types 1-4). The "Negative" category includes cases where individuals presented with symptoms of acute respiratory infection (ARI) or influenza-like illness (ILI) but tested negative for all surveillanced respiratory viruses. The "Unknown" category reflects samples with incomplete data regarding detected respiratory viruses.

The summed proportion may exceed 1 when samples test positive for multiple respiratory viruses. To account for symptom burden, we assume that co-infections are associated with more severe symptoms. Therefore, we calculate the proportion based on the total number of samples rather than the total number of detections.

SI 2.3 Correlation analysis of wastewater markers

SI 2.3.1 Pearson correlations chemical and viral loads (location-specific)

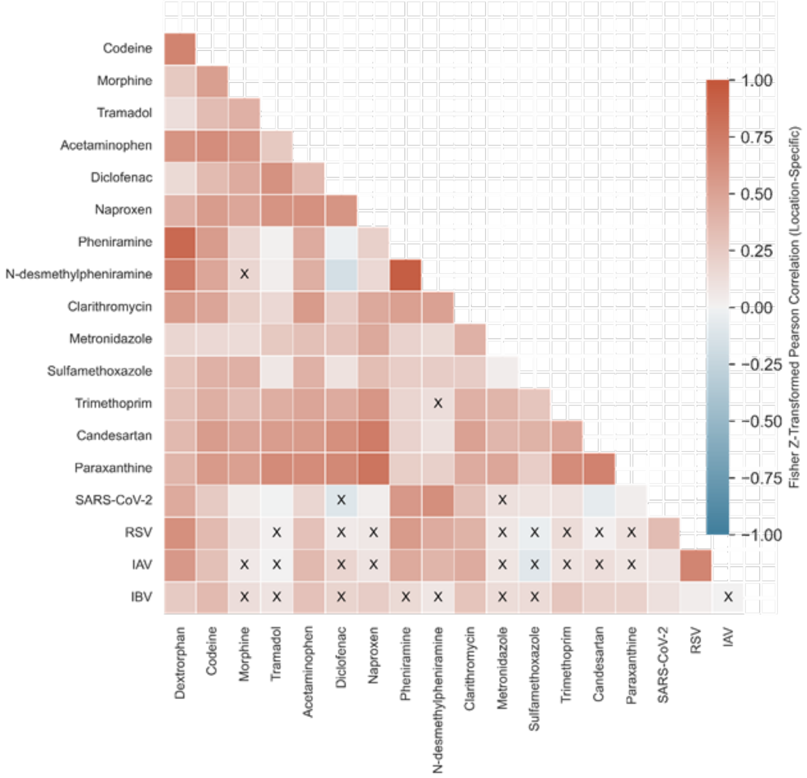

Figure SI 15: Fisher Z-transformed location-specific Pearson correlations of wastewater population loads. Correlations between pairs of wastewater markers were calculated for each of the ten locations and then aggregated using Fisher's Z-transformation. For chemical markers, loads from individual samples collected every 13th day were used, while for viruses, the 7-day centered median of daily values was employed. Correlation values with non-significant p-values (defined here as above 0.01) are marked with a cross. All wastewater pharmaceutical data with concentrations below the limit of quantification (LOQ) were excluded from the analysis.

SI 2.3.2 **Pearson correlations chemical and viral loads (location independent)**

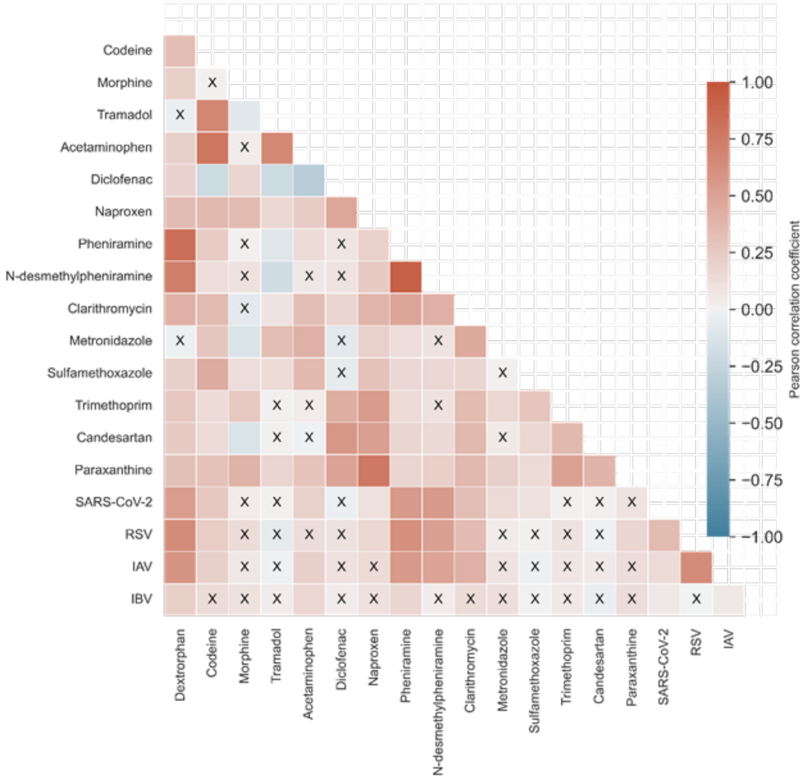

Figure SI 16: Pearson correlations of wastewater population loads. Correlations between pairs of wastewater markers were calculated across all data, independent of location. For pharmaceuticals, loads from individual samples collected every 13th day were used, while for viruses, the 7-day centered median of daily values was employed. Correlation values with p-values above 0.01 are marked with a cross. All wastewater pharmaceutical data with concentrations below the limit of quantification (LOQ) were excluded from the analysis.

SI 2.3.3 **Pearson correlations baseline-subtracted chemical and viral loads (location independent)**

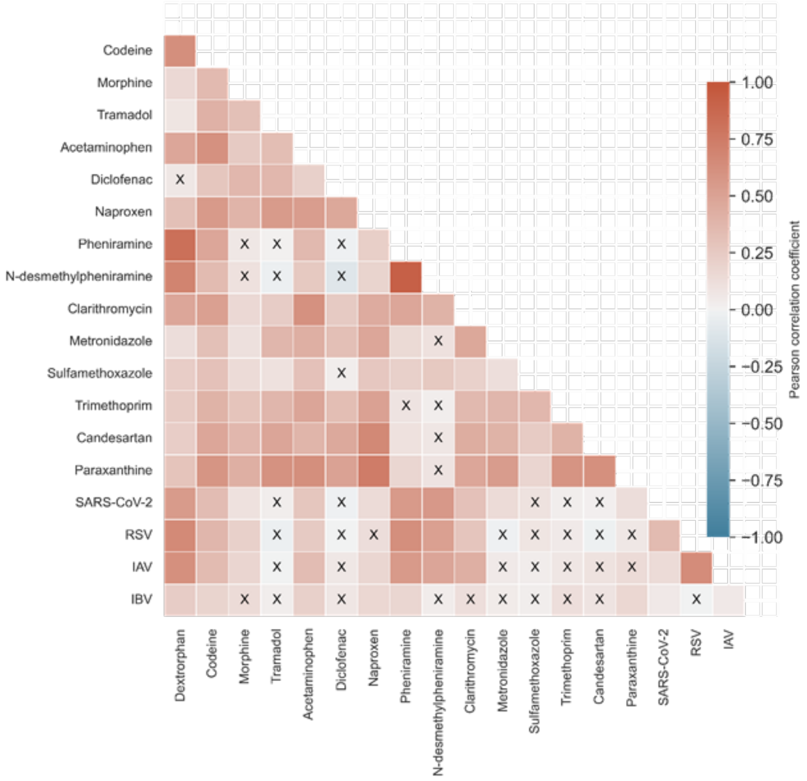

Figure SI 17: Pearson correlations of wastewater population loads. Correlations between pairs of wastewater markers were calculated across all data, independent of location. For pharmaceuticals, baseline-subtracted loads from individual samples collected every 13th day were used, while for viruses, the 7-day centered median of daily values was employed. Correlation values with p-values above 0.01 are marked with a cross. All wastewater pharmaceutical data with concentrations below the limit of quantification (LOQ) were excluded from the analysis. Baseline loads were determined from location-specific mean values during periods of low respiratory virus exposure (June 2021 and July to August 2023).

SI 2.3.4 **Scatter plot of viral and pharmaceutical loads (location independent)**

Correlation of SARS-CoV-2 with pharmaceutical loads in wastewater

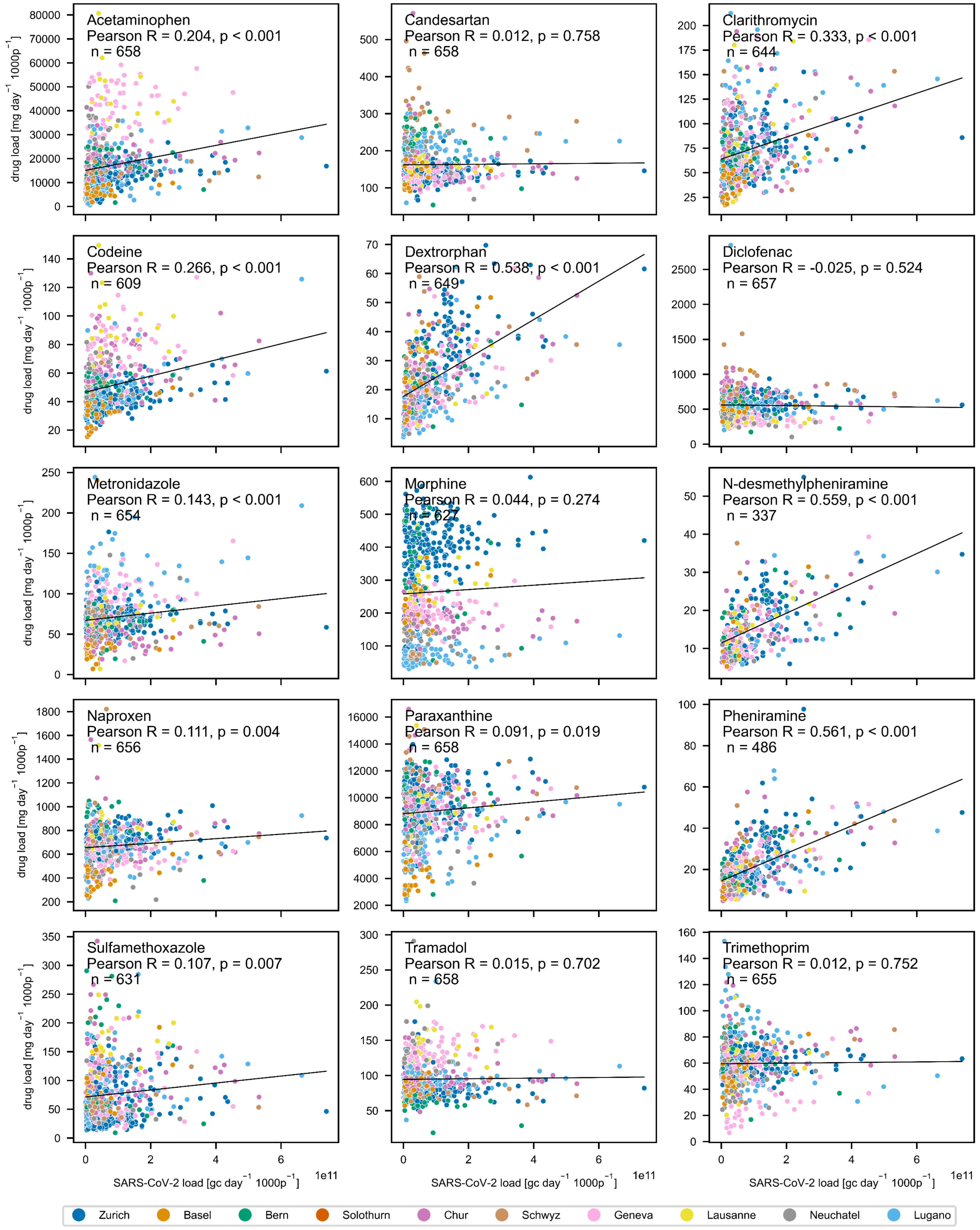

Figure SI 18: Scatterplot showing the correlation between SARS-COV-2 gene copy loads and chemical marker loads in wastewater, with data points colored by location. Pearson correlation coefficients (r), two-sided p-values, and the number of observations (n) are indicated for each marker.

Correlation of RSV with Pharmaceutical Loads in Wastewater

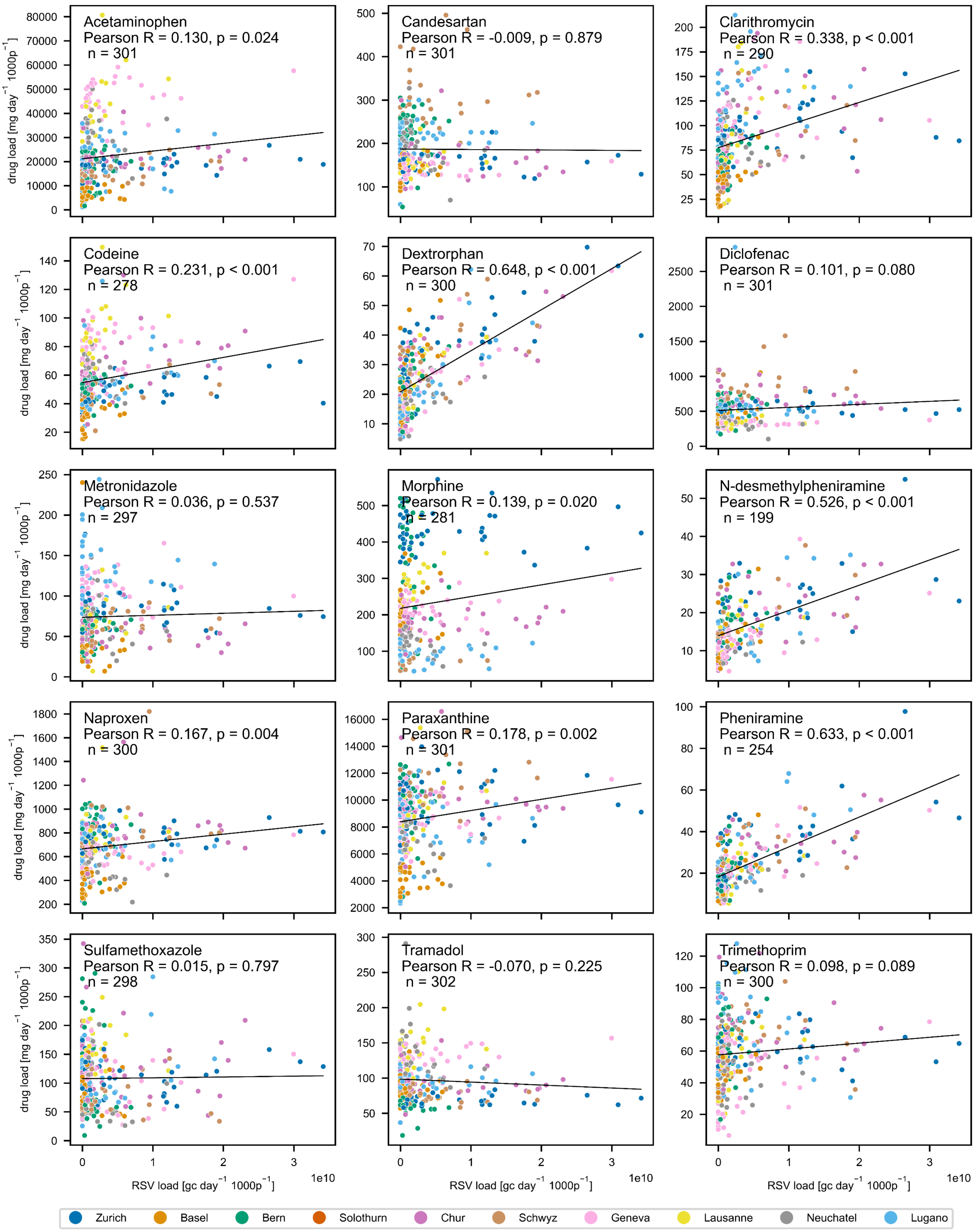

Figure SI 19: Scatterplot showing the correlation between RSV gene copy loads and chemical marker loads in wastewater, with data points colored by location. Pearson correlation coefficients (R), two-sided p-values, and the number of observations (n) are indicated for each marker.

Correlation of Influenza A with Pharmaceutical Loads in Wastewater

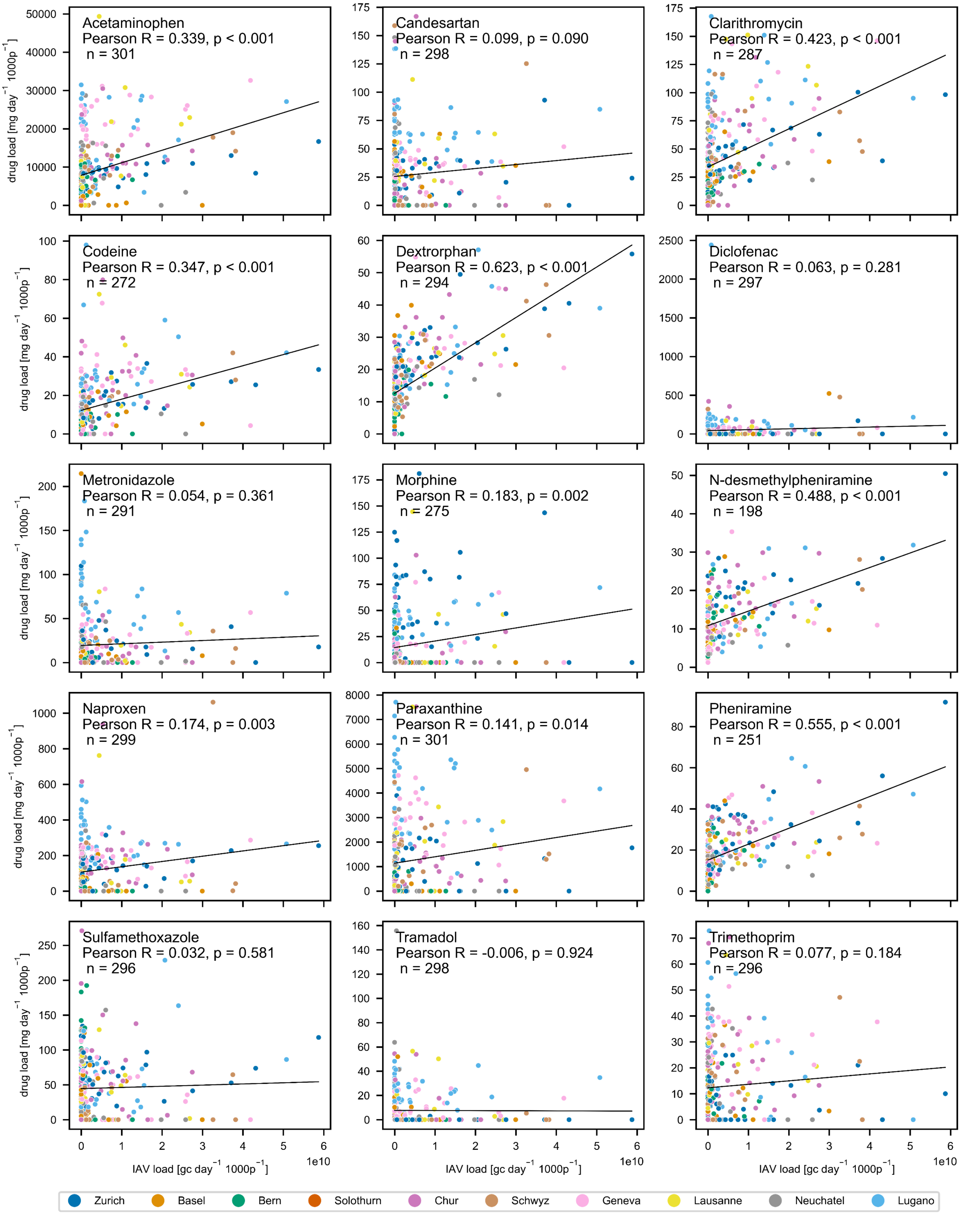

Figure SI 20: Scatterplot showing the correlation between Influenza A gene copy loads and chemical marker loads in wastewater, with data points colored by location. Pearson correlation coefficients (R), two-sided p-values, and the number of observations (n) are indicated for each marker.

Correlation of Influenza B with Pharmaceutical Loads in Wastewater

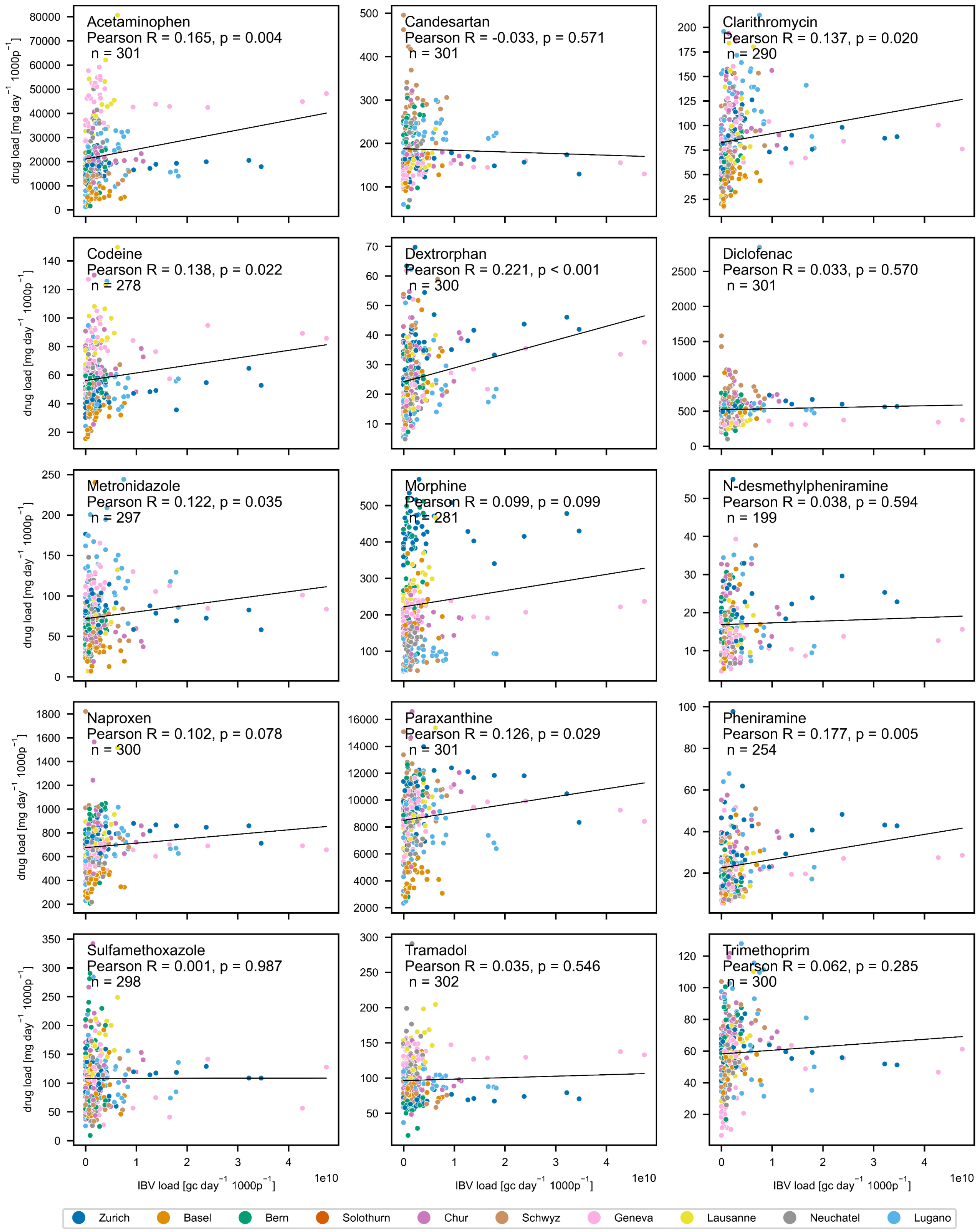

Figure SI 21: Scatterplot showing the correlation between Influenza B gene copy loads and chemical marker loads in wastewater, with data points colored by location. Pearson correlation coefficients (R), two-sided p-values, and the number of observations (n) are indicated for each marker.

SI 2.4 **Modeling of relationship between pharmaceutical and viral loads in wastewater**

Ordinary Least Squares (OLS) regression was used to estimate the coefficients of the linear model. However, the calculated statistics, including p-values and standard errors, should be interpreted with caution, as the use of time-series data violates the independence assumption inherent in OLS regression. To mitigate potential issues of heteroscedasticity and autocorrelation in the residuals, we applied Newey-West heteroskedasticity and autocorrelation consistent (HAC) standard errors with a maximum lag of 5. While this adjustment improves the reliability of the estimated standard errors, some degree of serial dependence may still persist, and results should be interpreted accordingly.

SI 2.4.1 **Ordinary least squares analyses with viral loads as predicting variables**

OLS diagnostics

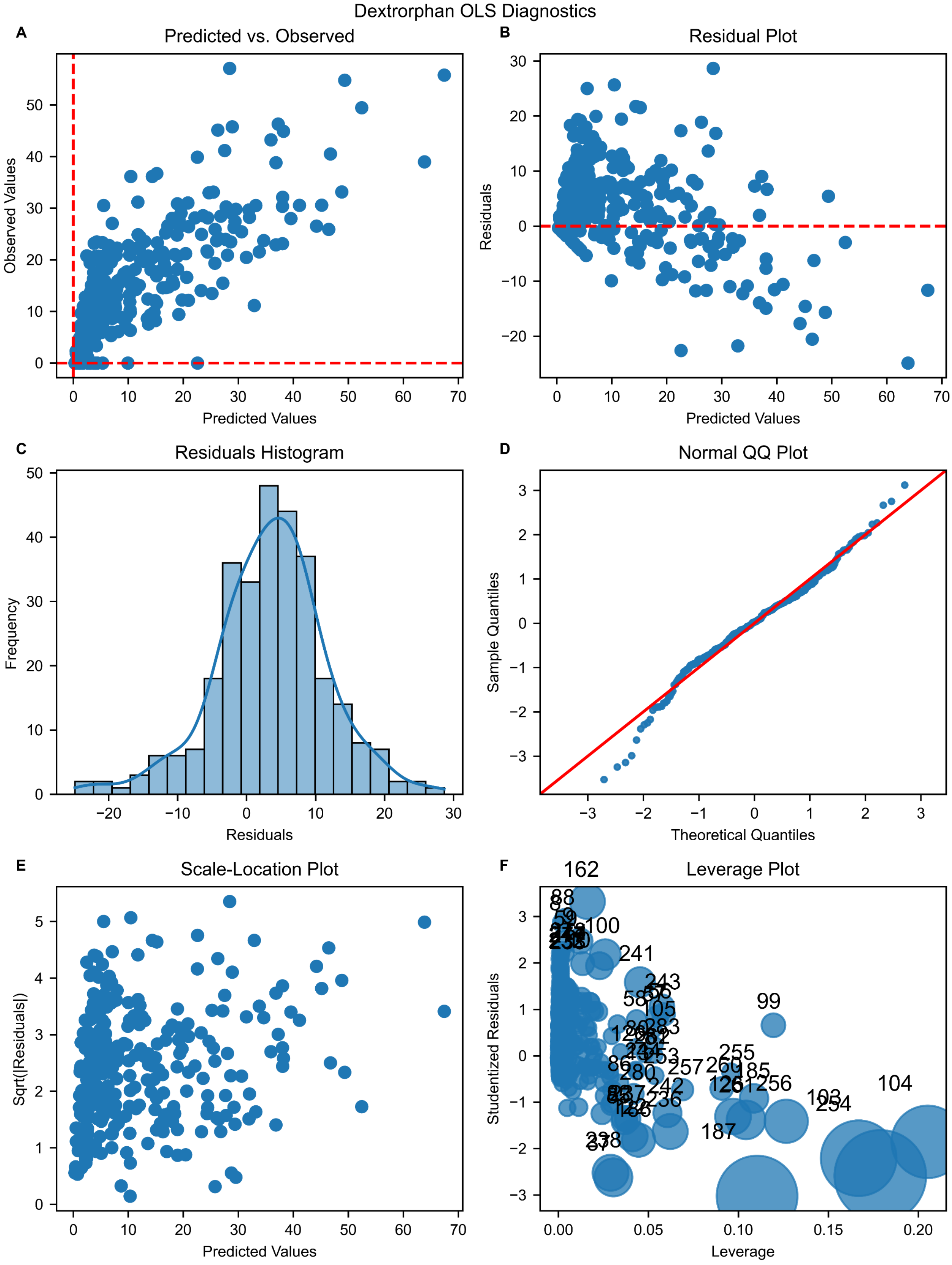

Figure SI 22: Diagnostic plots for the Ordinary Least Squares (OLS) regression analysis using 7-day centered median gene copy number loads of SARS-CoV-2, RSV, IAV, and IBV as predictors, with baseline-subtracted dextrorphan loads as the response variable. The figure includes: (A) Predicted vs. Observed Values, depicting the correlation between predicted and observed pharmaceutical loads; (B) Residual Plot, illustrating residuals against predicted values to assess homoscedasticity; (C) Residuals Histogram, showing the residual distribution with an overlaid kernel density estimate; (D) Normal Q-Q Plot, evaluating the normality of residuals; (E) Scale-Location Plot, revealing the spread of residuals to detect non-constant variance; and (F) Leverage Plot, highlighting influential data points using Cook's distance.

Table SI 5: Ordinary Least Squares (OLS) regression results using gene copy loads of SARS-COV-2, RSV, IAV and IBV as input as predicting variable and baseline-subtracted dextrorphan loads as the response variable. The time-series nature of the data is addressed using a heteroskedasticity- and autocorrelation-consistent (HAC) covariance matrix estimator, but the associated p-values and confidence intervals should still be interpreted with caution.

```
                                 OLS Regression Results                                
=======================================================================================
Dep. Variable:            Dextrorphan   R-squared (uncentered):                   0.796
Model:                            OLS   Adj. R-squared (uncentered):              0.793
Method:                 Least Squares   F-statistic:                              101.0
Date:                Tue, 11 Feb 2025   Prob (F-statistic):                    8.55e-54
Time:                        10:18:01   Log-Likelihood:                         -1059.5
No. Observations:                 295   AIC:                                      2127.
Df Residuals:                     291   BIC:                                      2142.
Df Model:                           4                                                  
Covariance Type:                  HAC                                                  
==============================================================================
                 coef    std err          z      P>|z|      [0.025      0.975]
------------------------------------------------------------------------------
SARSCoV2    4.716e-11   6.96e-12      6.778      0.000    3.35e-11    6.08e-11
RSV         1.016e-09   1.66e-10      6.112      0.000     6.9e-10    1.34e-09
IAV         4.555e-10   8.81e-11      5.171      0.000    2.83e-10    6.28e-10
IBV         7.838e-10   1.38e-10      5.687      0.000    5.14e-10    1.05e-09
==============================================================================
Omnibus:                       13.243   Durbin-Watson:                   0.909
Prob(Omnibus):                  0.001   Jarque-Bera (JB):               20.882
Skew:                          -0.291   Prob(JB):                     2.92e-05
Kurtosis:                       4.166   Cond. No.                         35.9
==============================================================================

Notes:
[1] R² is computed without centering (uncentered) since the model does not contain a constant.
[2] Standard Errors are heteroscedasticity and autocorrelation robust (HAC)
using 5 lags and without small sample correction
```

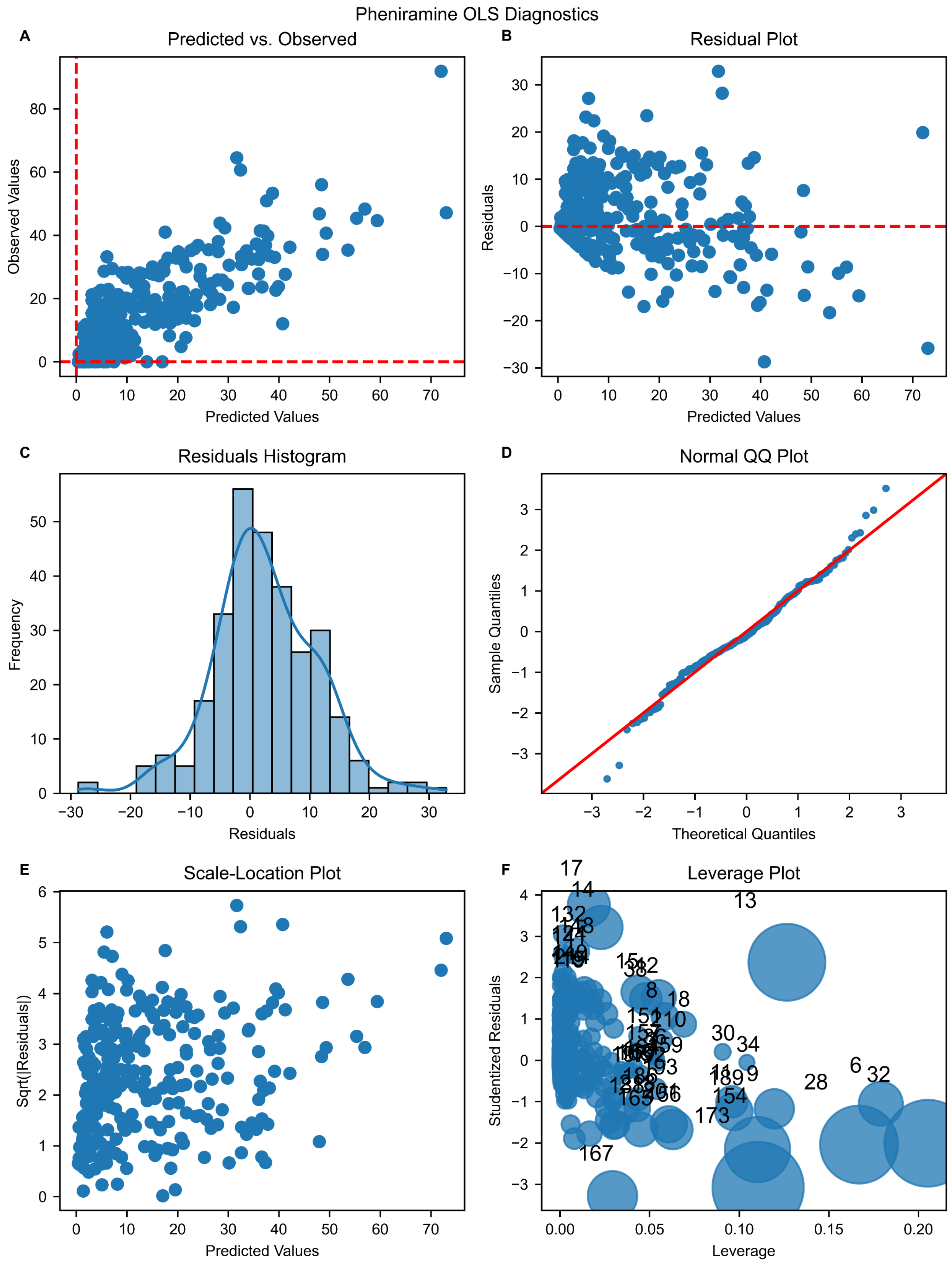

Figure SI 23: Diagnostic plots for the Ordinary Least Squares (OLS) regression analysis using 7-day centered median gene copy number loads of SARS-CoV-2, RSV, IAV, and IBV as predictors, with baseline-subtracted pheniramine loads as the response variable. The figure includes: (A) Predicted vs. Observed Values, depicting the correlation between predicted and observed pharmaceutical loads; (B) Residual Plot, illustrating residuals against predicted values to assess homoscedasticity; (C) Residuals Histogram, showing the residual distribution with an overlaid kernel density estimate; (D) Normal Q-Q Plot, evaluating the normality of residuals; (E) Scale-Location Plot, revealing the spread of residuals to detect non-constant variance; and (F) Leverage Plot, highlighting influential data points using Cook's distance.

Table SI 6: Ordinary Least Squares (OLS) regression results using gene copy loads of SARS-COV-2, RSV, IAV and IBV as input as predicting variable and baseline-subtracted pheniramine loads as the response variable. The time-series nature of the data is addressed using a heteroskedasticity- and autocorrelation-consistent (HAC) covariance matrix estimator, but the associated p-values and confidence intervals should still be interpreted with caution.

```
                                 OLS Regression Results                                
=======================================================================================
Dep. Variable:            Pheniramine   R-squared (uncentered):                   0.823
Model:                            OLS   Adj. R-squared (uncentered):              0.821
Method:                 Least Squares   F-statistic:                              99.73
Date:                Tue, 11 Feb 2025   Prob (F-statistic):                    3.19e-53
Time:                        10:18:24   Log-Likelihood:                         -1058.8
No. Observations:                 293   AIC:                                      2126.
Df Residuals:                     289   BIC:                                      2140.
Df Model:                           4                                                  
Covariance Type:                  HAC                                                  
==============================================================================
                 coef    std err          z      P>|z|      [0.025      0.975]
------------------------------------------------------------------------------
SARSCoV2      7.19e-11   8.27e-12      8.692      0.000    5.57e-11    8.81e-11
RSV         9.345e-10   1.71e-10      5.461      0.000    5.99e-10    1.27e-09
IAV          4.69e-10   1.28e-10      3.658      0.000    2.18e-10     7.2e-10
IBV         6.366e-10   1.51e-10      4.207      0.000     3.4e-10    9.33e-10
==============================================================================
Omnibus:                        7.410   Durbin-Watson:                   0.887
Prob(Omnibus):                  0.025   Jarque-Bera (JB):               12.107
Skew:                           0.052   Prob(JB):                      0.00235
Kurtosis:                       3.991   Cond. No.                         35.8
==============================================================================

Notes:
[1] R² is computed without centering (uncentered) since the model does not contain a constant.
[2] Standard Errors are heteroscedasticity and autocorrelation robust (HAC)
using 5 lags and without small sample correction
```

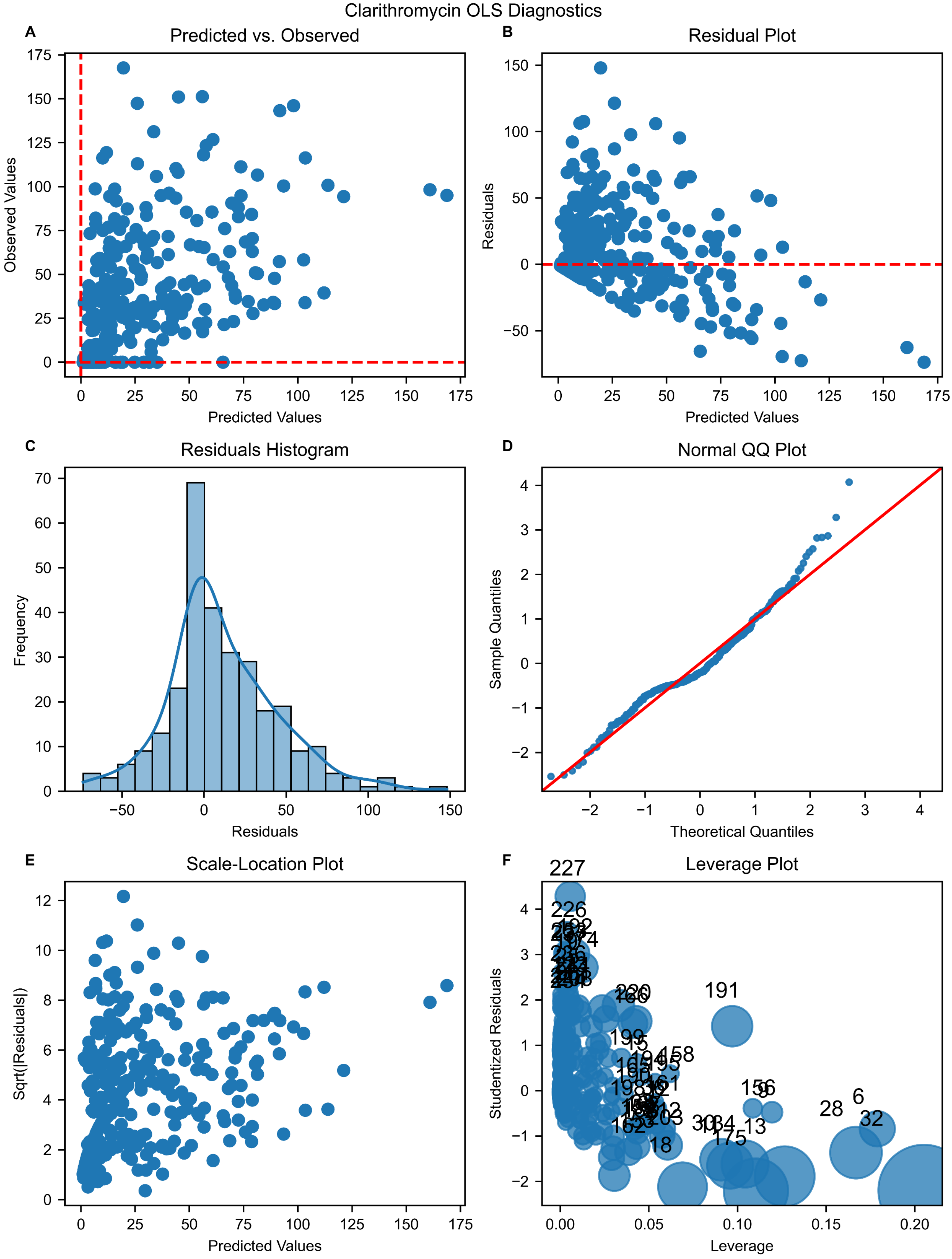

Figure SI 24: Diagnostic plots for the Ordinary Least Squares (OLS) regression analysis using 7-day centered median gene copy number loads of SARS-CoV-2, RSV, IAV, and IBV as predictors, with baseline-subtracted pharmaceutical loads as the response variable. The figure includes: (A) Predicted vs. Observed Values, depicting the correlation between predicted and observed pharmaceutical loads; (B) Residual Plot, illustrating residuals against predicted values to assess homoscedasticity; (C) Residuals Histogram, showing the residual distribution with an overlaid kernel density estimate; (D) Normal Q-Q Plot, evaluating the normality of residuals; (E) Scale-Location Plot, revealing the spread of residuals to detect non-constant variance; and (F) Leverage Plot, highlighting influential data points using Cook's distance.

Table SI 7: Ordinary Least Squares (OLS) regression results using gene copy loads of SARS-COV-2, RSV, IAV and IBV as input as predicting variable and baseline-subtracted clarithromycine loads as the response variable. The time-series nature of the data is addressed using a heteroskedasticity- and autocorrelation-consistent (HAC) covariance matrix estimator, but the associated p-values and confidence intervals should still be interpreted with caution.

```
                                 OLS Regression Results                                
=======================================================================================
Dep. Variable:          Clarithromycin   R-squared (uncentered):                   0.567
Model:                             OLS   Adj. R-squared (uncentered):              0.561
Method:                  Least Squares   F-statistic:                              38.30
Date:                 Tue, 11 Feb 2025   Prob (F-statistic):                    8.93e-26
Time:                         10:18:47   Log-Likelihood:                        -1481.0
No. Observations:                  297   AIC:                                      2970.
Df Residuals:                      293   BIC:                                      2985.
Df Model:                            4                                                  
Covariance Type:                   HAC                                                  
==============================================================================
                 coef    std err          z      P>|z|      [0.025      0.975]
------------------------------------------------------------------------------
SARSCoV2    1.557e-10   2.19e-11      7.092      0.000    1.13e-10    1.99e-10
RSV         5.257e-10   5.82e-10      0.903      0.367   -6.16e-10    1.67e-09
IAV         1.766e-09   4.06e-10      4.346      0.000     9.7e-10    2.56e-09
IBV         1.647e-09   7.36e-10      2.237      0.025    2.04e-10    3.09e-09
==============================================================================
Omnibus:                       27.514   Durbin-Watson:                   0.866
Prob(Omnibus):                  0.000   Jarque-Bera (JB):               38.983
Skew:                           0.629   Prob(JB):                     3.43e-09
Kurtosis:                       4.252   Cond. No.                         35.9
==============================================================================

Notes:
[1] R² is computed without centering (uncentered) since the model does not contain a constant.
[2] Standard Errors are heteroscedasticity and autocorrelation robust (HAC)
using 5 lags and without small sample correction
```

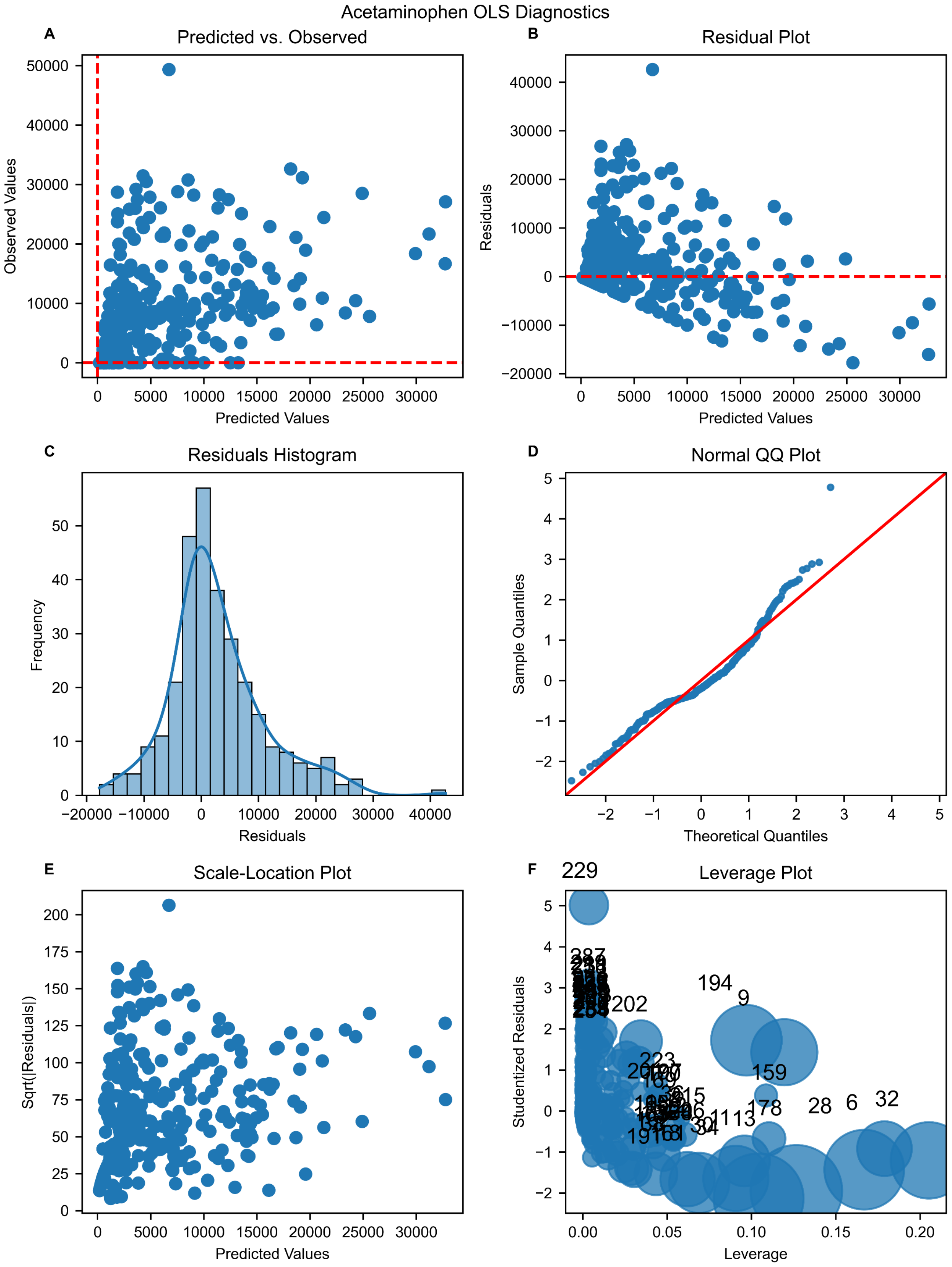

Figure SI 25: Diagnostic plots for the Ordinary Least Squares (OLS) regression analysis using 7-day centered median gene copy number loads of SARS-CoV-2, RSV, IAV, and IBV as predictors, with baseline-subtracted acetaminophen loads as the response variable. The figure includes: (A) Predicted vs. Observed Values, depicting the correlation between predicted and observed pharmaceutical loads; (B) Residual Plot, illustrating residuals against predicted values to assess homoscedasticity; (C) Residuals Histogram, showing the residual distribution with an overlaid kernel density estimate; (D) Normal Q-Q Plot, evaluating the normality of residuals; (E) Scale-Location Plot, revealing the spread of residuals to detect non-constant variance; and (F) Leverage Plot, highlighting influential data points using Cook's distance.

Table SI 8: Ordinary Least Squares (OLS) regression results using gene copy loads of SARS-COV-2, RSV, IAV and IBV as input as predicting variable and baseline-subtracted acetaminophen loads as the response variable. The time-series nature of the data is addressed using a heteroskedasticity- and autocorrelation-consistent (HAC) covariance matrix estimator, but the associated p-values and confidence intervals should still be interpreted with caution.

```
                                 OLS Regression Results                                
=======================================================================================
Dep. Variable:          Acetaminophen   R-squared (uncentered):                   0.507
Model:                            OLS   Adj. R-squared (uncentered):              0.500
Method:                 Least Squares   F-statistic:                              25.81
Date:                Tue, 11 Feb 2025   Prob (F-statistic):                    2.31e-18
Time:                        10:18:36   Log-Likelihood:                         -3150.4
No. Observations:                 300   AIC:                                      6309.
Df Residuals:                     296   BIC:                                      6324.
Df Model:                           4                                                  
Covariance Type:                  HAC                                                  
==============================================================================
                 coef    std err          z      P>|z|      [0.025      0.975]
------------------------------------------------------------------------------
SARSCoV2     2.68e-08   5.62e-09      4.767      0.000    1.58e-08    3.78e-08
RSV         2.759e-07   1.47e-07      1.872      0.061    -1.3e-08    5.65e-07
IAV         2.948e-07   1.05e-07      2.798      0.005    8.83e-08    5.01e-07
IBV         5.629e-07   1.61e-07      3.497      0.000    2.47e-07    8.78e-07
==============================================================================
Omnibus:                       50.597   Durbin-Watson:                   0.558
Prob(Omnibus):                  0.000   Jarque-Bera (JB):               90.895
Skew:                           0.926   Prob(JB):                     1.83e-20
Kurtosis:                       4.960   Cond. No.                         35.9
==============================================================================

Notes:
[1] R² is computed without centering (uncentered) since the model does not contain a constant.
[2] Standard Errors are heteroscedasticity and autocorrelation robust (HAC)
using 5 lags and without small sample correction
```

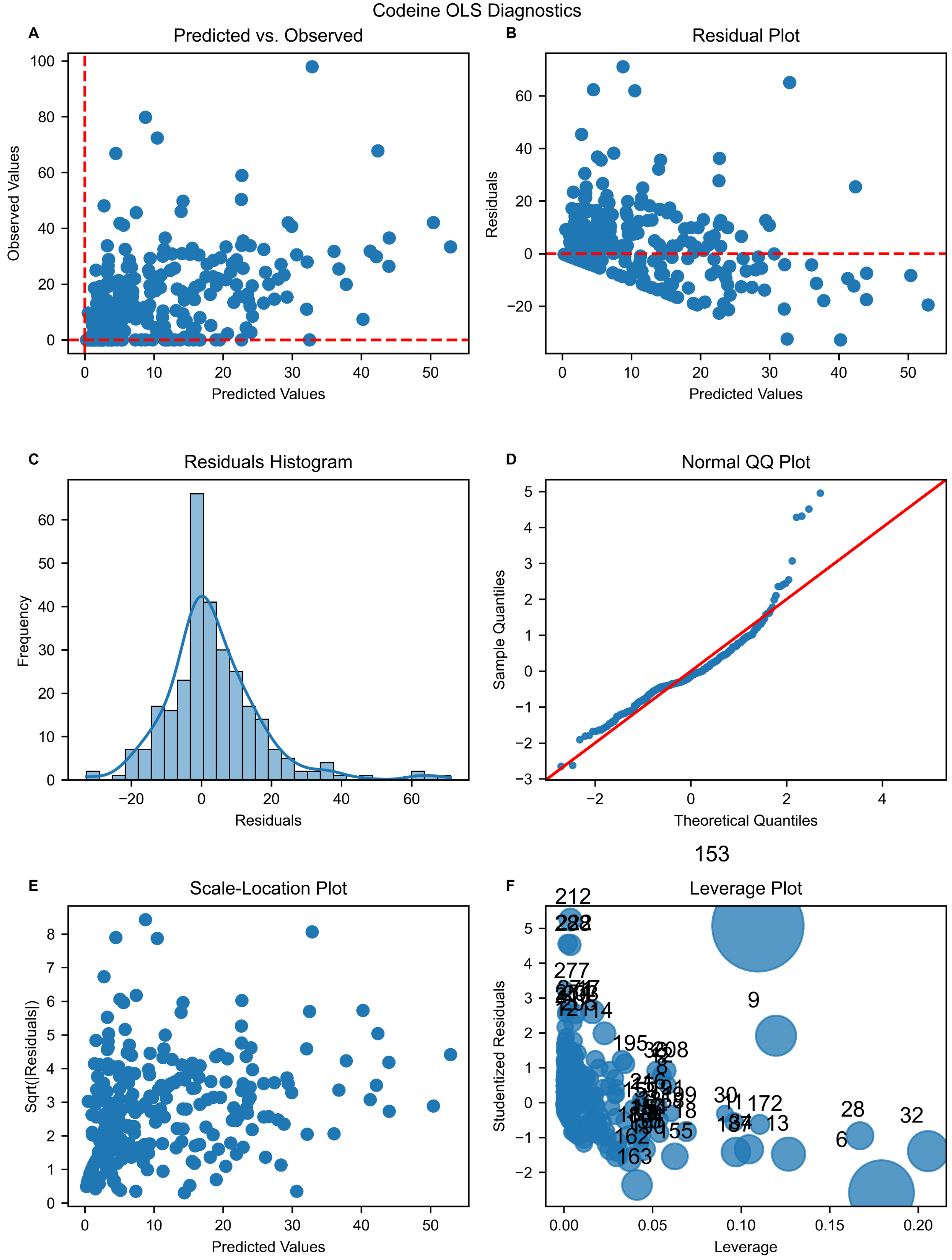

Figure SI 26: Diagnostic plots for the Ordinary Least Squares (OLS) regression analysis using 7-day centered median gene copy number loads of SARS-CoV-2, RSV, IAV, and IBV as predictors, with baseline-subtracted codeine loads as the response variable. The figure includes: (A) Predicted vs. Observed Values, depicting the correlation between predicted and observed pharmaceutical loads; (B) Residual Plot, illustrating residuals against predicted values to assess homoscedasticity; (C) Residuals Histogram, showing the residual distribution with an overlaid kernel density estimate; (D) Normal Q-Q Plot, evaluating the normality of residuals; (E) Scale-Location Plot, revealing the spread of residuals to detect non-constant variance; and (F) Leverage Plot, highlighting influential data points using Cook's distance.

Table SI 9: Ordinary Least Squares (OLS) regression results using gene copy loads of SARS-COV-2, RSV, IAV and IBV as input as predicting variable and baseline-subtracted codeine loads as the response variable. The time-series nature of the data is addressed using a heteroskedasticity- and autocorrelation-consistent (HAC) covariance matrix estimator, but the associated p-values and confidence intervals should still be interpreted with caution.

```
                            OLS Regression Results                            
==============================================================================
Dep. Variable:                Codeine   R-squared (uncentered):          0.524
Model:                            OLS   Adj. R-squared (uncentered):     0.518
Method:                 Least Squares   F-statistic:                     35.12
Date:                Tue, 11 Feb 2025   Prob (F-statistic):           6.91e-24
Time:                        10:18:13   Log-Likelihood:                -1186.5
No. Observations:                 292   AIC:                             2381.
Df Residuals:                     288   BIC:                             2396.
Df Model:                           4                                         
Covariance Type:                  HAC                                         
==============================================================================
                 coef    std err          z      P>|z|      [0.025      0.975]
------------------------------------------------------------------------------
SARSCoV2    4.012e-11    1.3e-11      3.077      0.002    1.46e-11    6.57e-11
RSV         8.911e-10   2.99e-10      2.980      0.003    3.05e-10    1.48e-09
IAV         2.939e-10   1.13e-10      2.600      0.009    7.24e-11    5.15e-10
IBV         7.986e-10   1.77e-10      4.515      0.000    4.52e-10    1.15e-09
==============================================================================
Omnibus:                      100.429   Durbin-Watson:                   1.374
Prob(Omnibus):                  0.000   Jarque-Bera (JB):              398.586
Skew:                           1.417   Prob(JB):                     2.81e-87
Kurtosis:                       7.973   Cond. No.                         35.9
==============================================================================

Notes:
[1] R² is computed without centering (uncentered) since the model does not contain a constant.
[2] Standard Errors are heteroscedasticity and autocorrelation robust (HAC) using 5 lags and without small sample correction
```

Ordinary least squares analyses of additional pharmaceuticals

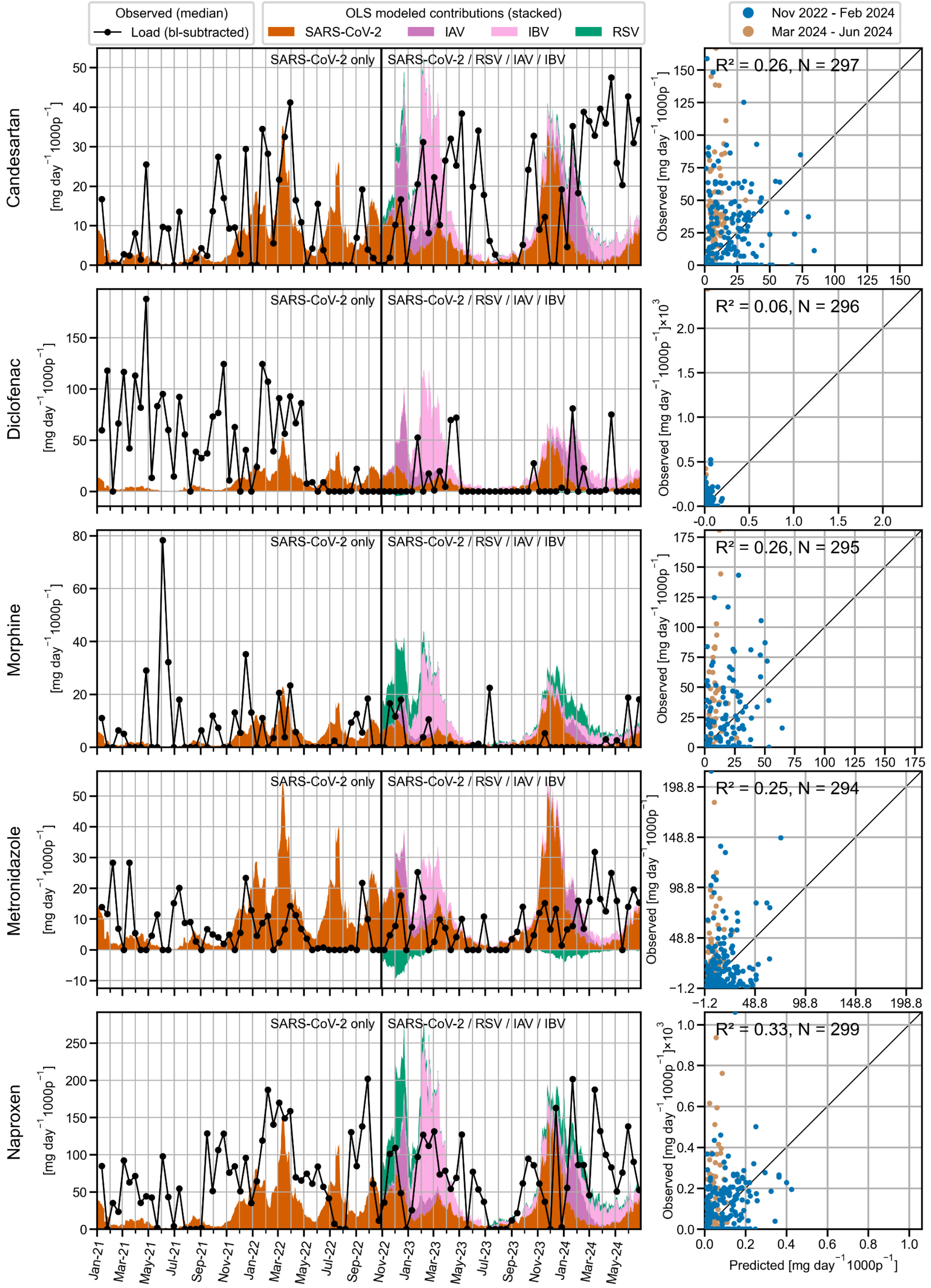

Figure SI 27: Modeled contributions of respiratory viruses to pharmaceutical loads. The left panel shows time series plots of observed baseline-subtracted pharmaceutical loads (black line) alongside the modeled contributions based on the median viral gene copy loads of SARS-CoV-2, RSV, IAV, and IBV, estimated using an ordinary least squares (OLS) model. The colored areas represent the stacked contributions of each virus to the overall baseline-subtracted pharmaceutical load. Baseline loads were determined from location-specific mean values during periods of low respiratory virus exposure (June 2021 and July to August 2023). For data before November 2022 (indicated by the vertical line), the model was retrospectively applied to SARS-CoV-2, as it was the only virus measured during those periods. The right panel presents scatter plots comparing observed versus predicted baseline-subtracted pharmaceutical loads for two distinct time periods: November 2022 to February 2024 (blue), March 2024 to June 2024 (orange). Each scatter plot includes the uncentered coefficient of determination (R^2) and the number of observations (N). The diagonal black line represents the 1:1 line, indicating perfect agreement between observed and predicted values.

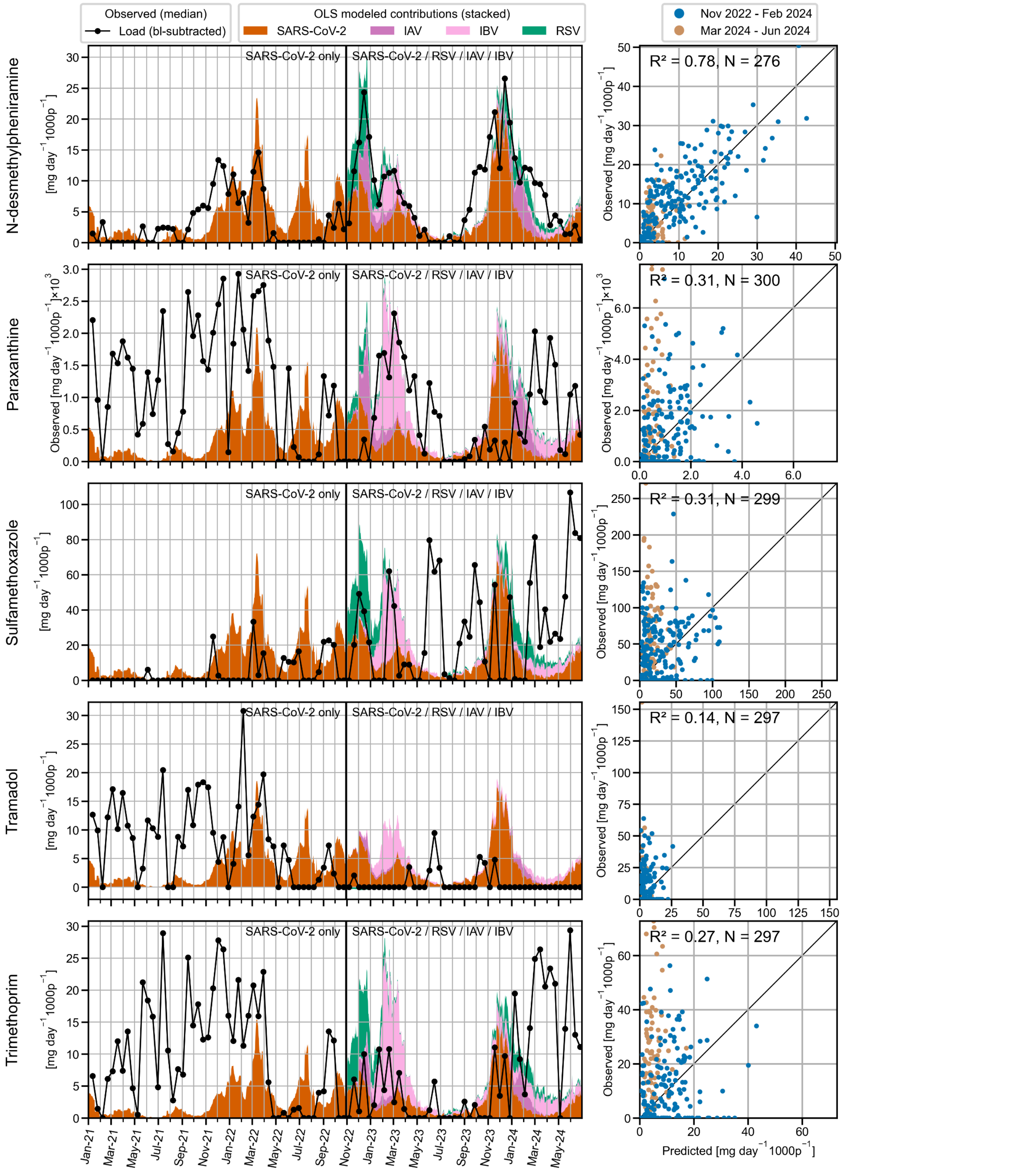

Figure SI 28: Modeled contributions of respiratory viruses to pharmaceutical loads. The left panel shows time series plots of observed baseline-subtracted pharmaceutical loads (black line) alongside the modeled contributions based on the median viral gene copy loads of SARS-CoV-2, RSV, IAV, and IBV, estimated using an ordinary least squares (OLS) model. The colored areas represent the stacked contributions of each virus to the overall baseline-subtracted pharmaceutical load. Baseline loads were determined from location-specific mean values during periods of low respiratory virus exposure (June 2021 and July to August 2023). For data before November 2022 (indicated by the vertical line), the model was retrospectively applied to SARS-CoV-2, as it was the only virus measured during those periods. The right panel presents scatter plots comparing observed versus predicted baseline-subtracted pharmaceutical loads for two distinct time periods: November 2022 to February 2024 (blue), March 2024 to June 2024 (orange). Each scatter plot includes the uncentered coefficient of determination (R^2) and the number of observations (N). The diagonal black line represents the 1:1 line, indicating perfect agreement between observed and predicted values.

Ordinary least squares analyses with SARS-COV-2 wastewater loads as only predicting variable

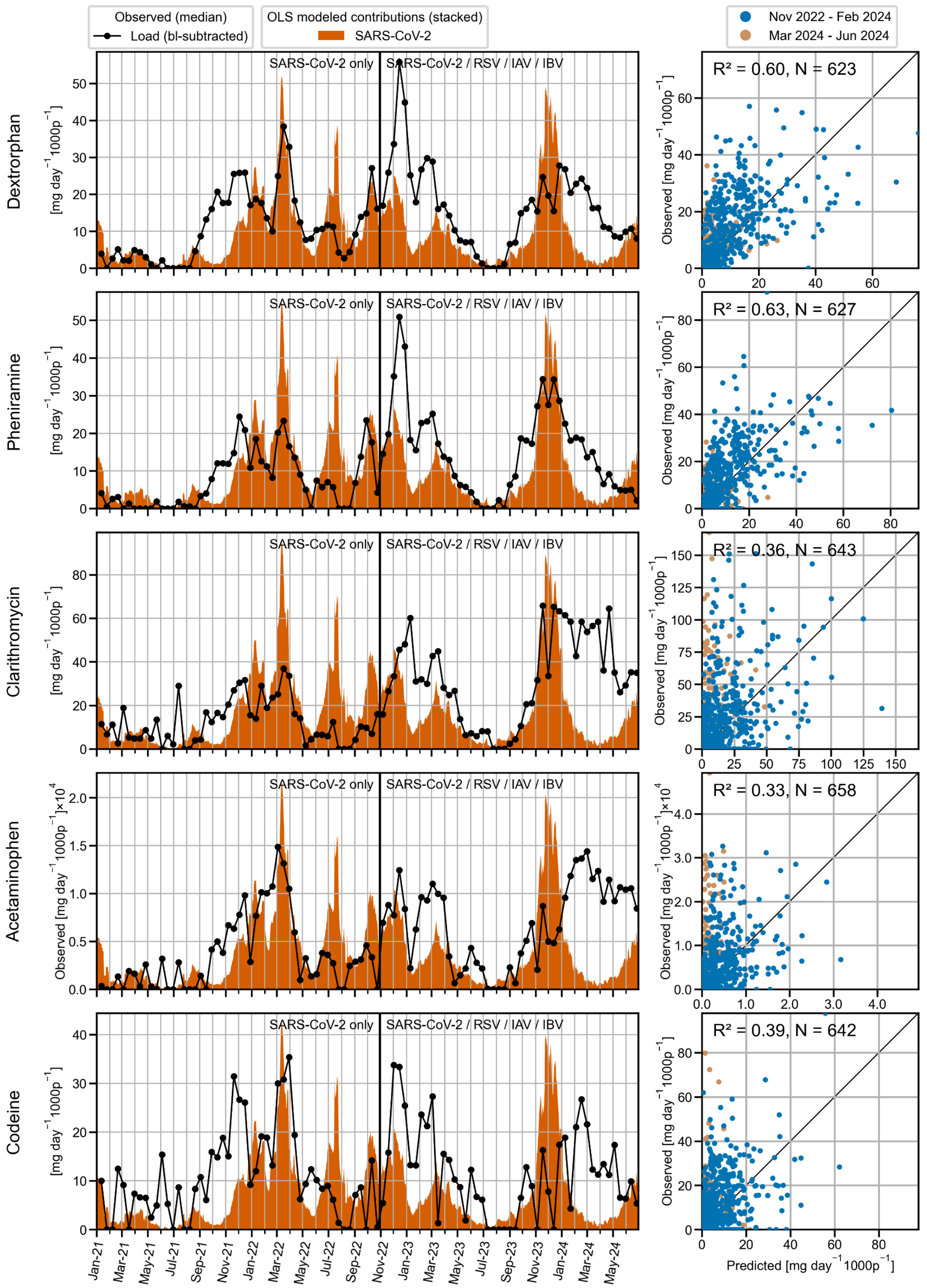

Figure SI 29: Modeled contributions of respiratory viruses to pharmaceutical loads. The left panel shows time series plots of observed baseline-subtracted pharmaceutical loads (black line) alongside the modeled contributions based on the median viral gene copy loads of SARS-CoV-2, estimated using an ordinary least squares (OLS) model. The colored areas represent the stacked contributions of each virus to the overall baseline-subtracted pharmaceutical load. Baseline loads were determined from location-specific mean values during periods of low respiratory virus exposure (June 2021 and July to August 2023). The right panel presents scatter plots comparing observed versus predicted baseline-subtracted pharmaceutical loads for two distinct time periods: November 2022 to February 2024 (blue), March 2024 to June 2024 (orange). Each scatter plot includes the uncentered coefficient of determination (R^2) and the number of observations (N). The diagonal black line represents the 1:1 line, indicating perfect agreement between observed and predicted values.

SI 2.4.2 **Ordinary least squares analyses with clinical cases as predicting variables**

To investigate whether the temporal occurrence of additional respiratory viruses recorded in the Swiss Sentinel Surveillance System (Sentinella) could explain unexplained gaps in pharmaceutical consumption, we conducted a series of separate OLS analyses. Initially, these analyses utilized Sentinel data for SARS-CoV-2, RSV, IAV, and IBV, which were also measured in wastewater. Subsequently, we expanded these analyses to include case data for either rhinovirus, an 'others' category (comprising seasonal human coronaviruses (HCoVs), bocavirus, human metapneumovirus (hMPV), and parainfluenza viruses (types 1-4)), or negative test results. The inclusion of negative test results aimed to identify potential triggers of acute respiratory infection (ARI) or influenza-like illness (ILI) symptoms in cases where no respiratory virus was detected. All analyses were based on median baseline-subtracted pharmaceutical loads, with Sentinella providing the national-level clinical case data.

OLS analysis with SARS-COV-2, RSV, IAV, IBV cases as predictors

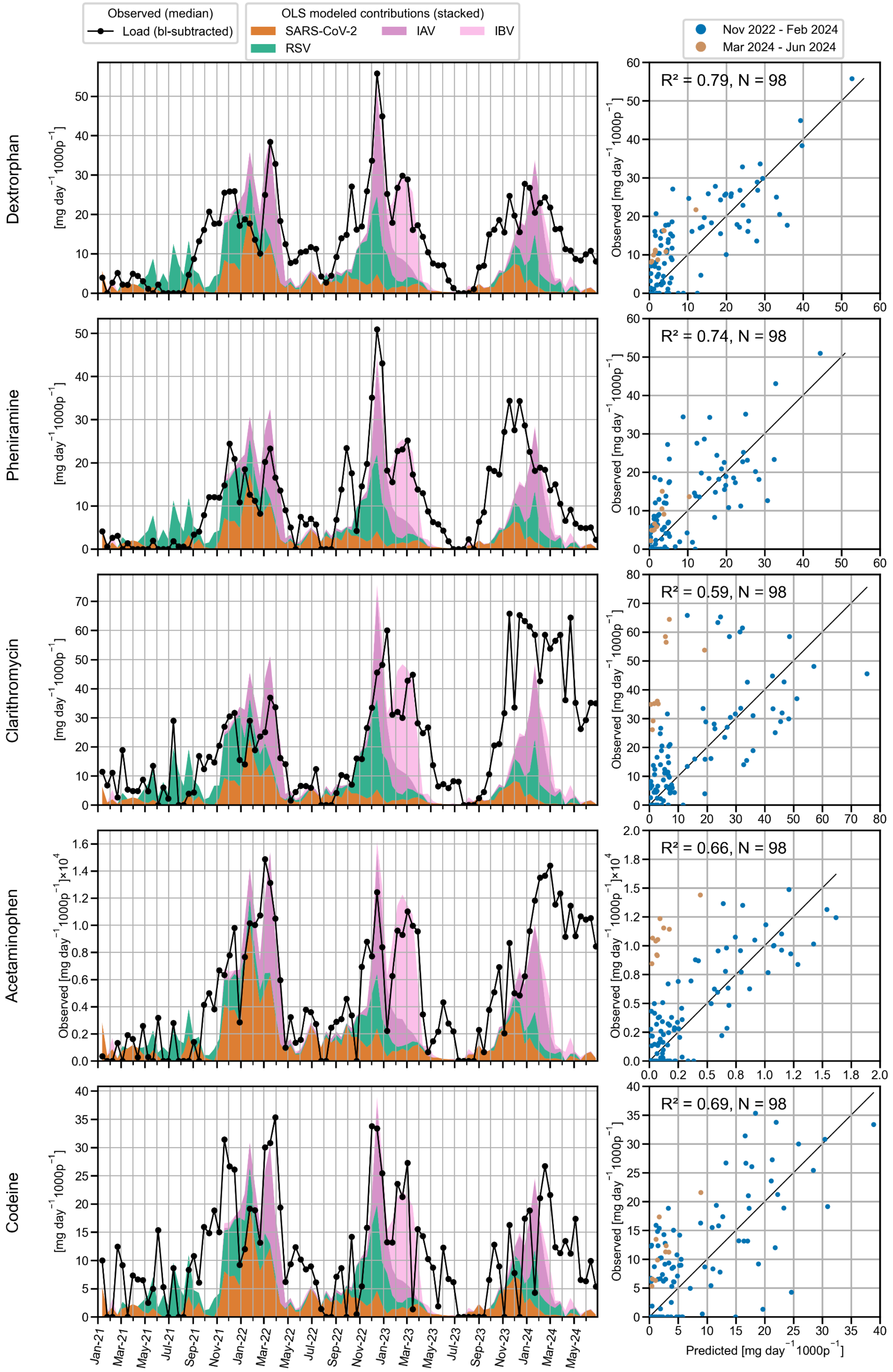

Figure SI 30: Modeled contributions of respiratory viruses to pharmaceutical loads. The left panel shows time series plots of observed median baseline-subtracted pharmaceutical loads (black line), alongside the modeled contributions of respiratory viruses, estimated using an ordinary least squares (OLS) model based on Sentinel system case data. The colored areas represent the stacked contributions of each virus to the overall baseline-subtracted pharmaceutical load. Baseline levels were determined using location-specific mean values during periods of minimal respiratory virus activity (June 2021 and July–August 2023). The right panel presents scatter plots comparing observed versus predicted baseline-subtracted pharmaceutical loads, with the uncentered coefficient of determination (R^2) and the number of observations (N). The diagonal black line represents the 1:1 line, denoting perfect agreement between observed and predicted values.

OLS Analysis with rhinovirus cases as an additional predictor

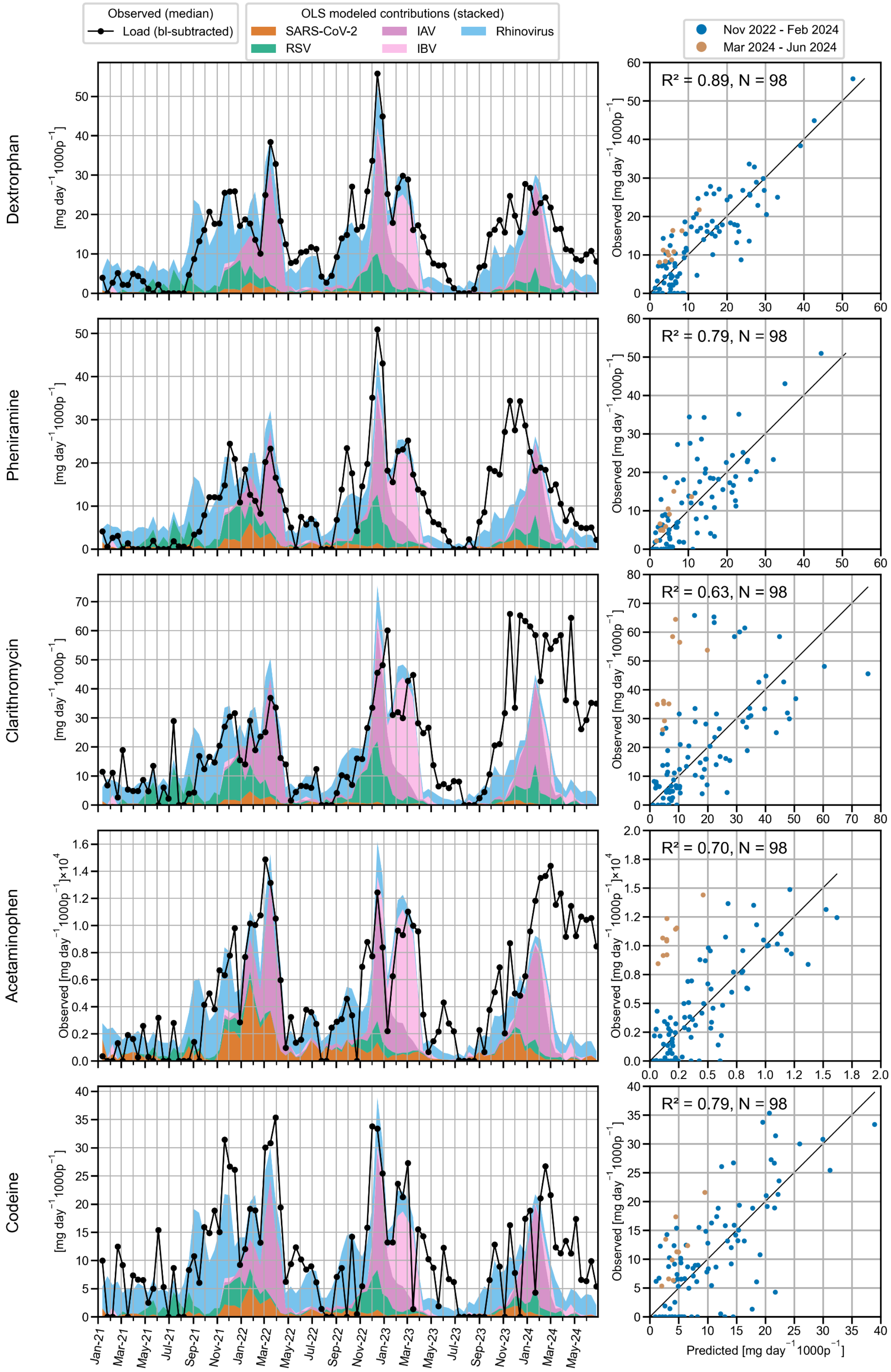

Figure SI 31: Modeled contributions of respiratory viruses to pharmaceutical loads. The left panel shows time series plots of observed median baseline-subtracted pharmaceutical loads (black line), alongside the modeled contributions of respiratory viruses, estimated using an ordinary least squares (OLS) model based on Sentinel system case data. The colored areas represent the stacked contributions of each virus to the overall baseline-subtracted pharmaceutical load. Baseline levels were determined using location-specific mean values during periods of minimal respiratory virus activity (June 2021 and July–August 2023). The right panel presents scatter plots comparing observed versus predicted baseline-subtracted pharmaceutical loads, with the uncentered coefficient of determination (R^2) and the number of observations (N). The diagonal black line represents the 1:1 line, denoting perfect agreement between observed and predicted values.

OLS Analysis with "Others" Category as an Additional Predictor

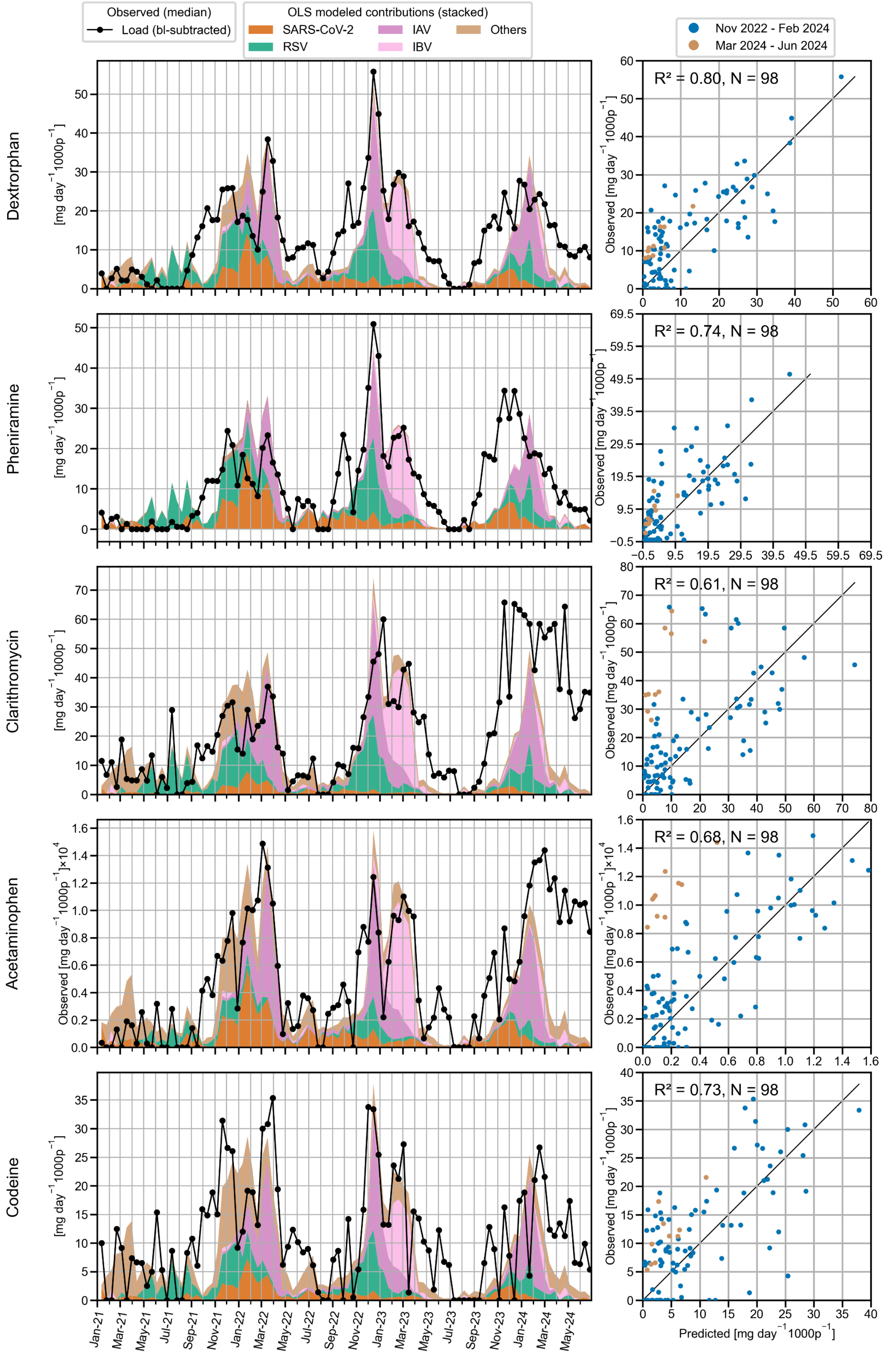

Figure SI 32: Modeled contributions of respiratory viruses to pharmaceutical loads. The left panel shows time series plots of observed median baseline-subtracted pharmaceutical loads (black line), alongside the modeled contributions of respiratory viruses, estimated using an ordinary least squares (OLS) model based on Sentinel system case data. The colored areas represent the stacked contributions of each virus to the overall baseline-subtracted pharmaceutical load. Baseline levels were determined using location-specific mean values during periods of minimal respiratory virus activity (June 2021 and July–August 2023). The right panel presents scatter plots comparing observed versus predicted baseline-subtracted pharmaceutical loads, with the uncentered coefficient of determination (R^2) and the number of observations (N). The diagonal black line represents the 1:1 line, denoting perfect agreement between observed and predicted values.

Including the "Others" category in the OLS modeling results in a negative coefficient for pheniramine, which is not physically meaningful and suggests a low explanatory power of this predictor.

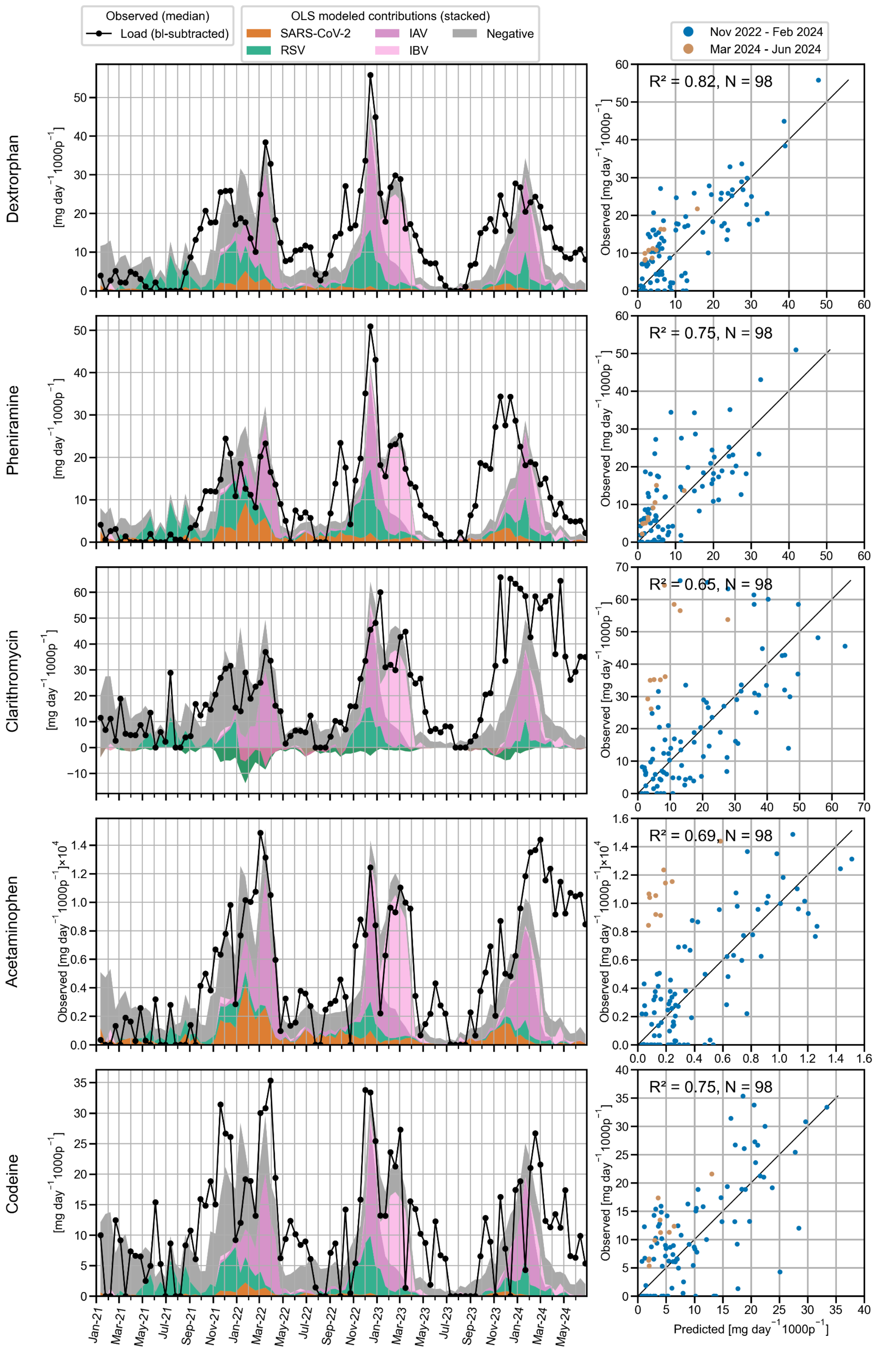

Figure SI 33: Modeled contributions of respiratory viruses to pharmaceutical loads. The left panel shows time series plots of observed median baseline-subtracted pharmaceutical loads (black line), alongside the modeled contributions of respiratory viruses, estimated using an ordinary least squares (OLS) model based on Sentinel system case data. The colored areas represent the stacked contributions of each virus to the overall baseline-subtracted pharmaceutical load. Baseline levels were determined using location-specific mean values during periods of minimal respiratory virus activity (June 2021 and July–August 2023). The right panel presents scatter plots comparing observed versus predicted baseline-subtracted pharmaceutical loads, with the uncentered coefficient of determination (R^2) and the number of observations (N). The diagonal black line represents the 1:1 line, denoting perfect agreement between observed and predicted values.

Including the "Negative" category in the OLS modeling results in a negative coefficient for clarithromycine, which is not physically meaningful and suggests a low explanatory power of this predictor.

SI 3 In-sample stability of chemical markers

Stability experiments demonstrated that the degradation of chemical compounds within the samples under study conditions is negligible. The chemicals remain stable over a 24-hour sampling period at 4°C and maintain concentrations within 80% of their original levels even when stored for more than a year at -20°C (long-term storage).

SI 3.1 **Sample preparation and analytical procedure**

The stability of the target analytes was evaluated in both spiked wastewater and Evian mineral water across different storage temperatures (-20°C, 4°C, and 22°C).
A 24-hour composite influent sample from Zurich Werdhölzli WWTP, collected under dry weather conditions on 28.03.2022, was used to represent the wastewater matrix. This sample was stored in a muffled 1000 mL Schott DURAN borosilicate glass bottle (Kisker Biotech) at 4°C in the dark for four days before the experiment commenced. Prior to spiking, the bottle was gently shaken and allowed to sit for 1.5 hours to permit the larger particles to settle. 700 mL of the supernatant was then transferred to a muffled bulkhead bottle, where it was spiked with an ethanol-based mix (1.7 mL) to reach a final concentration of 1000 ng/L.
After shaking, the bottle was left to equilibrate for 2 hours. Following equilibration, 20 mL of the supernatant was withdrawn to measure the pH (SevenMulti, Mettler Toledo), and the remaining solution was aliquoted into muffled 1.5 mL MS glass vials (BGB Analytik). The same steps were followed to prepare the Evian mineral water samples.
At the start of the experiment, aliquots were incubated at their designated temperatures. Following incubation, samples were thawed for 1 minute under lukewarm water (for frozen samples), spiked with 20 µL of a 60 µg/L ethanol-based isotope-labeled internal standard solution, shaken and vortexed, centrifuged at room temperature for 10 minutes at 279.5 RCF (5427 R, Eppendorf centrifuge), and analyzed immediately. All samples were examined using large-volume direct injection reversed-phase LC-HRMS, as detailed in the methods section.
Replicates were analyzed at the initial measurement (t0) for samples from the same matrix. Although most time points involved only one measurement per sample condition, replicates were occasionally measured, as detailed in the accompanying table (Table SI 10).
The procedure closely follows the method used in [1] for determining in-sample stability.

Table SI 10: Experimental conditions of in-sample stability experiment and number of samples measured per condition and time point

	Wastewater Influent			Evian mineral water	
Time point	22°C	4°C	-20°C	°4C	-20°C
0h	3	3	3	3	3
1d	1	1	1	1	1
2d	1	1	1	1	1
3d	1	1	1	1	1
7d / 8d	2	2	2	2	2
9d / 10d	2	2	2	2	2
14d / 15d	2	2	2	2	2
20d	1	1	1	1	1
up to 286d	-	-	1	-	1

Table SI 11: Result of pH measurements of the stability experiment samples after spiking and at the end of experiment

Matrix	Temperature [°C]	pH	
		After spiking	End of experiment (t=20d)
Wastewater influent	22	7.6	7.6
	4	7.6	5.9
	-20	7.6	8.2
Evian mineral water	4	7.7	8.2
	-20	7.7	9.5

SI 3.2 **Data analysis**

The change in relative concentration over time was assessed by comparing the peak area ratio of each analyte to its corresponding internal standard (ISTD) for short-term in-sample stability. For long-term stability assessments, relative concentrations were used to account for potential variations in ISTD concentrations between the different ISTD mixes prepared over the course of several years. For each substance and condition, the average response ratio or concentration of the triplicate at the initial time point (t0) was normalized to 100%. Changes in relative concentration were subsequently calculated as a percentage of this initial area ratio or concentration, enabling the evaluation of degradation or formation over time.
To determine the degradation rate constant for each analyte in the specific matrix and under particular conditions, a non-linear pseudo first-order kinetic model was fitted to the data. The degradation process is described by Equation SI 7, where A_0 represents the initial response ratio or concentration, A is the response ratio or concentration at a given time point, k denotes the degradation rate constant, and t represents time (in days).

$$A = A_0 e^{-kt} \qquad (SI\ 7)$$

The parameters *A0* and *k* were optimized using Scipy in Python.

SI 3.3 **Stability results**

Key pharmaceutical markers dextrorphan, pheniramine, clarithromycin, acetaminophen, and codeine, exhibited consistent stability when stored at 22°C for 15 days and -20°C for 400 days in both Evian mineral water and wastewater (Figures SI32 - SI35). The relative concentrations of these markers remained within the 80% to 120% range, as described by pseudo-first-order kinetics models. This indicates that the conditions and durations used in the current study are unlikely to result in noticeable degradation, ensuring reliable data for these compounds. This observation

applies to most other markers tested. In contrast, antibiotics metronidazole and sulfamethoxazole showed notable degradation, particularly at 22°C in wastewater matrix. Additionally, morphine concentrations increased significantly in spiked wastewater at both 22°C and 4°C, and in Evian water at 22°C (Figures SI 32), likely due to the hydrolysis of co-spiked heroin and 6-acetylmorphine [2]. However, given the generally low levels of heroin and 6-acetylmorphine in real wastewater, this effect has limited impact on real-world measurements.

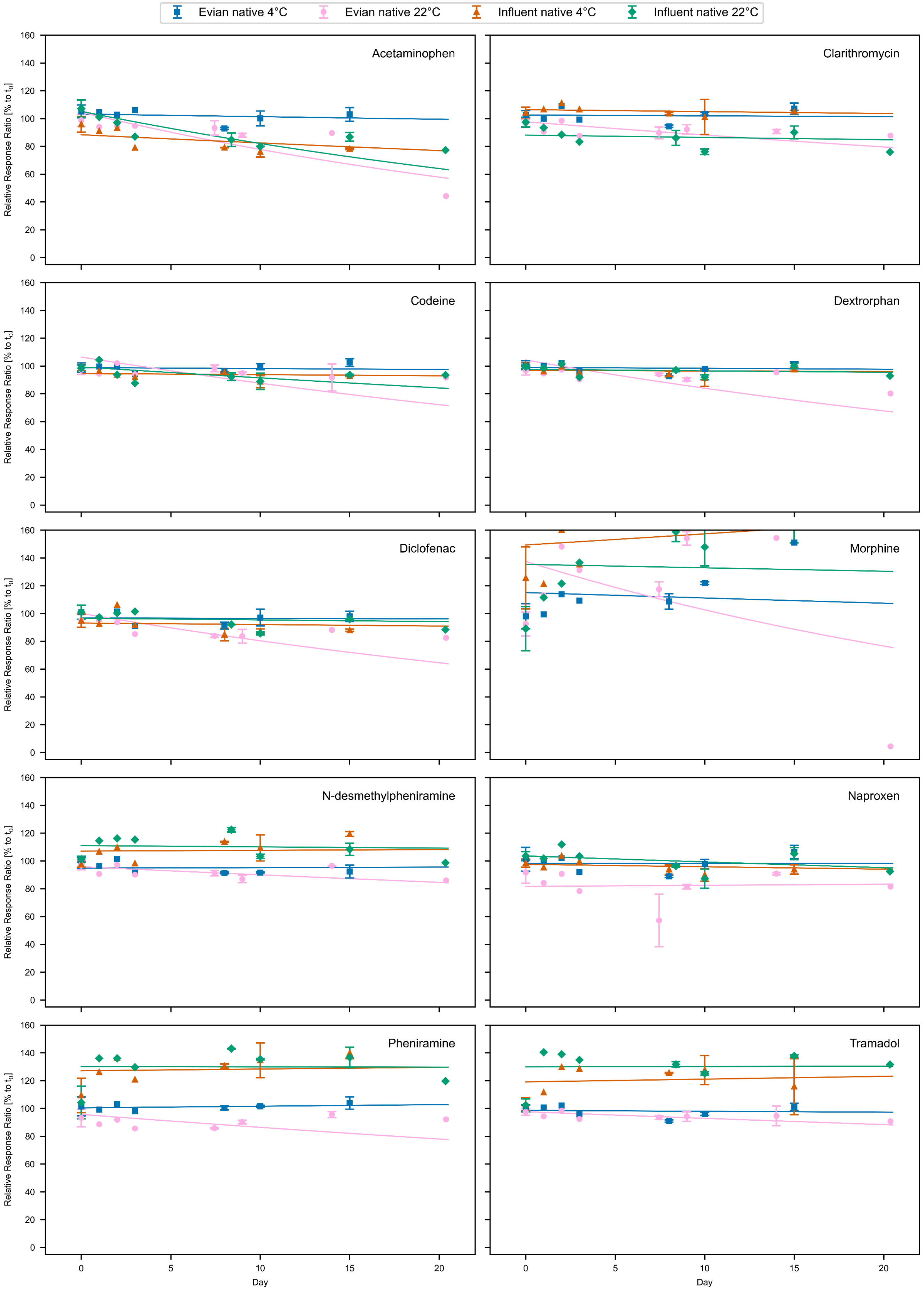

Figure SI 34: Stability of Chemicals in Wastewater Influent and Evian Mineral Water Incubated at 4°C and 22°C for 20 Days. The figure presents measured data points along with pseudo-first-order kinetic fits (lines). At time points with multiple measurements, data are presented as mean values with error bars indicating standard deviations.

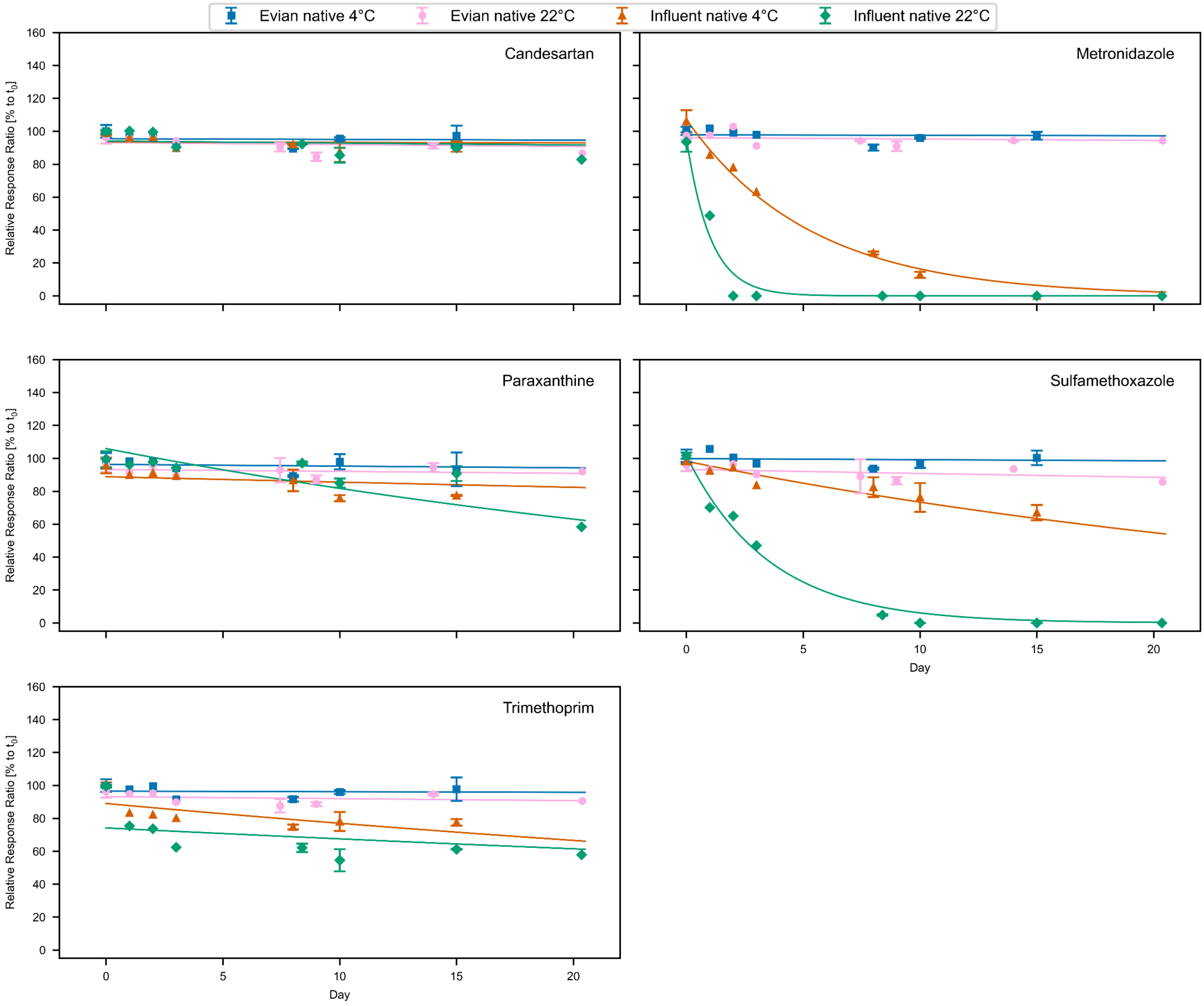

Figure SI 35: Stability of Chemicals in Wastewater Influent and Evian Mineral Water Incubated at 4°C and 22°C for 20 Days. The figure presents measured data points along with pseudo-first-order kinetic fits (lines). At time points with multiple measurements, data are presented as mean values with error bars indicating standard deviations.

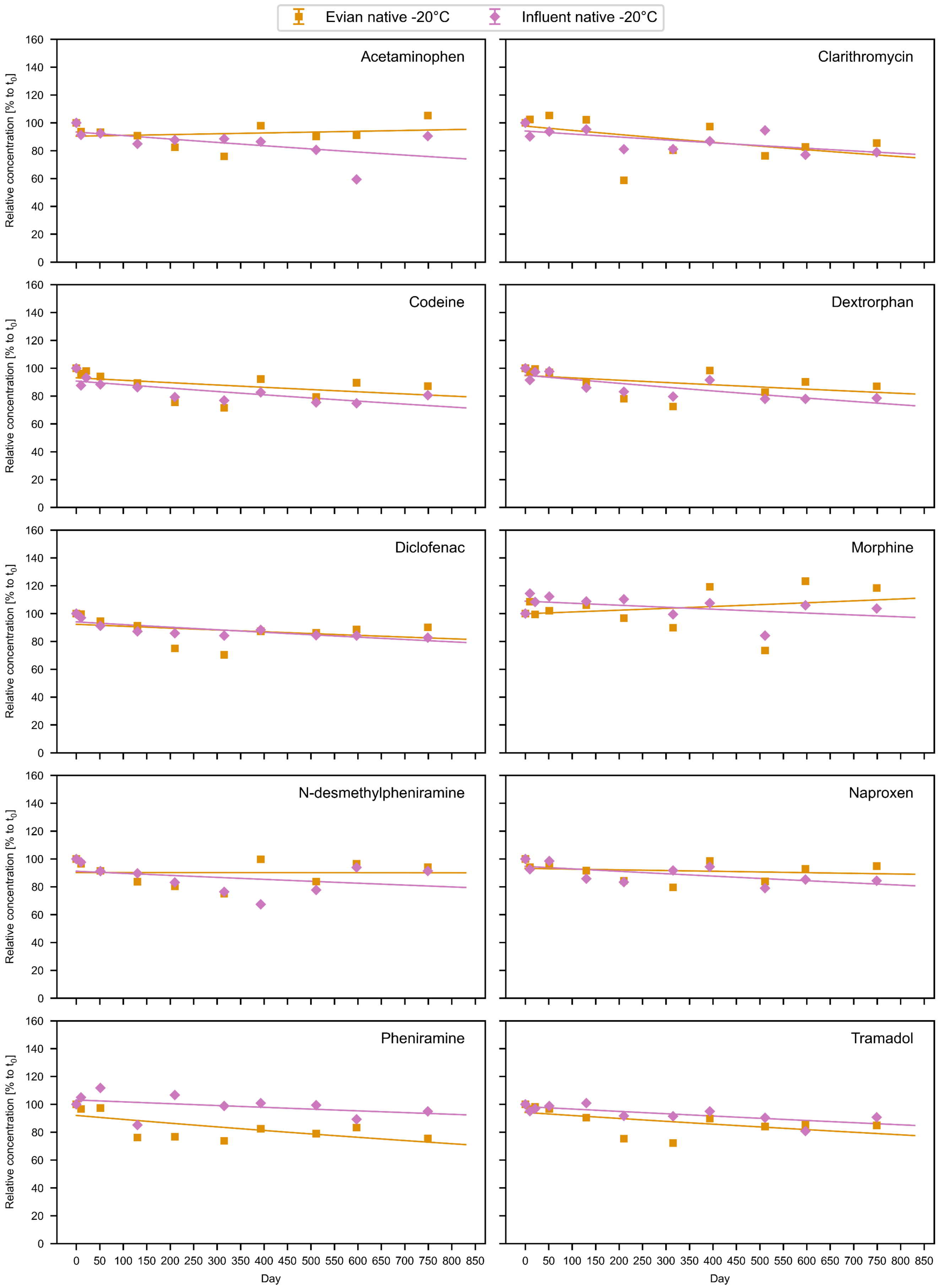

Figure SI 36: Long-term Stability of chemicals in Wastewater Influent and Evian Mineral Water Incubated at -20°C. The figure presents measured data points along with pseudo-first-order kinetic fits (lines). At time points with multiple measurements, data are presented as mean values with error bars indicating standard deviations.

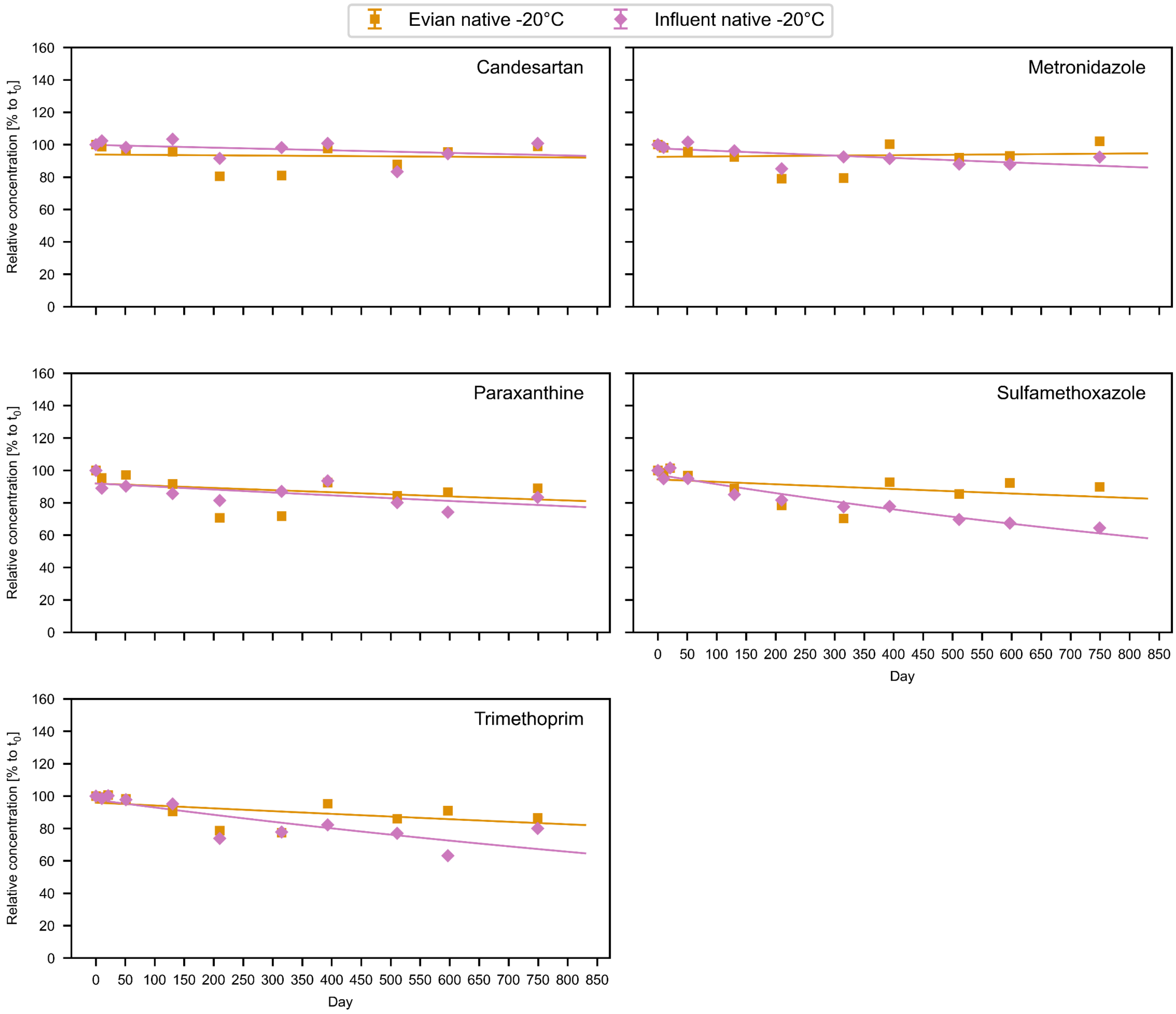

Figure SI 37: Long-term Stability of chemicals in Wastewater Influent and Evian Mineral Water Incubated at -20°C. The figure presents measured data points along with pseudo-first-order kinetic fits (lines). At time points with multiple measurements, data are presented as mean values with error bars indicating standard deviations.

SI 4 Licensed pharmaceutical products in Switzerland containing target chemicals

Table SI 12: Licensed Pharmaceutical Products in Switzerland Containing Dextromethorphan or Pheniramine, as Listed by the Swiss Agency for Therapeutic Products (Swissmedic)[3]. For products with multiple packaging sizes available, only a single option is listed.

Compound	Product	Package Size	Unit	Composition	Dispensing Category Medicine	Application Area
Dextromethorphan	GEM Antitussivum, Sirup	200	ml	dextromethorphanum 6.25 mg ut dextromethorphani hydrobromidum, …, excipiens ad solutionem pro 5 ml.	B	Husten
Dextromethorphan	SUN STORE Dextromethorphan, Hustensirup	200	ml	dextromethorphanum 25 mg ut dextromethorphani hydrobromidum, …	B	Husten
Dextromethorphan	Amavita Dextromethorphan -N, Hustensirup	200	ml	dextromethorphanum 25 mg ut dextromethorphani hydrobromidum, …	B	Husten
Dextromethorphan	Amavita Dextromethorphan, Hustentabletten	16	Tablette(n)	dextromethorphanum 25 mg ut dextromethorphani hydrobromidum,…	B	Husten
Dextromethorphan	Coop Vitality Dextromethorphan -N, Hustensirup	200	ml	dextromethorphanum 25 mg ut dextromethorphani hydrobromidum, ..	B	Husten
Dextromethorphan	Coop Vitality Dextromethorphan, Hustentabletten	16	Tablette(n)	dextromethorphanum 25 mg ut dextromethorphani hydrobromidum, ..	B	Husten
Dextromethorphan	Bisolvon Dextromethorphan, Sirup	200	Flasche(n)	dextromethorphani hydrobromidum 10 mg corresp. dextromethorphanum 7.3 mg, …	B	trockener Reizhusten
Dextromethorphan	Irotussin Antitussivum, Sirup	200	ml	dextromethorphanum 6.25 mg ut dextromethorphani hydrobromidum,	B	Husten
Dextromethorphan	Calmesin-Mepha, Sirup	90	ml	dextromethorphanum 11.5 mg ad resinam adsorbatum corresp., dextromethorphani hydrobromidum 15 mg, ..	B	Husten
Dextromethorphan	Pulmofor, sirop	200	ml	dextromethorphani hydrobromidum 25 mg corresp. dextromethorphanum 18.3 mg, ..	B	Toux, particulièrement la toux sèche irritative
Dextromethorphan	Pulmofor retard, capsules	10	Kapsel(n)	dextromethorphani hydrobromidum 50 mg corresp. dextromethorphanum 36.6 mg, ..	B	Toux, particulièrement la toux irritative
Dextromethorphan	Pretuval Grippe & Erkältung, Filmtabletten	20	Tablette(n)	dextromethorphani hydrobromidum 20 mg corresp. dextromethorphanum 14.66 mg, ..	D	Erkältungskrankheiten mit Husten, Fieber und Schmerzen
Dextromethorphan	Bexin Hustentabletten, Filmtabletten	24	Tablette(n)	dextromethorphanum 25 mg ut dextromethorphani hydrobromidum, ..	B	Husten
Dextromethorphan	Pretuval Grippe & Erkältung C, Brausetabletten	10	Tablette(n)	dextromethorphani hydrobromidum 20 mg corresp. dextromethorphanum 14.66 mg, ..	D	Erkältungskrankheiten mit Husten, Fieber und Schmerzen
Dextromethorphan	Calmerphan-L, Sirup	90	ml	dextromethorphanum 11.5 mg ad resinam adsorbatum corresp., dextromethorphani hydrobromidum 15 mg, ..	B	Husten
Dextromethorphan	Bexin, Hustentropfen	20	ml	dextromethorphanum 20.81 mg ut dextromethorphani hydrobromidum, ..	B	Husten
Dextromethorphan	Bexin, Hustensirup	200	ml	dextromethorphanum 25 mg ut dextromethorphani hydrobromidum, ..	B	Husten
Dextromethorphan	Emedrin N, Sirup	150	ml	dextromethorphanum 12.5 mg ut dextromethorphani hydrobromidum 17.075 mg, ..	B	Husten, insbesondere trockener Reizhusten
Pheniramine	SUN STORE Flumol Grippe, Pulver zur Herstellung einer Lösung zum Einnehmen	12	Beutel	pheniramini maleas 20 mg, ..	D	Symptomatische Behandlung bei Erkältungskrankheiten
Pheniramine	NeoCitran Grippe / Erkältung für Erwachsene, Pulver zur Herstellung einer Lösung zum Einnehmen	12	Beutel	pheniramini maleas 20 mg..	D	Symptomatische Behandlung bei Erkältungskrankheiten
Pheniramine	neotylol Grippe, Pulver zur Herstellung einer Lösung zum Einnehmen	12	Beutel	pheniramini maleas 20 mg, ..	D	Symptomatische Behandlung bei Erkältungskrankheiten
Pheniramine	Dafalgan Grippal, Granulat zur Herstellung einer Lösung zum Einnehmen	12	Beutel	pheniramini maleas 25 mg, ..	D	Symptomatische Behandlung bei Erkältungskrankheiten
Pheniramine	Amavita Flumol Grippe, Pulver zur Herstellung einer Lösung zum Einnehmen	12	Beutel	pheniramini maleas 20 mg, ..	D	Symptomatische Behandlung bei Erkältungskrankheiten

Licensed products containing pheniramine are classified under dispensing category D, meaning they are available over-the-counter. Similarly, some dextromethorphan-containing products are also categorized as D, though the majority are classified under dispensing category B, which necessitates a medical prescription (Table SI 12).
Information on pharmaceutical products containing additional analyzed chemicals is included in the data package accompanying this publication.

SI 5 References

1. Baumgartner S, Salvisberg M, Clot B, et al. Relationship between antihistamine residues in wastewater and airborne pollen concentrations: Insights into population-scale pollinosis response. *Science of The Total Environment*. 2025;964:178515. doi:10.1016/J.SCITOTENV.2025.178515

2. Lin X, Choi PM, Thompson J, et al. Systematic Evaluation of the In-Sample Stability of Selected Pharmaceuticals, Illicit Drugs, and Their Metabolites in Wastewater. *Environ Sci Technol*. 2021;55(11):7418-7429. doi:10.1021/acs.est.1c00396

3. Swissmedic. Accessed August 20, 2024. https://www.swissmedic.ch/swissmedic/en/home/services/medicinal-product-information.html
