# Supplementary material for: Insights into respiratory illness at the population level through parallel analysis of pharmaceutical and viral markers in wastewater
Source: Nat Water. 2025 May 14;3(5):580–9. doi: 10.1038/s44221-025-00437-4 (PMC12098119; doi:10.1038/s44221-025-00437-4)
Supplement: Supplementary file 2 — Reporting Summary [file 44221_2025_437_MOESM2_ESM.pdf]

nature portfolio

Corresponding author(s): Christoph Ort

Last updated by author(s): Mar 25, 2025

Reporting Summary

Nature Portfolio wishes to improve the reproducibility of the work that we publish. This form provides structure for consistency and transparency in reporting. For further information on Nature Portfolio policies, see our Editorial Policies and the Editorial Policy Checklist.

Statistics

For all statistical analyses, confirm that the following items are present in the figure legend, table legend, main text, or Methods section.

n/a	Confirmed	
☒	☐	The exact sample size (*n*) for each experimental group/condition, given as a discrete number and unit of measurement
☐	☒	A statement on whether measurements were taken from distinct samples or whether the same sample was measured repeatedly
☒	☐	The statistical test(s) used AND whether they are one- or two-sided<br>*Only common tests should be described solely by name; describe more complex techniques in the Methods section.*
☒	☐	A description of all covariates tested
☐	☒	A description of any assumptions or corrections, such as tests of normality and adjustment for multiple comparisons
☐	☒	A full description of the statistical parameters including central tendency (e.g. means) or other basic estimates (e.g. regression coefficient) AND variation (e.g. standard deviation) or associated estimates of uncertainty (e.g. confidence intervals)
☐	☒	For null hypothesis testing, the test statistic (e.g. *F*, *t*, *r*) with confidence intervals, effect sizes, degrees of freedom and *P* value noted<br>*Give P values as exact values whenever suitable.*
☒	☐	For Bayesian analysis, information on the choice of priors and Markov chain Monte Carlo settings
☒	☐	For hierarchical and complex designs, identification of the appropriate level for tests and full reporting of outcomes
☐	☒	Estimates of effect sizes (e.g. Cohen's *d*, Pearson's *r*), indicating how they were calculated

Our web collection on statistics for biologists contains articles on many of the points above.

Software and code

Policy information about availability of computer code

Data collection	TraceFinder 5.1 Thermo Fisher standard software for HRMS measurements (for pharmaceutical analyses); Crystal Miner Software version 4.0 Stilla Technologies (for RNA quantification)
Data analysis	Python, the whole code and computational environment are provided in an institutional data repository as specified in the data availability statement.

For manuscripts utilizing custom algorithms or software that are central to the research but not yet described in published literature, software must be made available to editors and reviewers. We strongly encourage code deposition in a community repository (e.g. GitHub). See the Nature Portfolio guidelines for submitting code & software for further information.

Data

Policy information about availability of data

All manuscripts must include a data availability statement. This statement should provide the following information, where applicable:

- Accession codes, unique identifiers, or web links for publicly available datasets
- A description of any restrictions on data availability
- For clinical datasets or third party data, please ensure that the statement adheres to our policy

The dataset, along with the corresponding analysis and visualization scripts, is available in Eawag's Research Data Institutional Collection (ERIC-open) at https://doi.org/10.25678/000D6F.

Research involving human participants, their data, or biological material

Policy information about studies with human participants or human data. See also policy information about sex, gender (identity/presentation), and sexual orientation and race, ethnicity and racism.

Reporting on sex and gender	NA
Reporting on race, ethnicity, or other socially relevant groupings	NA
Population characteristics	NA
Recruitment	We analyzed raw wastewater from urban catchments (all >30'000p) to which the entire population present in the catchment contributed anonymously.
Ethics oversight	NA

Note that full information on the approval of the study protocol must also be provided in the manuscript.

Field-specific reporting

Please select the one below that is the best fit for your research. If you are not sure, read the appropriate sections before making your selection.

☐ Life sciences ☐ Behavioural & social sciences ☒ Ecological, evolutionary & environmental sciences

For a reference copy of the document with all sections, see nature.com/documents/nr-reporting-summary-flat.pdf

Ecological, evolutionary & environmental sciences study design

All studies must disclose on these points even when the disclosure is negative.

Study description	Analyses of raw wastewater from large catchments (all >30'000p) for pharmaceutical residues and viral RNA fragments of respiratory viruses.
Research sample	24-h composite raw wastewater samples representing the excreta from the entire population present in the urban drainage catchment
Sampling strategy	Number of sites: 10 wastewater treatment plants in Switzerland covering ~23% of the Swiss population.<br>Raw wastewater for pharmaceutical residue analyses: every 13th day (7 samples per quarter, each weekday once) to cover intra-week and seasonal variation [for one location also daily values in SI]<br>Raw wastewater for viral RNA fragments: initially daily samples, from July 2023 onwards 5 samples per week.
Data collection	Flow data and size of residential population: provided by wastewater treatment plant operators.<br>Concentrations for pharmaceutical residues and viral RNA fragments with instruments and software specified above.
Timing and spatial scale	January 2021 to June 2024; the 10 locations are large wastewater treatment plants and represent the different regions in Switzerland
Data exclusions	As specified in the manuscript:<br>A small number of days during rain events were excluded, when the inflow volume at WWTPs exceeded an empirically determined location-specific maximum threshold (for more details see SI).<br>For Lugano WWTP, three data points were excluded due to unusually high loads, likely from sewer disposal or industrial sources
Reproducibility	We did not perform experiments, we observed and investigated parameters in raw wastewater resulting in time series of pharmaceutical residues and viral RNA fragments.
Randomization	NA (raw wastewater from large populations)
Blinding	NA (raw wastewater from large populations)

Did the study involve field work? ☐ Yes ☒ No

Reporting for specific materials, systems and methods

We require information from authors about some types of materials, experimental systems and methods used in many studies. Here, indicate whether each material, system or method listed is relevant to your study. If you are not sure if a list item applies to your research, read the appropriate section before selecting a response.

Materials & experimental systems

n/a	Involved in the study
☒	☐ Antibodies
☒	☐ Eukaryotic cell lines
☒	☐ Palaeontology and archaeology
☒	☐ Animals and other organisms
☒	☐ Clinical data
☒	☐ Dual use research of concern
☒	☐ Plants

Methods

n/a	Involved in the study
☒	☐ ChIP-seq
☒	☐ Flow cytometry
☒	☐ MRI-based neuroimaging

Plants

Seed stocks	NA
Novel plant genotypes	NA
Authentication	NA
